# Combined HDAC8 and checkpoint kinase inhibition induces tumor-selective synthetic lethality in preclinical models

**DOI:** 10.1172/JCI165448

**Published:** 2024-10-22

**Authors:** Ting-Yu Chang, Yan Yan, Zih-Yao Yu, Moeez Rathore, Nian-Zhe Lee, Hui-Ju Tseng, Li-Hsin Cheng, Wei-Jan Huang, Wei Zhang, Ernest R. Chan, Yulan Qing, Ming-Lun Kang, Rui Wang, Kelvin K. Tsai, John J. Pink, William E. Harte, Stanton L. Gerson, Sung-Bau Lee

**Affiliations:** 1 PhD Program in Drug Discovery and Development Industry, College of Pharmacy, Taipei Medical University, Taipei, Taiwan.; 2 Case Comprehensive Cancer Center and; 3 Department of Surgery, Case Western Reserve University (CWRU) School of Medicine, Cleveland, Ohio, USA.; 4 Laboratory of Advanced Molecular Therapeutics, Graduate Institute of Clinical Medicine, College of Medicine,; 5 Core Laboratory of Organoids Technology, Office of R&D,; 6 Graduate Institute of Pharmacognosy, College of Pharmacy, Taipei Medical University, Taipei, Taiwan.; 7 Institute for Computational Biology, CWRU School of Medicine, Cleveland, Ohio, USA.; 8 Genomics Research Center, Academia Sinica, Taipei, Taiwan.; 9 Office of Translation and Innovation and; 10 Department of Medicine, CWRU School of Medicine, Cleveland, Ohio, USA.

**Keywords:** Cell biology, Therapeutics, Cancer, Cell cycle, Genetic instability

## Abstract

The elevated level of replication stress is an intrinsic characteristic of cancer cells. Targeting the mechanisms that maintain genome stability to further increase replication stress and thus induce severe genome instability has become a promising approach for cancer treatment. Here, we identify histone deacetylase 8 (HDAC8) as a drug target whose inactivation synergized with the inhibition of checkpoint kinases to elicit substantial replication stress and compromise genome integrity selectively in cancer cells. We showed that simultaneous inhibition of HDAC8 and checkpoint kinases led to extensive replication fork collapse, irreversible cell-cycle arrest, and synergistic vulnerability in various cancer cells. The efficacy of the combination treatment was further validated in patient tumor–derived organoid (PDO) and xenograft mouse (PDX) models, providing important insights into patient-specific drug responses. Our data revealed that HDAC8 activity was essential for reducing the acetylation level of structural maintenance of chromosomes protein 3 (SMC3) ahead of replication forks and preventing R loop formation. HDAC8 inactivation resulted in slowed fork progression and checkpoint kinase activation. Our findings indicate that HDAC8 guards the integrity of the replicating genome, and the cancer-specific synthetic lethality between HDAC8 and checkpoint kinases provides a promising replication stress–targeting strategy for treating a broad range of cancers.

## Abstract 2

This study identifies a novel function of HDAC8 in guarding the integrity of replicating genome and a cancer-specific synthetic lethality between HDAC8 and checkpoint kinases.

## Graphical abstract

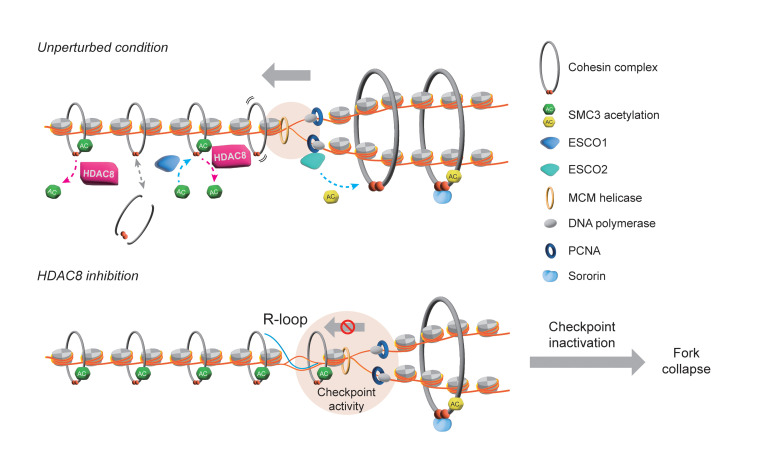

## Introduction

To maintain the integrity of the human genome, the genetic and epigenetic information in proliferating cells must be precisely duplicated during chromatin replication. Replication stress, defined as any impediment to genome replication, is considered a unique feature of cancer (1). Owing to uncontrolled cell proliferation driven by oncogenes, chromatin replication in cancer cells is often threatened in the face of numerous obstacles, such as deregulation of origin firing, shortage of replication building blocks, interference between replication and transcription, etc. (2). The common consequences of unresolved replication stress include stalling and collapse of DNA replication forks, leading to the accumulation of mutations and chromosomal instability (3, 4).

In response to high baseline levels of replication stress, cancer cells rely on checkpoint kinase signaling modules, which play a crucial role in cell survival by allowing time for stress resolution and genome maintenance (2). Molecularly, this signaling pathway becomes activated by extended single-stranded DNAs (ssDNAs) due to various mechanisms, such as uncoupling of DNA unwinding from strand synthesis, formation of R loops at regions of transcription-replication conflicts, generation of ssDNA gaps as a result of fork bypass or resection of DNA lesions, etc. (4, 5). The accumulated ssDNAs are avidly bound by replication protein A (RPA) and can undergo fork reversal to avoid fork collapse (6). RPA recruits the protein kinase ataxia telangiectasia and Rad3-related protein (ATR) via ATR-interacting protein (ATRIP). ATR subsequently phosphorylates a multitude of targets, including checkpoint kinase 1 (CHK1) (3). An important CHK1 target is WEE1, whose phosphorylation causes inhibition of cyclin-dependent kinase (CDK1/CDK2) activity (7). To this end, activation of this pathway is critical to cell-cycle arrest, fork stabilization, origin-firing suppression, and so on. This, in turn, promotes fork repair and restart to complete replication at stress-affected loci (5).

Mounting evidence in cancer cells has revealed that elevated replication stress and dependency on checkpoint kinases can be leveraged as a vulnerability for drug targeting (8–10). The triggering of suprathreshold replication stress by simultaneous inhibition of nucleotide synthesis and checkpoint kinases is an appealing approach to elicit replication catastrophe (11, 12). Many checkpoint kinase inhibitors have been developed and are under clinical evaluation in combination with chemotherapy or radiotherapy (2). However, highly proliferative tissues can also be susceptible to this damage, and several trials have failed due to intolerable side effects such as cardiotoxicity (10). Exacerbation of replication stress selectively in cancers by cotargeting different checkpoint kinases (13–15) highlights the potential for combinations with drugs that provoke replication stress through the same pathway. However, it remains unknown whether compounds that target different aspects of replication stress through an integrative network can work synergistically with checkpoint kinase inhibitors to specifically induce replication catastrophe in cancer cells.

In addition to DNA replication, the chromatin structure experiences global disturbances during genome duplication. Downstream from replication forks, nucleosomes are reassembled, epigenetic marks on DNA are reestablished, and sister chromatids are held together by cohesin complexes to prevent their segregation before mitosis (16, 17). A growing body of evidence indicates that perturbation of these processes by inhibiting key factors, such as histone chaperons, acetyltransferases/deacetylases, and methyltransferases/demethylases, often results in aberrant chromatin organization, dysregulation of gene expression, replication fork stalling, and genome instability (17–19). Moreover, interruption of histone supply or posttranslational modifications have also been shown to increase sensitivity to checkpoint kinase inhibitors (20–23). Therefore, epigenetic modifiers could be attractive pharmacologic targets to elicit replication stress and synergize with checkpoint kinase inhibitors for cancer treatment.

Here, we performed an epigenetic compound screen to uncover synthetically lethal interactions with replication checkpoint kinases as an unexplored vulnerability in cancer cells. We identify histone deacetylase 8 (HDAC8) as a promising druggable candidate whose inhibition gave rise to extensive replication stress, robust DNA damage, and persistent S-phase arrest when combined with checkpoint kinase inhibitors. The synthetic lethality of the dual inhibition was validated in various in vitro and in vivo models, including patient tumor–derived xenograft (PDX) and organoid (PDO) models, indicating its potential for clinical applications. Moreover, this synergistic vulnerability is specific to cancer cells, suggesting a strong therapeutic index. We also showed that HDAC8 inactivation led to hyperacetylation of the cohesin subunit structural maintenance of chromosomes protein 3 (SMC3) on unreplicated chromatin and exacerbated the formation of DNA-RNA hybrids. These data reveal a critical function of HDAC8 in the regulation of genome stability during chromatin replication, and the cancer-specific synthetic lethality by inhibiting HDAC8 and checkpoint kinases supports a promising strategy for replication stress–targeting cancer therapy.

## Results

### Coinhibition of HDAC8 and replication checkpoints elicits severe replication stress, culminating in replication-dependent DNA double-stranded breaks.

To search for compounds that could work in tandem with checkpoint kinase inhibitors to disrupt replication fork stability, we performed rigorous high-throughput compound screening (Supplemental information and Supplemental Figure 1; supplemental material available online with this article; https://doi.org/10.1172/JCI165448DS1). This involved evaluation of the cellular DNA damage response using H2AX phosphorylation at Ser139 (γH2AX) as a readout and measuring ssDNA accumulation at replication forks by determining RPA protein loading on replicating chromatin marked with proliferating cell nuclear antigen (PCNA) after compound treatment. This screen led to the discovery of HDAC8 inhibitors that potentially induce replication stress and enhance genotoxicity from checkpoint kinase inhibition (Supplemental Methods and Supplemental Figure 1).

**Figure 1:**
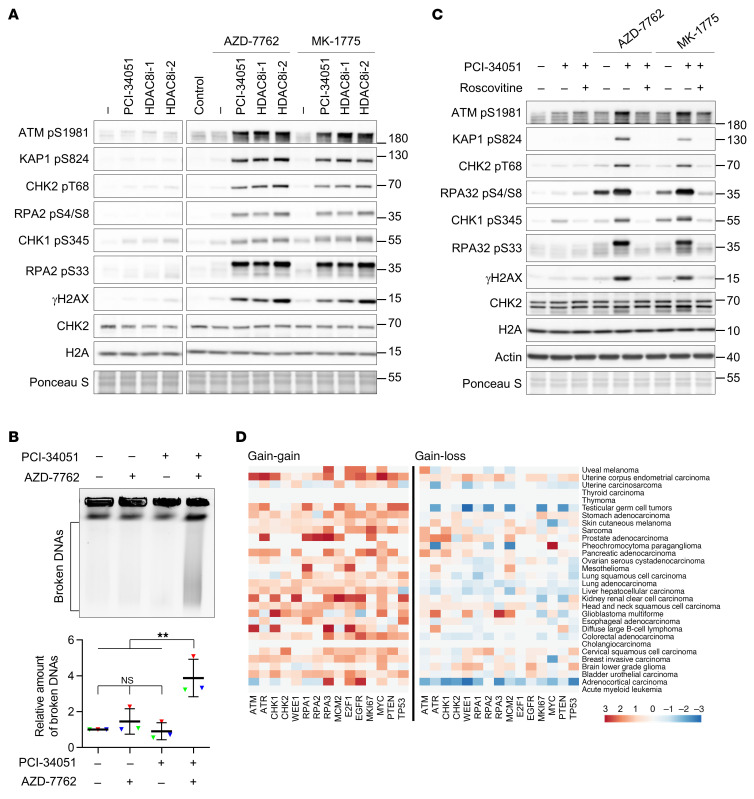
Coinhibition of HDAC8 and replication checkpoints elicits severe replication stress, culminating in replication-dependent DNA double-stranded breaks. (**A**) Western blot analysis of the DNA damage response in U-2 OS cells treated with the indicated compounds for 4 hours. Representative results from 1 of 2 biological replicates are shown. The lanes were run on the same gel but were noncontiguous. (**B**) PFGE analysis of DNA breaks in U-2 OS cells treated with the indicated compounds for 15 hours. Relative intensities of broken DNAs were obtained by normalizing individual values to the corresponding untreated control group values. Representative results (upper) and quantification of broken DNAs from 3 biological replicates (lower; *n* = 3) are shown. Triangles represent the relative intensities of each biological replicate; lines indicate the mean ± SDs of the biological replicates. **P < 0.01, by 1-way ANOVA. (**C**) Western blot analysis of the DNA damage response in U-2 OS cells treated with the indicated compounds in the presence or absence of roscovitine for 4 hours. Representative results from 1 of 2 biological replicates are shown. HDAC8 inhibitor: 40 μM; AZD-7762: 50 nM; MK-1775: 300 nM; roscovitine: 50 μM. (**D**) Heatmap showing the co-occurrence of either gain or loss of copy numbers of the indicated genes when the copy number of the HDAC8 gene was gained in individual TCGA cohorts.

In light of the high levels of RPA and γH2AX on replicating chromatin observed from our screen results, we sought to determine whether dual inhibition of HDAC8 and checkpoint kinases induces a lethal push of replication stress that triggers fork collapse, resulting in DNA double-stranded breaks (DSBs). As single regimens, treatment with either HDAC8 inhibitors or AZD-7762 induced a mild replication stress response and ATR activation, as evidenced by phosphorylation of CHK1 at S345 (pS345) and RPA2 at S33 (pS33) (Figure 1A). Strikingly, the combination of HDAC8 inhibitor and AZD-7762 induced a strong DSB signal, reflected by phosphorylation of ATM (pS1981), KAP1 (pS824), CHK2 (pT68), and RPA2 (pS4/S8) (Figure 1A). The accumulation of DSBs in cotreated cells was further validated by pulsed-field gel electrophoresis (PFGE) (Figure 1B). This synergistic genotoxicity was also observed in cells treated with combinations of AZD-7762 and other top hits identified from the screen (resminostat, scriptaid, SP2509, and ITF 2357) (Supplemental Figure 2A). In addition, gene-specific knockdown of HDAC8 by an siRNA showed the enhanced DNA damage response from checkpoint inhibition (Supplemental Figure 2B), further corroborating the pharmacological effect of HDAC8 inhibitors.

We also tested this combination model with other inhibitors targeting various checkpoint kinases, including WEE1 (MK-1775), CHK1 (UCN-01, prexasertib, and rabusertib), ATR (VE-821 and AZ-20), ATM (AZD-1390), and CHK2 (BML-277). We observed that coinactivation of HDAC8 with the WEE1 or ATR/CHK1 pathway, but not the ATM/CHK2 pathway, synergistically induced DNA damage (Supplemental Figure 2, C and D). These results indicate that HDAC8 inhibitors were more effective in combination with drugs targeting the S-phase checkpoint pathway. Given the function of checkpoints in antagonizing CDKs and origin firing, inactivation of CDKs by roscovitine or genetic depletion of the replication initiation factor CDC45 markedly reduced the DNA damage response from the dual inhibition (Figure 1C and Supplemental Figure 2, C and D), indicating that ongoing chromatin replication is a prerequisite for genome catastrophe caused by coinactivation of HDAC8 and checkpoint kinases. These data demonstrated that HDAC8 and checkpoint kinases cooperatively shielded replication forks in a replication-dependent manner.

Given our results showing that HDAC8 activity is crucial for maintaining replication fork stability in conjunction with checkpoint kinases, we hypothesized that HDAC8 is likely to be maintained and coamplified with components of the replication stress pathways in cancers. We analyzed the co-occurrence of gain of HDAC8 copy numbers with gain of copy numbers in genes involved in replication stress and oncogenic pathways for all cancer types included in The Cancer Genome Atlas (TCGA) pan-cancer initiative. We found that when tumors exhibited an increase in HDAC8 copy numbers, there was also a tendency toward elevated copy numbers of components involved in replication stress and carcinogenic pathways (Figure 1D). At the expression level, a positive correlation between HDAC8 and CHK1 or ATR was also found in pan-cancer datasets, suggesting coregulation between these genes in many tumor types (Supplemental Figure 2F). Furthermore, we examined the potential relationship between expression of HDAC8 and CHK1 with patient outcomes. High expression levels of both HDAC8 and CHK1 or ATR was linked to poor overall survival in a pan-cancer dataset from TCGA. (Supplemental Figure 2G). Collectively, these data suggest that HDAC8 may functionally interact with key regulators in genome maintenance pathways to sustain chromatin replication in cancer cells.

### Combined treatment with HDAC8 and checkpoint kinase inhibitors leads to synthetic vulnerability in various types of cancer cells.

**Figure 2:**
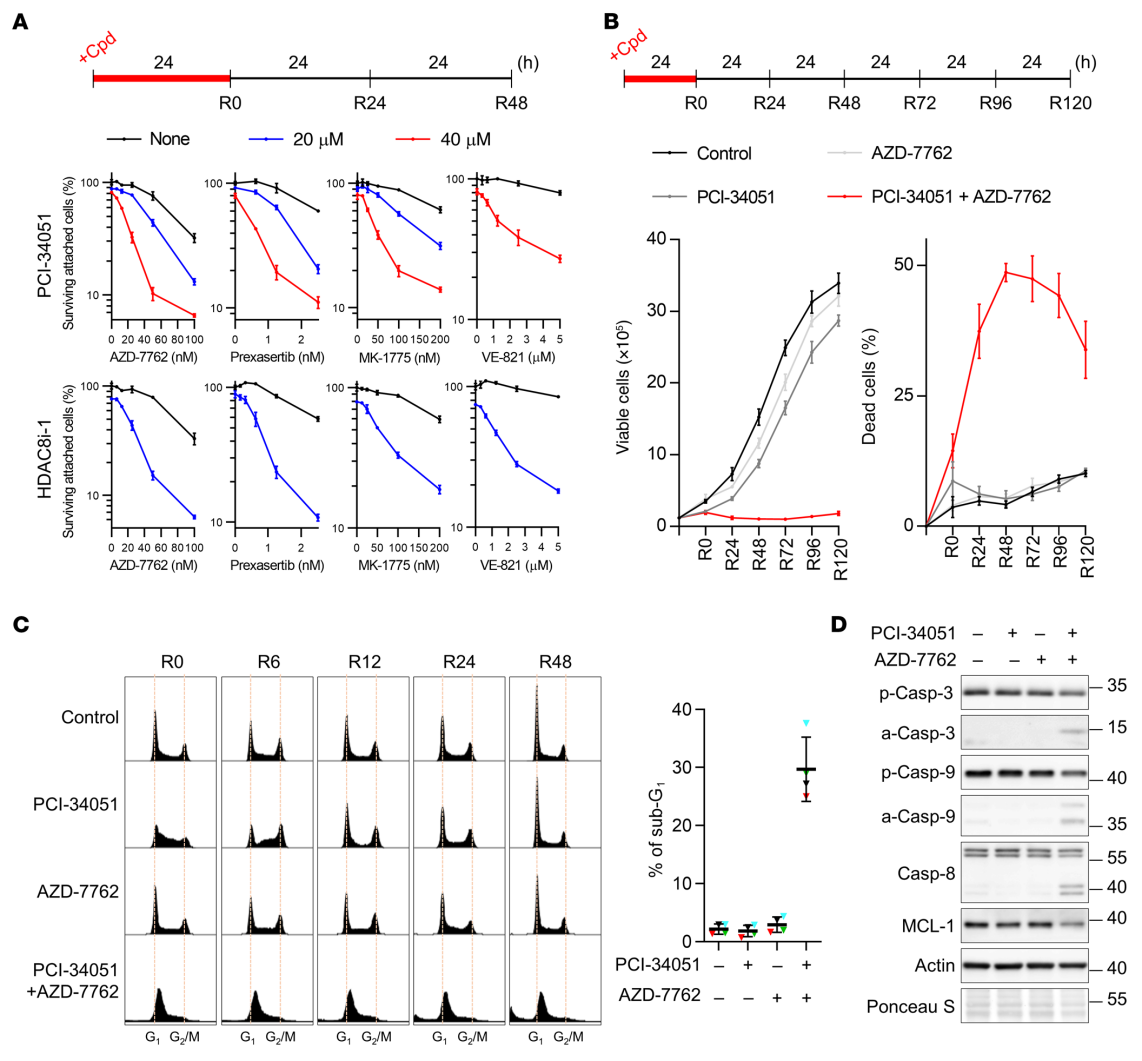
Combined treatment of HDAC8 and checkpoint kinase inhibitors leads to synthetic vulnerability. (**A**) Cytotoxicity analysis of the indicated treatments in U-2 OS cells. Experimental design and percentages of surviving attached cells at R48 from 1 of 3 biological replicates are shown with means and SDs (*n* =3). (**B**) Trypan blue exclusion assay of proliferation efficiency of U-2 OS cells treated with the indicated compounds. Experimental design and numbers of viable cells and percentages of dead cells from 2 biological replicates are shown with means and SDs (*n* = 6). (**C**) Cell-cycle analysis of U-2 OS cells treated as in **A**. Representative profiles and percentages of the sub-G_1_ population of the R48 samples from 4 biological replicates are shown (*n* = 4). Triangles represent the percentages of each biological replicate; lines indicate the mean ± SDs of the biological replicates. (**D**) Western blot analysis of apoptotic proteins in U-2 OS cells at R48. Data were collected from different sets of gel electrophoresis assays with equal loading of the same samples. Representative results from 1 of 4 biological replicates are shown. Cpd, compound; p-Casp, pro-caspase; a-Casp, active caspase. PCI-34051: 40 μM (**B**–**D**); AZD-7762: 50 nM (**B**–**D**).

Having demonstrated that coinhibition of HDAC8 and checkpoint kinases caused high levels of replication stress, we asked whether cotreatment would be sufficient to trigger cancer cell death. To mimic clinical settings, we treated U-2 OS cells with an HDAC8 inhibitor (PCI-34051 or HDAC8i-1), a checkpoint kinase inhibitor (AZD-7762, prexasertib, MK-1775, VE-821, rabusertib, AZ-20, AZD-1390, or BML-277), or combinations of one the HDAC8i and one of the checkpoint kinase inhibitors for 24 hours, and the survival fraction was analyzed 48 hours after release from treatment (Figure 2A). Consistent with the results of the genotoxicity analyses, we found that HDAC8 inhibition markedly potentiated the cell-killing effect of checkpoint kinase inhibitors against ATR, CHK1, or WEE1, but not ATM or CHK2 (Figure 2A and Supplemental Figure 3, A and B). Under our treatment conditions, the IC_50_ values for PCI-34051 and AZD-7762 in U-2 OS cells were approximately 80 μM and 90 nM, respectively (data not shown). Combining 40 μM PCI-34051 with 50 nM AZD-7762 killed more than 90% of the cells (Figure 2A). These findings indicate that these combinations not only increased treatment efficacy but also reduced the doses of both drugs needed to achieve nearly complete cell killing. Similar results were also obtained with various cancer cell lines, including triple-negative breast cancer (MDA-MB-231), colorectal cancer (CRC) (DLD-1 and HCT-116), cervical cancer (HeLa), and non–small cell lung carcinoma (H1299) cells (Supplemental Figure 3, B and D). Knockdown of HDAC8 in U-2OS cells further confirmed that the enhanced toxicity resulted from HDAC8 inactivation (Supplemental Figure 3E). Moreover, we observed that, in addition to HDAC8 inhibitors, treatments with other top hits of the screen (resminostat, scriptaid, SP2509, and ITF 2357) also enhanced AZD-7762 toxicity (Supplemental Figure 3F).

In line with the synergistic killing effect, we also found that the combination treatment for 24 hours almost completely blocked cell proliferation even after compound removal, and the percentage of dead cells increased dramatically over 48 hours (Figure 2B). By analyzing inhibitor-induced changes in cell-cycle progression, AZD-7762 treatment did not show obvious change in cell-cycle distribution (Figure 2C; 0 hours after the release from compound treatment [R0]). Although PCI-34051 induced accumulation in S phase (R0), subsequent drug washout allowed a return to normal cell-cycle progression (R6–R48). Remarkably, a large population of cotreated cells arrested in early S phase along with drug removal did not result in recovery of normal cell-cycle progression (Figure 2C). With time, the cells that received combination treatment showed signs of apoptosis, including an increase in the sub-G_1_ population, activation of caspases through cleavage, and a decrease in the antiapoptotic protein MCL1 (Figure 2, B–D). These data strongly suggest that simultaneous inhibition of HDAC8 and replication checkpoint kinases irreversibly leads to synthetic lethality.

### Genome collapse during chromatin replication causes the enhanced cytotoxicity of combination treatments.

**Figure 3:**
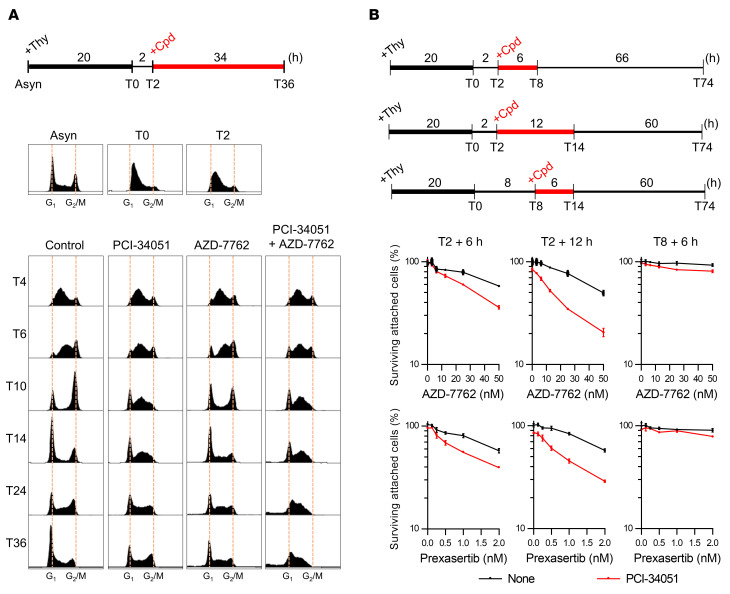
Cytotoxicity of combination treatments is the consequence of genome replication defects. (**A**) Cell-cycle analysis of thymidine-synchronized U-2 OS cells treated with the indicated compounds. Experimental design and representative results from 1 of 2 biological replicates are shown. (**B**) Cytotoxicity analysis of the indicated compounds in thymidine-synchronized U-2 OS cells. Experimental design and representative results from 1 of 2 biological replicates are shown. PCI-34051: 40 μM; AZD-7762: 50 nM. Thy, thymidine.

Combined evidence of extensive DNA damage in replicating cells and irreversible cell-cycle arrest in early S phase suggests that the numerous DNA lesions generated during chromatin replication block S-phase progression. To test this hypothesis, cells synchronized in early S phase using a single round of thymidine block were treated with the inhibitors, and cell-cycle progression was monitored in detail (Figure 3A). As expected, HDAC8 inactivation by PCI-34051 delayed S-phase progression (24, 25), whereas inhibition of CHK1/2 by AZD-7762 expedited the progression through S phase and probably mitotic exit when compared with the control (26) (Figure 3A). Critically, with the combination treatment, S-phase progression was completely stopped 4 hours after treatment initiation, and most cells accumulated in mid–S phase (Figure 3A). Only a small portion of cells, which were in G_2_/M at the time of drug administration, could pass through mitosis but arrested in early S phase of the following cell cycle (Figure 3A). Similar results were also obtained from G_1_ cells released from thymidine-nocodazole double synchronization, showing immediate cell-cycle arrest of dually treated cells when entering S phase, regardless of whether cells were exposed to drugs continuously or washed out (Supplemental Figure 4A).

The unaffected G_2_-M population suggests that the increased DNA damage response and cytotoxicity observed with coinhibition were specifically due to interference with genome replication during S phase, rather than with chromosome segregation during mitosis. To prove this, thymidine-synchronized cells enriched in early S phase (2 hours after the release from thymidine [T2]) or G_2_/M phase (T9) were pulsed with drugs for 5 hours, and the DNA damage responses were examined (Supplemental Figure 4B). Our data confirmed that DNA damage responses were elevated in replicating cells (T2+5h) as compared with the mitotic population (T9+5h) (Supplemental Figure 4B). Consistently, PCI-34051 enhanced the cytotoxicity of AZD-7762 or prexasertib when cells were treated in S phase (T2+6h and T2+12h; Figure 3B). In contrast, cells that were primarily in G_2_/M phase at the time of treatment were mostly unaffected by the combination treatment (T8+6h; Figure 3B), supporting our hypothesis that DNA replication is required for cytotoxicity. Altogether, these data reinforced our findings that coinhibition of HDAC8 and checkpoint kinases caused synergistic vulnerability during S phase by destabilizing replication forks in cancer cells.

### Synergistic cell killing by dual inhibition of HDAC8 and checkpoint kinase is selective for cancer cells.

**Figure 4:**
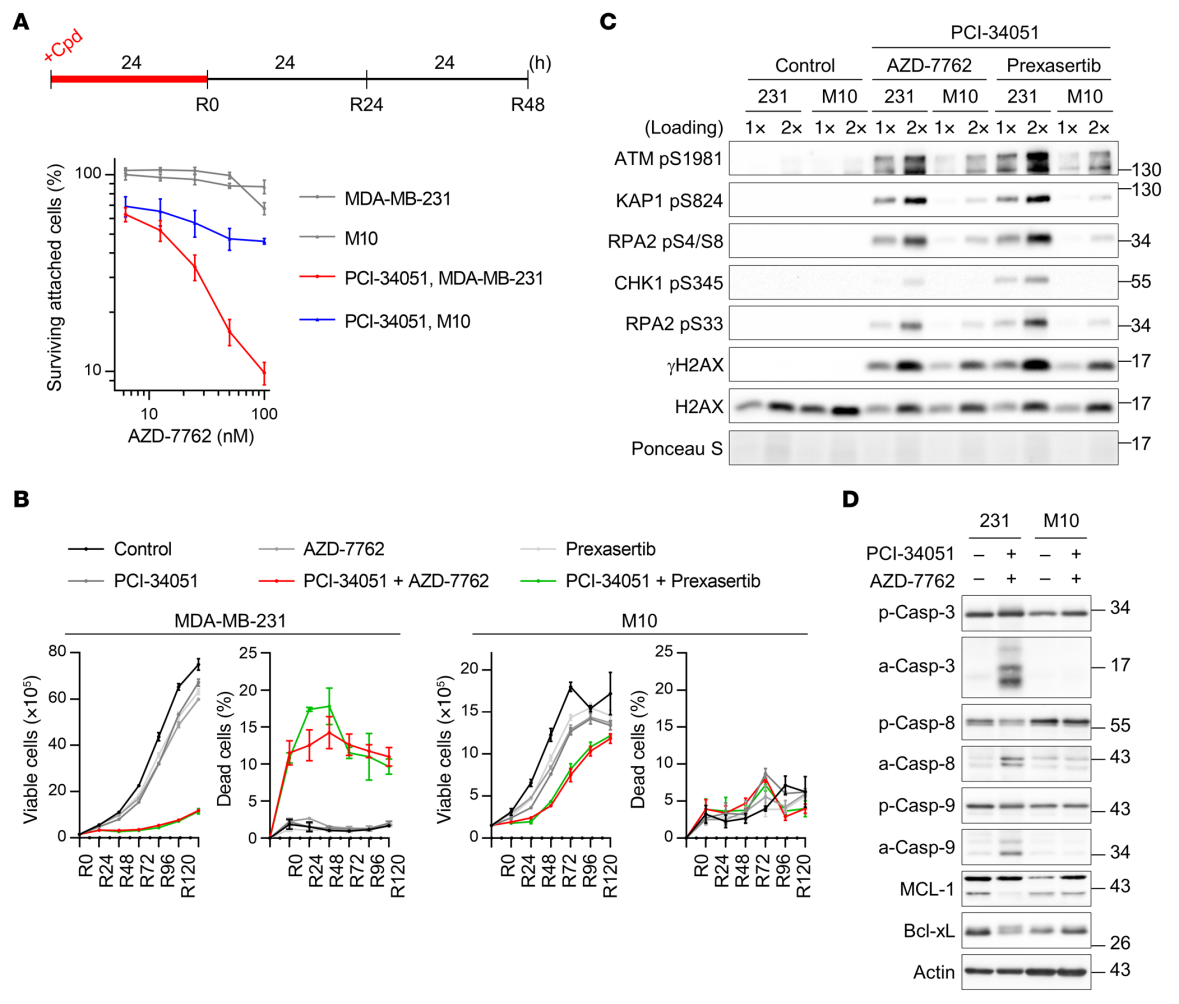
Synergistic cell killing by HDAC8 and checkpoint kinase inhibition is selective in cancer cells. (**A**) Cytotoxicity analysis of the indicated treatments in MDA-MB-231 and M10 cells. Experimental design and percentages of surviving attached cells at R48 from 2 biological replicates are displayed with means and SDs (*n* ≥5). (**B**) Trypan blue exclusion assay of proliferation efficiency of MDA-MB-231 and M10 cells treated with the indicated compounds. Numbers of viable cells and percentages of dead cells from 2 biological replicates are shown with means and SDs (*n* ≥3). (**C** and **D**) Western blot analysis of the DNA damage response at R0 (**C**) and apoptotic proteins at R48 (**D**) in MDA-MB-231 and M10 cells treated with the indicated compounds. Data were collected from different sets of gel electrophoresis assays with equal loading of the same samples (**D**). Representative results from 1 of 3 biological replicates are shown. PCI-34051: 80 μM (**A**, **C**, and **D**) or 40 μM (**B**); AZD-7762: 100 nM (**C** and **D**) or 80 nM (**B**); prexasertib: 3 nM.

Replication stress is a well-known hallmark of tumor cells that is rarely observed in even the most highly proliferative normal tissues (8). This cancer-specific characteristic can thus be exploited as a therapeutic approach. To test whether the enhanced cell-killing effect of cotreatment was specific to cancer cells, the normal breast epithelial cells H184B5F5/M10 (M10) and MCF10A were utilized in comparison with the breast cancer cell line MDA-MB-231. Our data revealed that the combination only resulted in a modest cytotoxic response in M10 and MCF10A cells, but a robust cytotoxic response in the MDA-MB-231 cells (Figure 4A). Although we observed slow growth in normal cells treated with both inhibitors, drug removal allowed proliferation to resume (Figure 4B and Supplemental Figure 5A). The dual treatment caused much greater genotoxicity in MDA-MB-231 cells than in M10 cells (Figure 4C). These observations suggest that proficient DNA repair can protect normal cells from catastrophic genome instability even after inhibition of HDAC8 and checkpoint kinases. Consistent with the hypothesis of ubiquitous fork collapse in cancer cells, cotreatment also resulted in apoptosis and an accelerated death rate in MDA-MB-231 but not M10 or MCF-10A cells (Figure 4, B and D, and Supplemental Figure 5A), further illustrating the cancer-selective sensitivity. In agreement with the observations of cell-cycle arrest of U-2 OS cells (Figure 2C), the combination treatment perpetually arrested the majority of MDA-MB-231 cancer cells in S phase. In contrast, only a slight increase of S-phase and sub-G_1_ populations were detected in the normal M10 cells under the same treatment (Supplemental Figure 5B). These data support the observation that the synergistic effects of cotreatment on replication dynamics are cancer cell specific and allude to the safe applicability of dual inhibition to treat a variety of cancers.

### Inactivation of HDAC8 perturbs replication elongation.

**Figure 5:**
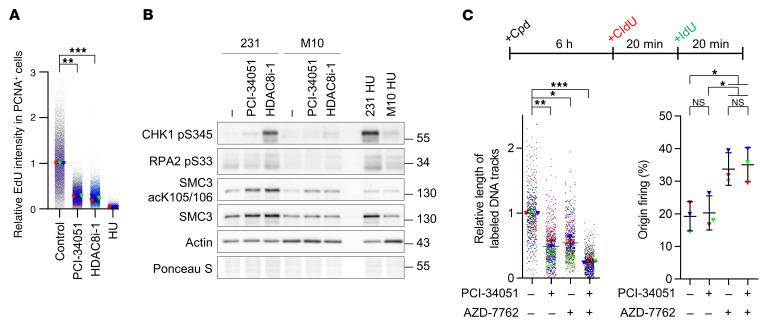
HDAC8 inactivation impairs replication elongation. (**A**) Immunofluorescence analysis of DNA replication efficiency in U-2 OS cells treated with the indicated HDAC8 inhibitors for 6 hours. Relative EdU intensities in PCNA^+^ replicating cells were obtained by normalizing individual values to the median of the corresponding untreated control group. Dots indicate normalized values of individual cells from each biological replicate labeled with the corresponding colors; triangles represent the median of each biological replicate; lines indicate the mean ± SDs of the medians from biological replicates. (**B**) Western blot analysis of the DNA damage response in MDA-MB-231 and M10 cells treated with the indicated compounds for 4 hours. Representative results from 1 of 2 biological replicates are shown. (**C**) DNA fiber analysis of the replication dynamics of MDA-MB-231 cells treated with the indicated compounds for 6 hours. Experimental design and quantitation results of total length of fibers and means ± SDs of the percentages of origin firing from 3 biological replicates are shown. PCI-34051: 40 μM (**A** and **B**) or 20 μM (**C**); HDAC8i-1: 40 μM; AZD-7762: 50 nM. HU: 1 mM. *P < 0.05, **P < 0.01, and ***P < 0.005, by 2-tailed, paired *t* test (**A** and **C**, left panel) and 1-way ANOVA (**C**, right panel).

A vast majority of reports have documented the involvement of HDAC8 in tumorigenesis at multifaceted levels to promote cancer cell survival, repress apoptosis, and prevent telomere shortening (27, 28). Inhibition of HDAC8 alone slowed S-phase progression and augmented γH2AX staining (Figure 2C, Figure 3A, and Supplemental Figure 6A) (24, 25), suggesting that HDAC8 itself plays a role in regulating genome stability during chromatin replication. To better understand the role of HDAC8 in chromatin replication, we evaluated DNA synthesis by measuring the incorporation efficiency of the thymidine analog 5-ethynyl-2′-deoxyuridine (EdU). Both PCI-34051 and HDAC8i-1 reduced EdU incorporation in proliferating cells (Figure 5A and Supplemental Figure 6B). We also observed similar effects on DNA synthesis and DNA damage induction in cells with siRNA-induced HDAC8 depletion (Supplemental Figure 6C). These results demonstrate that HDAC8 activity was required for genome replication. Similar to HDAC8 inhibitors, the 3 top hits of the screen, scriptaid, SP2509, and ITF 2357, also significantly impeded DNA replication (Supplemental Figure 6D). Interestingly, another top hit, resminostat, slightly augmented the overall nucleotide incorporation efficiency and the level of chromatin-bound PCNA (Supplemental Figure 6D). These findings show that our screening approach is reliable for discovering agents that disturb chromatin replication.

In addition, short-term HDAC8 inhibition (4 hours) induced replication stress demonstrated by increased levels of phosphorylated CHK1 and RPA2 (pS33), while longer drug exposure (24 hours) resulted in the activation of DSB signaling, as shown by phosphorylation of CHK2 and RPA2 (pS4/S8) (Supplemental Figure 6E), potentially due to collapse of a portion of replication forks with persistent stress. Notably, cancer cells were more susceptible to HDAC8 inhibition, as both HDAC8 inhibitors induced greater checkpoint activation in MDA-MB-231 cells compared with normal M10 cells (Figure 5B).

To decipher how HDAC8 inhibition perturbs replication dynamics, we performed single-molecule DNA fiber analysis after drug treatment for 6 hours (Figure 5C). As expected, CHK1/2 inhibition by AZD-7762 increased origin density and compromised replication elongation (Figure 5C and Supplemental Figure 6F) (29, 30). HDAC8 inhibition by PCI-34051 also significantly slowed replication speed but left the occupancy of origin firing unaffected (Figure 5C), indicating that HDAC8 protected replication elongation. Consistent with this observation, HDAC8 inhibitor treatment alone did not affect loading of the initiation factor CDC45 on chromatin (Supplemental Figure 6G). Interestingly, in the case of double inhibition, replication elongation was severely impaired, as revealed by the prevalence of small fragments of DNA fibers (Figure 5C and Supplemental Figure 6F). This observation is consistent with a genome-wide induction of replication stress and thus synthetic vulnerability. Together, these studies imply that HDAC8 alone serves as a mediator of replication surveillance which works with checkpoint kinases to prevent replication catastrophe.

### HDAC8 inhibition abrogates deacetylation of SMC3 throughout the cell cycle.

**Figure 6:**
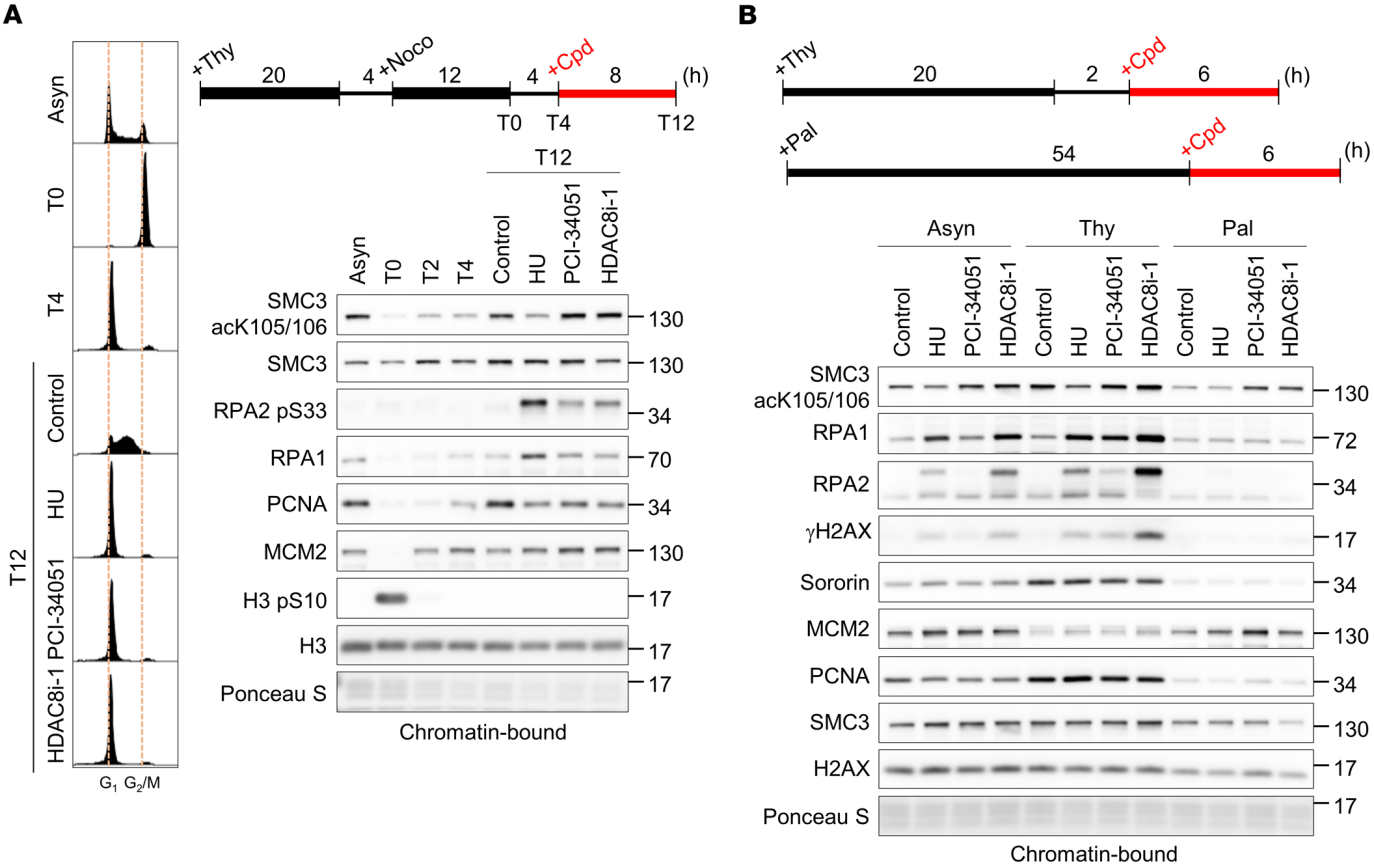
HDAC8 inhibition abrogates SMC3 deacetylation throughout the cell cycle. (**A**) Western blot analysis of SMC3 acetylation and replication factors loading on chromatin in U-2 OS cells that were thymidine and nocodazole (Noco) synchronized and treated with the indicated compounds. Experimental design, representative cell-cycle profiles, and Western blot results are shown. (**B**) Western blot analysis of SMC3 acetylation and replication factors loading on chromatin in thymidine- or palbociclib-synchronized U-2 OS cells treated with the indicated compounds. Experimental design and representative results from 1 of 2 biological replicates are shown. HU: 1 mM; HDAC8 inhibitors: 40 μM; palbociclib (Pal): 4 μM; AZD-7762: 50 nM. Asyn, asynchronized.

Our findings described above illustrate the contribution of HDAC8 to safeguarding the integrity of the replicating genome. However, it remains unclear how HDAC8 functions to prevent replication stress. HDAC8 has demonstrated in vitro deacetylase activity for histone proteins (27), but this activity has yet to be validated in vivo. In addition, our previous study did not find HDAC8 enriched at replication forks or nascent chromatin (31), suggesting that HDAC8 may interact with proteins localized on or nearby the parental/mature chromatin. SMC3, a subunit of the cohesin complex, is a widely recognized HDAC8 substrate involved in entrapment of sister chromatids and genome maintenance, of which dysregulated acetylation has been linked to Cornelia de Lange syndrome, a genetic disorder that affects many organs (32, 33). The gradual acetylation of the head domain of SMC3 during S phase has been shown to be important for cohesin establishment on sister chromatids in humans. During mitosis, SMC3 is released from chromosomes, and the rapid removal of acetyl groups by HDAC8 allows SMC3 recycling for the next cell cycle (34–36). Given that HDAC8 inactivation undoubtedly exerts its effect at S phase, we hypothesized that, while the acetyl group on SMC3 is removed during mitosis to allow chromosome segregation, the turnover of acetylation might also play a crucial role in cohesin dynamics during other phases of the cell cycle. To test this, we first confirmed that SMC3 acetylation was almost fully erased in mitotic cells prepared by thymidine/nocodazole double synchronization (Figure 6A, T0). As cells exited mitosis and entered the next G_1_ and S phases, acetylated SMC3 progressively accumulated and reached the highest level in S phase (T2–T12). To understand how HDAC8 inhibition disrupts the cell-cycle–dependent dynamics of SMC3 acetylation, we treated cells that were just about to enter S phase (T4) with an HDAC8 inhibitor. As a comparison, suppression of DNA synthesis by HU kept cells in the G_1_/early-S transition and thus prevented augmentation of SMC3 acetylation (T12). Although cells with HDAC8 inhibition also accumulated in G_1_/early S phase, SMC3 acetylation increased to a level comparable to that of untreated cells in mid–S phase (T12). These results indicate that, aside from its role during mitosis, HDAC8 also removed acetyl groups from SMC3 during S phase in a manner uncoupled from fork progression. Similarly, we also found that HDAC8 actively deacetylated SMC3 in G_1_ as HDAC8 inactivation elevated SMC3 acetylation levels in cells synchronized by the CDK4/6 inhibitor palbociclib (Figure 6B and Supplemental Figure 7A). In addition, depletion of ESCO1, the main SMC3 acetyltransferase in G_1_, prevented the upregulation of SMC3 acetylation by HDAC8 inhibitor treatment (Supplemental Figure 7B). These results indicate that HDAC8 was active throughout the cell cycle to maintain a low level of SMC3 acetylation on chromatin.

### HDAC8 inhibition leads to R-loop accumulation.

**Figure 7:**
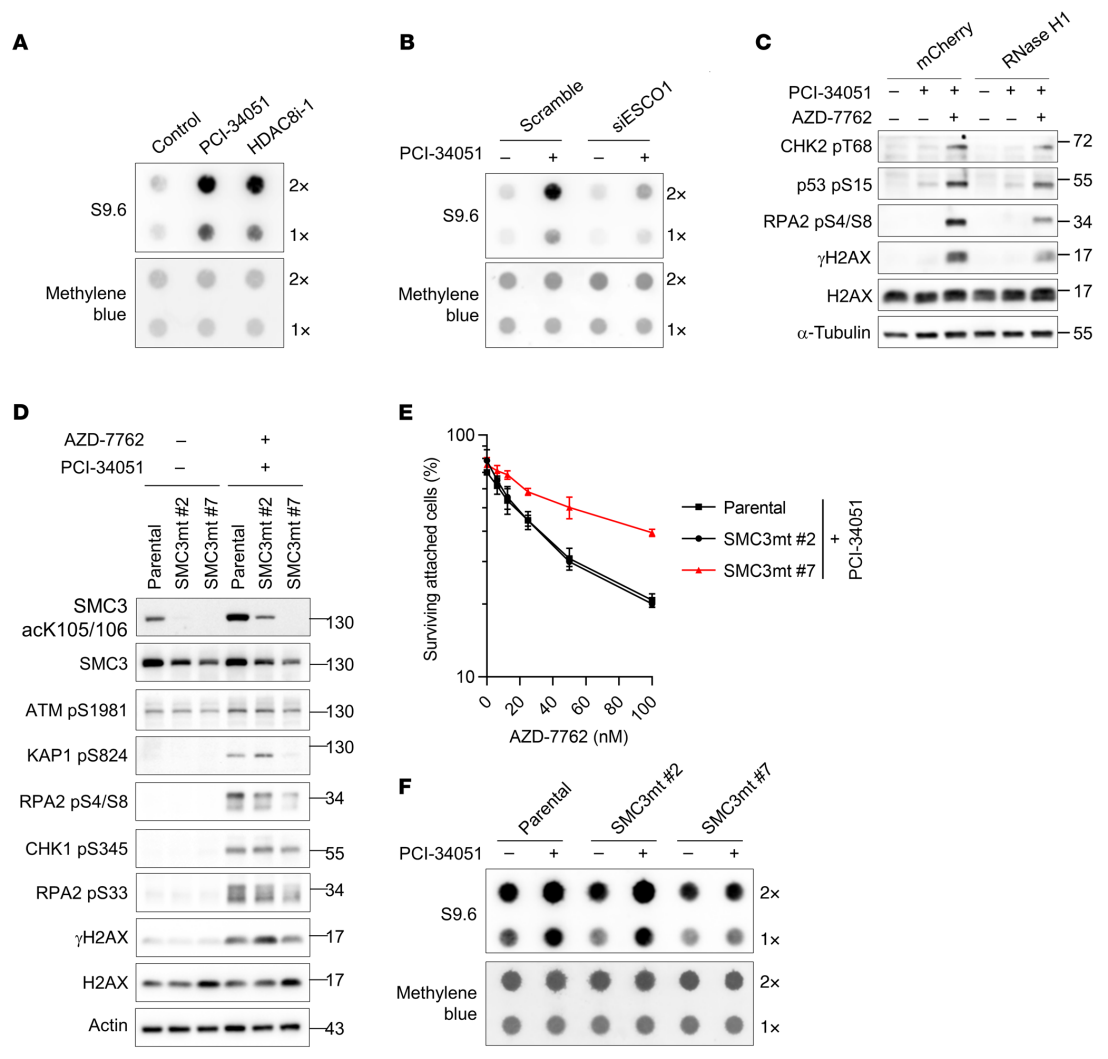
HDAC8 inhibition leads to R-loop accumulation. (**A** and **B**) Dot blot analysis of DNA-RNA hybrids in normal (**A**) or ESCO1-depleted (**B**) U-2 OS cells treated with the indicated compounds for 24 hours. DNA-RNA hybrids were probed with the S9.6 antibody. (**C** and **D**) Western blot analysis of the DNA damage response in RNase H1-expressing U-2 OS cells (**C**) or SMC3-mutant–expressing MDA-MB-231 cells (**D**) treated with the indicated compounds for 4 hours. (**E**) Cytotoxicity analysis of the indicated treatments in SMC3-mutant–expressing MDA-MB-231 cells. Percentages of surviving attached cells are displayed with means and SDs (*n* = 3). (**F**) Dot blot analysis of DNA-RNA hybrids in SMC3- mutant–expressing MDA-MB-231 cells treated with the indicated compounds for 24 hours. Representative results from 1 of 2 biological replicates are shown (**A**–**F**). HDAC8 inhibitors: 40 μM; AZD-7762: 50 nM.

Well-coordinated cohesin dynamics is critical for the proper topological organization of genome structure and transcriptional regulation (37). A recent study found that cohesin complexes bind to R-loops, a genome structure composed of a DNA-RNA hybrid and a displaced ssDNA (38). Given that R-loops have been well accepted as an obstacle to chromatin replication (39), we directly investigated the effect of HDAC8 inhibition on R-loop formation using the S9.6 antibody that specifically recognizes the structure of DNA-RNA hybrids present in the purified nucleic acids (40). By performing dot blot analyses, we detected a dramatic increase in the level of DNA-RNA hybrids in cells with HDAC8 inhibition or depletion (Figure 7A and Supplemental Figure 8, A and B). The observed accumulation of R-loops resulting from HDAC8 inhibition also correlated with aberrant SMC3 hyperacetylation, as this phenomenon was reduced in cells with establishment of sister chromatid cohesion *N*-acetyltransferase 1 (ESCO1) knockdown (Figure 7B). Moreover, the expression of RNase H1 antagonized the emergence of R-loops upon HDAC8 inhibitor treatment and partially repressed the DNA damage response activated by cotreatment (Figure 7C and Supplemental Figure 8C). This implies the existence of a mechanism by which HDAC8 inhibition induces replication stress through promotion of R-loop accumulation that is likely due to impaired cohesion dynamics.

To further link SMC3 hyperacetylation to replication stress induced by HDAC8 inhibition, we exploited CRISPR genome-editing technology to generate cells expressing the SMC3 mutant. As expected, we were unable to obtain cells with SMC3 knockout, which supports the notion that SMC3 is essential for cohesion establishment and cell survival (41). We observed that SMC3 expression in some MDA-MB-231 clones was decreased (represented by SMC3mt nos. 2, 7, and 21), and some of which demonstrated a lack of SMC3 acetylation even in response to PCI-34051 treatment (nos. 7 and 21) (Figure 7D and Supplemental Figure 8D). Compared with parental cells and mutant cells retaining SMC3 acetylation (no. 2), cells deficient in SMC3 acetylation (nos. 7 and 21) were more resistant to the combinational treatment in terms of DNA damage and cytotoxicity (Figure 7, D and E, and Supplemental Figure 8D). Consistently, we observed that DLD-1 and PANC-1 cells deficient in SMC3 acetylation were also less responsive to the combined treatment (Supplemental Figure 8, E and F). Additionally, HDAC8 inhibition failed to trigger R-loop accumulation in cells lacking SMC3 acetylation (Figure 7F). Treating SMC3-mutant cells with either an HDAC8 inhibitor or a checkpoint kinase inhibitor frequently induced less DNA damage when compared with parental cells (Supplemental Figure 8, D–F), supporting the hypothesis that HDAC8-mediated SMC3 deacetylation plays a crucial role in reducing replication stress. The absence of HDAC8 activity resulted in SMC3 hyperacetylation, which increased replication stress and, consequently, enhanced sensitivity to checkpoint kinase inhibitors. Collectively, our findings strongly suggest that HDAC8 activity was necessary for the coordinated reversal of SMC3 acetylation and R-loop resolution during genome replication. Note that expression of the acetylation-deficient SMC3 mutant only partially rescued the phenotypes caused by HDAC8 inhibition, implying that other mechanisms may also contribute to HDAC8 inhibitor–induced replication stress.

### Cotreatment with an HDAC8 inhibitor and AZD-7762 suppresses tumor growth in human CRC organoid and mouse xenograft models.

Encouraged by the findings that combined inhibition of HDAC8 and checkpoint kinases generated synergistic toxicity in multiple cancer cells, we then assessed the therapeutic value of this combination in vivo. However, our initial attempts with dual inhibition by PCI-34051 and AZD-7762 in mice inoculated with DLD-1 xenografts did not show a detectable difference compared with just AZD-7762 treatment (data not shown). In addition, PCI-34051 treatment alone appeared to be ineffective at reducing tumor volumes (data not shown). PCI-34051 is a conventional hydroxamic acid–based HDAC8 inhibitor that is widely used in vitro with high potency (27, 28). However, previous studies showed that hydroxamic acid–based inhibitors are extensively glucuronidated in vivo to inactive metabolites as a major mode of metabolism, thereby displaying undesirable metabolic and pharmacokinetic profiles (42, 43). Since HDAC8i-3 described in the literature for in vivo use also belongs to the class of hydroxamate inhibitors (44, 45), we aimed to determine whether glucuronidation affects its in vivo stability as well as antitumor effects. To evaluate the glucuronidation of PCI-34051 and HDAC8i-3, we treated nude mice with 80 mg/kg of each compound, and plasma was collected after 15 minutes for detection of the free compound and its glucuronide using liquid chromatography–tandem mass spectrometry (LC-MS/MS) analysis (Supplemental Figure 9, A and B). By chromatography, in addition to the expected peak of the free compound (PCI-34051: 3.67 min; HDAC8i-3: 3.94 min; Supplemental Figure 9, C and D), we detected an additional peak corresponding to the glucuronidated metabolites (PCI-34051: 3.55 min; HDAC8i-3: 3.72 min; Supplemental Figure 9, C and D). When we compared free PCI-34051 and HDAC8i-3, there was substantially more HDAC8i-3 circulating in the mouse plasma 15 minutes after the drugs were administered (Supplemental Figure 9, C and D), indicating better pharmacokinetic properties. We thus conclude that the HDAC8i-3 is more stable in mouse plasma.

HDAC8i-3 exhibited potent inhibitory activity and high selectivity toward HDAC8 in vitro (Supplemental Figure 10A) (44) and in cells (Supplemental Figure 1H). HDAC8i-3 treatment recapitulated the responses induced by PCI-34051, including impaired DNA replication (Supplemental Figure 10B), checkpoint kinase activation (Supplemental Figure 1H), and RPA hyperloading and phosphorylation on chromatin (Supplemental Figure 1F and 10C). These effects were more pronounced than what we observed with PCI-34051 treatment. Moreover, HDAC8i-3 treatment increased the level of R-loops in both U-2 OS and MDA-MB-231 cells and enhanced DNA damage induced by AZD-7762 treatment (Supplemental Figure 10, D–F). These results strongly indicate that HDAC8i-3 is a specific HDAC8 inhibitor and more potent than PCI-34051.

**Figure 8:**
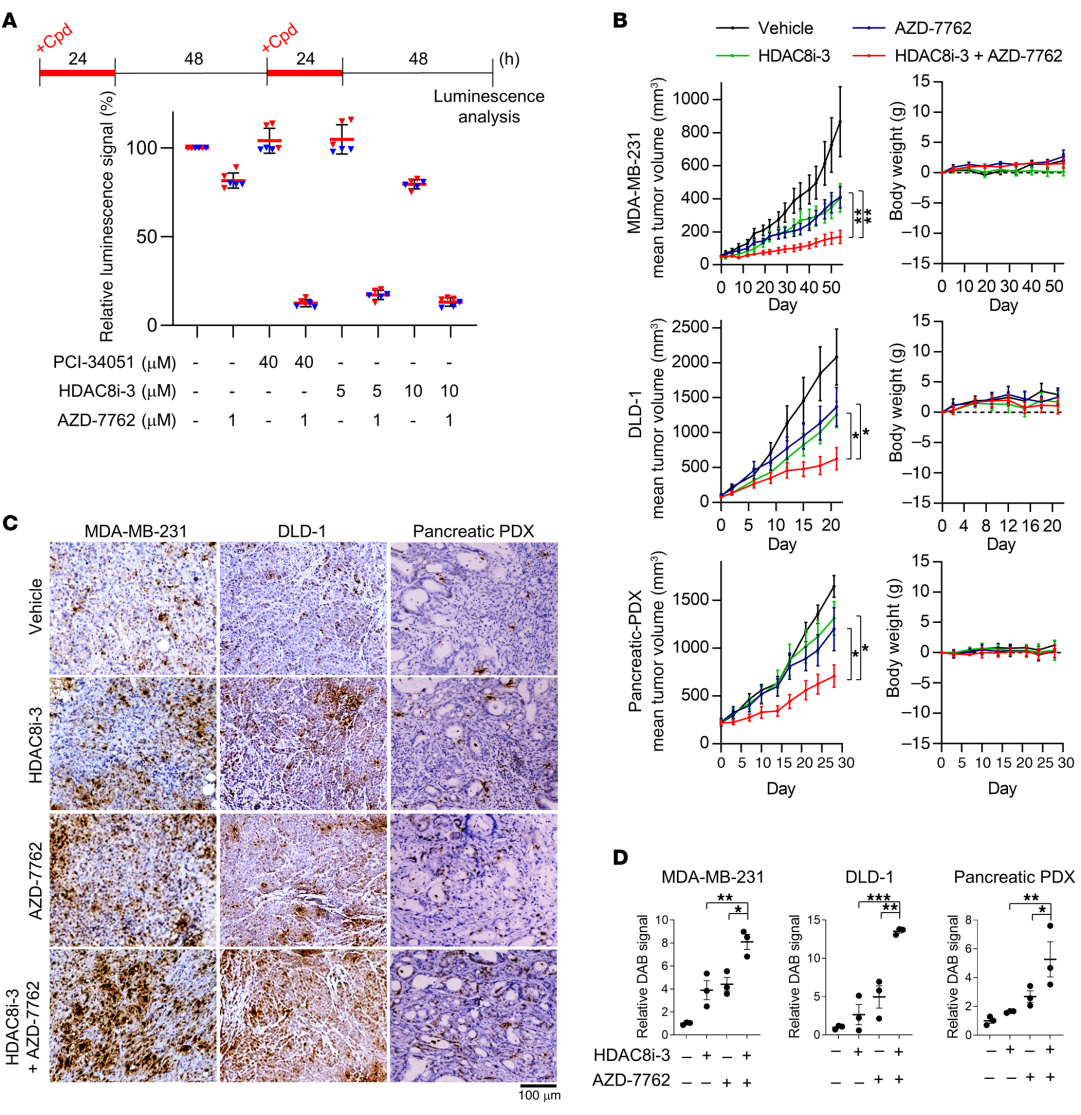
Coinactivation of HDAC8 and checkpoint kinase suppresses tumor growth in human cancer organoids and mouse xenograft models. (**A**) Cytotoxicity analysis of CRC PDOs. Experimental design and percentages of surviving PDOs at the endpoint from 2 biological replicates are shown. Relative luminescence signals were obtained by normalizing individual values to the mean of corresponding untreated control group. Triangles represent the technical repeats of each biological replicate; lines indicate the mean ± SDs of all replicates (*n* = 6). (**B**) Tumor growth analysis of athymic mice bearing MDA-MB-231 (*n* ≥9), DLD-1 (*n* ≥6), or patient pancreatic (*n* ≥9) established tumors. Mice were intraperitoneally injected with vehicle, 10 mg/kg AZD-7762, and/or 50 mg/kg HDAC8i-3 (once a day for MDA-MB-231; twice a day for DLD-1 and pancreatic PDX), 5 times per week. Tumor volumes and mouse body weight changes were monitored throughout the treatment schedules. (**C** and **D**) IHC analysis of the DNA damage response by γH2AX staining (DAB, brown) of tumors (*n* = 3) excised from MDA-MB-231, DLD-1, and pancreatic PDX xenografts 1 hour after the last injection. Representative images (**C**) are shown, and quantification results (**D**) are expressed as the mean ± SEM. *P < 0.05, **P < 0.01, and ***P < 0.001, by 1-way ANOVA.

We therefore evaluated whether HDAC8i-3 is synergistic with AZD-7762 by performing cytotoxicity assays and calculating the combination index (CI) using the Chou-Talalay method (46). We observed strong synergy with HDAC8i-3 and CHK1/2 inhibitors in all tested cancer cell lines (CI <0.6) (Supplemental Figure 10G). Moreover, we assessed the efficacy of the combinations using a cancer organoid system, which has emerged as a strong predictor of clinical efficacy. Human CRC PDOs were cultured for 3 days and then treated with an HDAC8 inhibitor (PCI-34051 or HDAC8i-3), AZD-7762, or combined for 2 rounds. Our findings demonstrated a notable suppression of PDO growth with the combinational treatment, while either single treatment only showed a modest effect (Figure 8A and Supplemental Figure 11A).

To test whether HDAC8i-3 potentiates the tumor killing effect of AZD-7762, we subcutaneously inoculated MDA-MB-231 breast cancer and DLD-1 colon cancer cells into athymic mice. Mice were randomized to receive either vehicle, HDAC8i-3, AZD-7762, or both, 5 times a week. The combination treatment remarkably delayed tumor progression compared with either monotherapy alone, demonstrating a strong suppression of tumor growth (Figure 8B). Furthermore, we also evaluated the treatments in a pancreatic PDX model (Figure 8B). The therapeutic effect of cotreatment was significantly greater than HDAC8i-3 or AZD-7762 treatment in all 3 tumor types (Figure 8B). To analyze whether the cotreatment induced DNA damage, tumors were extracted 1 hour after the last treatment, and γH2AX was assessed by IHC. Consistent with the in vitro experiments, we observed considerably more pan-nuclear γ*H2*AX staining in tumors with the combination (Figure 8, C and D), suggesting an induction of apoptosis (47).

As predicted by the minimal cytotoxicity observed in normal cells after cotreatment in vitro, mice from all 3 xenograft groups (MDA-MB-231, DLD-1, and pancreatic PDX) showed no measurable changes in body weight (<3%) throughout the length of their treatment (Figure 8B). Mice in all treatment groups remained equally active and displayed no diarrhea or abnormal food intake throughout the treatment. To evaluate drug-induced damage in normal tissues, small intestine, kidney, liver, and lungs were collected from the tumor-bearing mice (*n* = 3 per group) after the completion of drug treatment. Except for ileum, no other normal tissues showed any DNA damage, as revealed by γH2AX staining after treatment (Supplemental Figure 11B). Although we observed increased γH2AX staining in the crypt region of some small intestines, the length of villi was not reduced after either single or combination treatment when compared with villi lengths in controls (Supplemental Figure 11C). Taken together, these results strengthen our concept that cotargeting HDAC8 and checkpoint kinases could be an efficacious and well-tolerated strategy for cancer therapy. The results obtained from both PDO and PDX models further underscore the potential of the combined therapy for clinical use.

## Discussion

**Figure 9:**
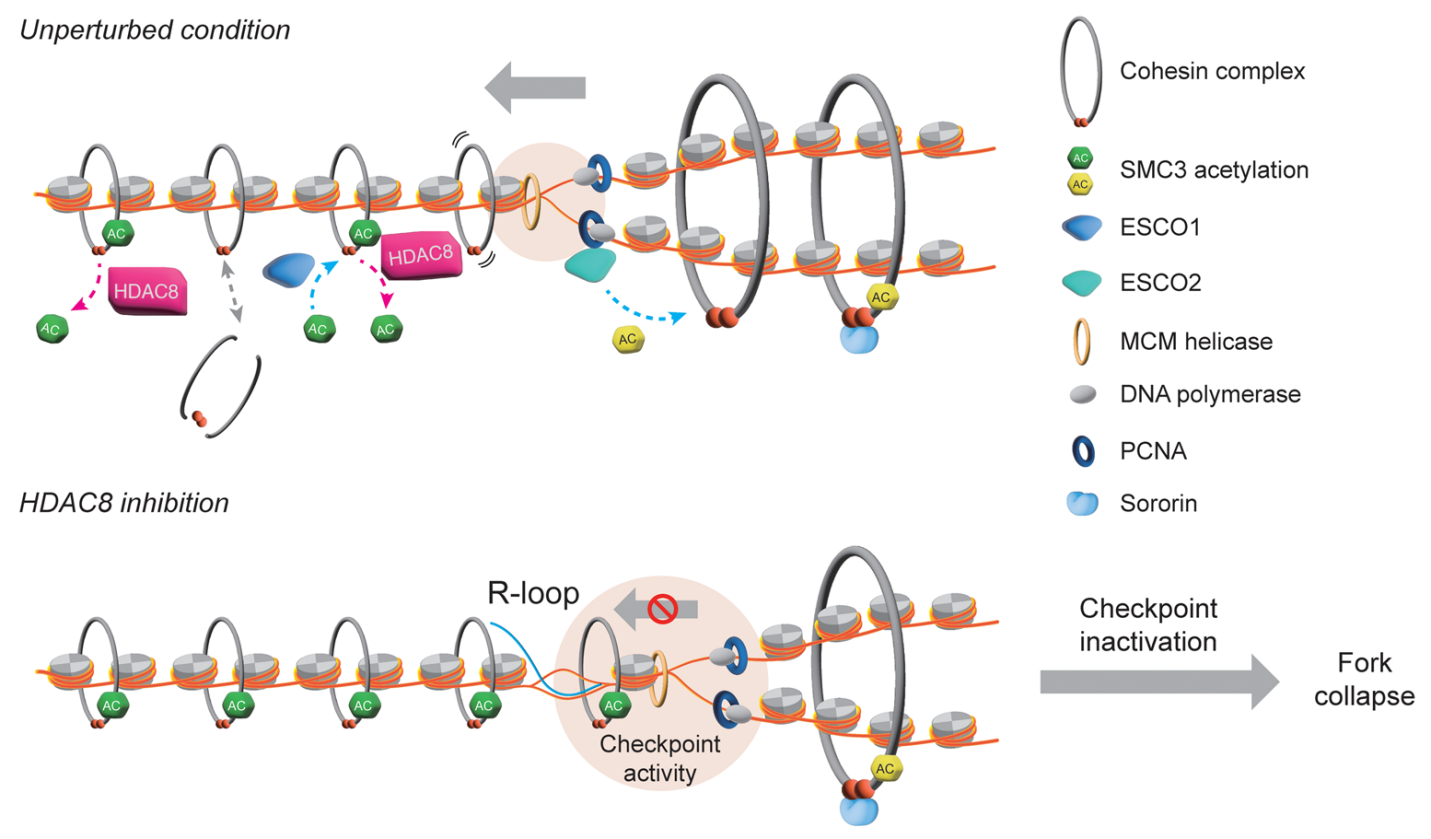
Proposed model of HDAC8 functions in chromatin replication. In the unperturbed condition, cohesin complexes are loaded on chromatin before DNA replication. The SMC3 acetyltransferase ESCO1 and the deacetylase HDAC8 coordinately regulate the turnover of SMC3 acetylation to control cohesin mobility and genome organization. SMC3 deacetylation by HDAC8 increases cohesin flexibility on chromatin and thus facilitates the fork passing through the cohesin complex. After chromatin replication, ESCO2 acetylates SMC3 to promote cohesion establishment that tethers 2 sister chromatids together. HDAC8 inhibition causes hyperacetylation of chromatin-bound SMC3, resulting in reduced cohesin mobility and accumulation of R-loops that block replisomes traveling on chromatin. This generates replication stress and activates checkpoint kinases to secure replication fork integrity. Inactivation of checkpoint activity in cancer cells further exacerbates replication stress to an intolerable level, leading to fork collapse and, thus, cancer-specific cytotoxicity.

Recent research has demonstrated that blocking checkpoint kinases can lead to synthetic lethality when combined with the inhibition of factors from the same pathway (ATR, CHK1, WEE1) or the chromatin assembly and DNA repair pathways (TLKs, BRCA2, PARP1, ERCC1-XPF, Fanconi anemia, p53) (21, 48). Here, we identified epigenetic agents that can synergize with checkpoint kinase inhibitors to exacerbate replication stress and degrade genome stability. We found that simultaneous inhibition of HDAC8 and checkpoint kinases generated substantial levels of DSBs during genome replication and induced an irreversible S-phase arrest, leading to synergistic cytotoxicity specifically in cancer cells. Mechanistically, we show that HDAC8 inactivation uncoupled SMC3 acetylation from fork progression, enhanced R-loop formation, impaired replication elongation, and ultimately induced replication-dependent DNA damage. These findings strongly indicate that HDAC8 activity is crucial for resolving R-loop structures and suppressing replication stress (Figure 9). This study has implications for widening the scope of replication stress–targeted therapy as part of a synthetic lethal response involving diverse mechanisms. The cancer-selective killing effect resulting from boosting replication stress is applicable to a wide variety of cancers, with minimal adverse effects on normal tissues. This further supports the concept that the vulnerability of cancer cells is due to their reliance on checkpoint pathways to tolerate high levels of intrinsic insults (2, 8, 10). Most important, the results from PDX and PDO models confirm our in vitro observations and provide critical insights into patient-specific drug responses and the tumor microenvironment, reassuring the reliability of our research.

Targeting HDACs is a promising strategy because these enzymes contribute to tumorigenesis by regulating various biological processes including transcription, metabolism, the DNA damage response, the cell cycle, apoptosis, protein degradation, and immunity (49). HDAC inhibition by pan-HDAC inhibitors has been shown to impair DNA replication, DNA repair, and genome stability (19, 50), which may partly be attributed to inhibition of HDAC8, as suggested by our results. However, the clinical failure of pan-HDAC inhibitors due to toxicity necessitates the design of more targeted drugs specific to individual HDAC subtypes (51). Given its high affinity for chelation of catalytic zinc ions in the active site, the hydroxamate moiety is used as the backbone for the majority of HDAC inhibitors, endowing them with high potential as effective compounds (52). Both PCI-34051 and HDAC8i-3 are hydroxamate derivatives that demonstrate high specificity toward HDAC8 (27). However, the hydroxamic acid group is particularly susceptible to metabolic inactivation by modifications such as glucuronidation (42, 43). We observed that PCI-34051 was rapidly metabolized in mice, resulting in only a small amount of intact compound remaining in circulating plasma, whereas HDAC8i-3 was markedly less susceptible to metabolism and is therefore more attractive for development for in vivo use. Even though HDAC8i-3 appears to be a superior advance over PCI-34051, more work needs to be conducted to optimize HDAC8 inhibition using non-hydroxamate-based HDAC8 inhibitors.

Subsequent validation of HDAC8 inhibitor and AZD-7762 combination in PDO, mouse xenograft, and PDX models showed high tumor suppression activity with a favorable tolerability profile (Figure 8 and Supplemental Figure 11). We detected high levels of pan-nuclear γH2AX staining in tumor samples (Figure 8, C and D), which is often observed in cells undergoing a high level of replication stress that results in global replication catastrophe (11). An early study showed that cells exhibiting high levels of replication stress and DNA damage eventually succumbed to irreversible cell death (53). Our in vitro analyses clearly showed that the combination of an HDAC8 inhibitor with checkpoint kinase inhibitors induced both DSBs and initiated apoptosis (Figures 1 and 3). Given that combination treatment efficiently reduced tumor size (Figure 8), we favor the view that tumor cells with pan-nuclear γH2AX were experiencing extremely high levels of genome instability and undergoing apoptosis. While we noted an accumulation of DNA damage in intestinal crypt cells of some mice in the combination treatment group, the length of the villi was not affected, and no DNA damage was detected in villus epithelial cells that were rapidly and continuously regenerated from crypt stem cells (Supplemental Figure 11). Accordingly, no diarrhea or food-intake problems occurred, indicating that the gastrointestinal tract remained functional. Similarly, in a study using WEE1 and ATR inhibitors, DNA damage in intestinal crypt cells of mice was also observed but resolved within a week of the last treatment (14). Consistently, we observed only low levels of DNA damage without increasing apoptosis in normal epithelial cells cotreated with PCI-34051 and AZD-7762, and proliferation resumed soon after drug removal (Figure 4). These important findings strongly support the principle that synthetic lethality induced by targeting replication stress is a safe and effective strategy for cancer therapy.

Many drugs used in combination with checkpoint kinase inhibitors are able to elicit replication stress but often generate intensive genotoxicity when used alone. Some drugs, such as nucleotides (gemcitabine, cytarabine), topoisomerases (topotecan), or poly (ADP-ribose) polymerases (PARPs) (olaparib), etc., target factors required for normal cell proliferation. Although these drugs can enhance the efficacy of checkpoint kinase inhibitors, irreversible unwanted effects have been observed in clinical trials (2). In contrast, HDAC8 function appears to be nonessential in humans. We found that HDAC8 inactivation impaired chromatin replication and thus slowed S-phase progression, but these effects were quickly reversed after drug removal (Figure 2). Importantly, coinhibition of checkpoint kinase and HDAC8 induced irreversible early S-phase arrest and triggered apoptosis specifically in cancer cells (Figure 4 and Supplemental Figure 5). Our in vivo studies further confirmed that this combination elicited substantial DNA damage in tumors but no remarkable toxicity in mouse tissues (Figure 8 and Supplemental Figure 11). Given that HDAC8 is upregulated in many cancer tissues (54, 55), HDAC8 inhibitors could be considered as top candidate agents to enhance the efficacy and safety of checkpoint kinase inhibitors in cancer therapeutics. While HDAC8 deficiency is associated with Cornelia de Lange syndrome, which occurs during early development (56), diseases linked to HDAC8 deficiency have not been identified in adults Nevertheless, the development of seizures and heart defects, both symptoms of Cornelia de Lange syndrome, will need to be monitored carefully when patients are treated in the clinic.

Exacerbating replication stress can trigger fork collapse and DSBs, ultimately leading to cell death. The widely used anticancer drug gemcitabine markedly boosts replication stress and thus easily exceeds the survival limit when high replication stress tumors are treated. Adding an ATR inhibitor only enhances the treatment response in low-replication-stress tumors (57). In contrast, HDAC8 inhibition induces relatively low replication stress, but when combined with checkpoint kinase inhibitors, it can elevate replication stress to a lethal level in various tumor types. Cancer cells often exhibit high levels of replication stress, making them key targets for checkpoint kinase inhibitors. We therefore anticipate that this approach will be effective across a wide range of cancers. We envision that cancer cells with chromosomal instabilities, including chromosome rearrangement, dysregulation in genome maintenance mechanisms (e.g., BRCA mutations), or aberrations in oncogene/tumor suppressor pathways, such as Myc amplification or loss of TP53 and Rb, will be more responsive to our combination treatment.

Through the genome-editing technology, we demonstrated that SMC3 hyperacetylation by HDAC8 inhibition was crucial for inducing replication and increasing the effectiveness of checkpoint kinase inhibitors. Surprisingly, except for the PANC-1 cells, the CRISPR-edited cells did not show mutations at the expected sites (K105/K106) (Supplemental Figure 8G). We observed several deletion/insertion mutations in this region. Some of these mutations caused frame-shift changes that altered the amino acid sequence and resulted in incorrect translation termination. This could explain why the SMC3 protein level was lower in SMC3-mutant cells. Our focus on in-frame mutations revealed intriguing findings (Supplemental Figure 8G). Clones 7 and 21 of MDA-MB-231 cells, which ultimately lost SMC3 acetylation, showed an extra lysine (K) inserted before the canonical acetylation sites (KKK). In contrast, clone 2, which still retained SMC3 acetylation, showed an extra threonine at the same site (TKK). Two DLD-1–mutant clones defective in SMC3 acetylation were missing 2 amino acids (G103/A104) before to the acetylation sites, with an additional point mutation K105Q detected in clone 14. These results suggest the importance of the sequence near the acetylation sites (K105/K106) for SMC3 acetylation. To test this hypothesis, we generated expression plasmids encoding Flag-tagged SMC3 WT and the mutant with an extra lysine ahead of the acetylation sites (KKK). After transfecting cells with the plasmids, we isolated the SMC3 protein complex using the Flag antibody. We found that the lysine insertion dramatically decreased the level of SMC3 acetylation without affecting its association with the partner SMC1 (Supplemental Figure 8H). These results revealed that the sequence near the acetylation sites plays a crucial role in SMC3 acetylation.

The S-phase–specific, ESCO2-mediated acetylation of SMC3 is important for efficient chromatin replication and cohesion establishment, and its loss leads to slowed fork progression and reduced sister chromatid cohesion, accompanied by increases in scattered chromosomes and chromosome breakage (32, 34, 58). Intriguingly, impaired DNA replication and activation of checkpoint kinases were also evident in cells with HDAC8 inactivation that enhanced SMC3 acetylation (this work and refs. 24, 25). Deacetylation of SMC3 by HDAC8 at the end of mitosis is required for the renewal of the cohesin complex on chromatin in the subsequent G_1_ phase (32, 35). Yet, in our cell-cycle analysis (Supplemental Figure 6), before HDAC8 inhibitor treatment, most cells were synchronized in early S phase when cohesin proteins had been loaded on chromatin. These observations indicate that the replication stress induced by HDAC8 inhibition was not a consequence of deficient cohesin renewal.

SMC3 can also be acetylated by ESCO1 in G_1_ to regulate the genome topological structure and gene expression (16). ESCO1 stabilizes cohesin complexes on chromatin, similar to the role of SMC3 acetylation in cohesion establishment during S phase (34–36), and promotes the formation of CTCF-marked loops (59, 60). While it is generally accepted that SMC3 acetylation antagonizes cohesin release from chromatin through wings apart-like protein homolog (WAPL) (16), the recent Hi-C study revealed that SMC3 acetylation by ESCO1 recruits PDS5A to restrict chromatin loop length and HDAC8-mediated SMC3 deacetylation enables loop enlargement (61). Cohesin complexes also locate at chromatin regions containing R-loops (38), and our study indicates that HDAC8 activity is required for R-loop resolution (Figure 7). The ssDNA present in an R-loop provides an interface for RPA binding, which subsequently recruits RNase H1 (62) and probably ATR/CHK1. This may explain the modest increase in RPA-coated ssDNA detected in cells with HDAC8 inhibition. Although other targets may be also involved in HDAC8-mediated suppression of replication stress, accumulation of R-loops in cells lacking HDAC8 function acted as an obstacle to fork progression. Overexpression of RNase H1 degraded R-loops and thereby minimized the DNA damage induced by inactivation of HDAC8 alone or in combination with checkpoint kinases (Figure 7). Our results further demonstrated that SMC3 hyperacetylation is key to R-loop accumulation resulting from HDAC8 inhibition (Figure 7). We thus propose that HDAC8 is required for erasure of the acetylation of SMC3 to promote the mobility of cohesin complexes ahead of replication forks. Loss of HDAC8 activity results in the generation of R-loops, presumably by reducing the switch of chromosomal cohesin during a state of high turnover, which blocks the migration of replisomes on chromatin. This leads to an increase in replication stress and activation of checkpoint kinases to protect fork stability. Subsequent checkpoint failure caused by inhibition of checkpoint kinases overwhelms the replication stress threshold of cancer cells. This triggers replication fork collapse and genome catastrophe, potentially due to exhaustion of the nuclear RPA pool (11, 12), ultimately leading to intolerable damage and cell death (Figure 9). In line with this model, ATR activity has been found to be critical for the suppression of R-loop–induced genomic instability and survival of cancer cells harboring high levels of R-loops (63).

In summary, our study shows that our phenotypic drug screen is a powerful approach to identify potent combinations targeting replication stress for cancer therapy. We validated a synergistic vulnerability between HDAC8 and checkpoint kinases that highlights a potentially valuable precision medicine approach to selectively target cancer. Our findings identified a critical function of HDAC8 in genome replication surveillance and highlight this pathway as a druggable target.

## Methods

### Sex as a biological variable.

Sex was not considered as a biological variable. All mice used in this study were female.

### Cell culturing.

Human osteosarcoma U-2 OS (Bioresource Collection and Research Centre [BCRC]), breast cancer MDA-MB-231 (BCRC), and cervical cancer HeLa (BCRC) cell lines were cultured in DMEM (Gibco, Thermo Fisher Scientific, 12800-017). The human colon cancer DLD-1 (BCRC) and non–small cell lung cancer H1299 (American Type Culture Collection [ATCC]) cell lines were cultured in RPMI 1640 medium (Gibco, Thermo Fisher Scientific, 23400-021). The CRC HCT-116 (ATCC) cell line was cultured in McCoy’s 5A medium (MilliporeSigma, M4892). The human H184B5F5/M10 (M10) (BCRC) normal breast epithelial cell line was cultured in MEM-α (Gibco, Thermo Fisher Scientific, 11900-024). All media above were supplemented with 10% FBS and 1% penicillin/streptomycin/glutamine (PSG). The human MCF-10A normal breast epithelial cell line was cultured in DMEM/F12 medium containing 5% horse serum, 1% PSG, 20 μg/L EGF (Peprotech, AF-100-15), 10 mg/L insulin, 500 μg/L hydrocortisone, 100 μg/L cholera toxin, and 1% nonessential amino acids. All cell lines were cultured in a humidified incubator at 37°C in an atmosphere of 5% CO_2_.

### MTT assay.

Cells grown in 96-well plates were treated with the indicated compounds for 24 hours and then released in the fresh culture medium. After 48 hours, cells were incubated with 1 mg/mL MTT (MilliporeSigma, M2128) for 4 hours. The precipitated formazan crystals were then dissolved in DMSO, and the absorbance at 490 nm was measured using the Victor^3^ 1420 Multilabel Counter (PerkinElmer).

### DNA fiber analysis.

MDA-MB-231 cells were treated with the indicated compounds for 6 hours and subsequently pulse-labeled sequentially with 25 μM chlorodeoxyuridine (CIdU) for 20 minutes and 200 μM iododeoxyuridine (IdU) for another 20 minutes. Labeled cells were harvested, and DNA fiber spreads were prepared and visualized as previously described (64). Microscopy was performed using a Keyence BZ-X810 all-in-one digital microscope, and images were taken from randomly selected fields. For structure analyses, the frequencies of the different dynamics of fiber tracks were classified as follows: red-green (elongating fork), red (stalled or terminated forks), green-red-green (first pulse origin), green (second pulse origin), and red-green-red (fork termination). For each condition, at least 300 forks were assessed from 3 independent experiments. For fork progression analysis, CldU and IdU track lengths of DNA fiber molecules were measured using ImageJ (NIH). A minimum of 100 individual fibers were analyzed for each experiment, and the means of at least 3 independent experiments are presented.

### Chromatin fractionation.

Chromatin fractionation was performed as described previously (21) with minor modifications. Soluble proteins were pre-extracted with 0.5% Triton X-100 for 5 minutes on ice. Chromatin-bound proteins were then lysed in 1× Laemmli sample buffer and subjected to Western blot analysis.

### Dot blots.

Genomic DNAs (gDNA) was extracted using the QIAamp DNA Mini Kit (QIAGEN). Equal gDNAs were spotted on a Zeta-Probe GT Membranes (Bio-Rad) using the Bio-Dot Microfiltration Apparatus (Bio-Rad) and cross-linked using Ultraviolet Crosslinkers (50 mJ/cm^2^ UV) (UVP CL-1000). The membrane was blocked with 5% skim milk, and DNA-RNA hybrids were probed with S9.6 antibody, followed by incubation with HRP-conjugated goat anti-mouse antibody. Chemiluminescence was assessed using the Western ECL Substrate kit (Bio-Rad) and measured by ImageQuant (LAS 4000).

### Generation of CRISPR/Cas9-edited cell lines.

SMC3-mutant cells were generated by transfecting parental cells with the sgRNA-containing px458 plasmid using Lipofectamine 2000 (Invitrogen, Thermo Fisher Scientific). Single GFP^+^ cells were sorted into 96-well plates 24 hours after transfection. Following 2 weeks of culturing, the clones were then expanded and analyzed for SMC3 expression and acetylation by immunoblot analysis. The sequence for sgSMC3 was: AAGAGTTATTGGTGCCAAAA.

The EdU incorporation assay, immunofluorescence staining, PFGE, trypan-blue exclusion assay, thymidine synchronization, and cell-cycle analysis were performed as described previously (21, 65). Detailed information can be found in the Supplemental Methods.

### Cancer organoid assay.

CRC PDOs were obtained from ATCC (HCM-CSHL-0257-C18) and were maintained according to the ATCC organoid culture guide. The PDOs were cultured using the Organoid Growth Kit 1A (ATCC, ACS-7100) supplemented with penicillin/streptomycin. The morphology of the PDOs was routinely checked to ensure their phenotypic characteristics. For experiments, the PDOs were processed and cultivated in rBM (growth factor–reduced Matrigel). After 3 days of culturing in 96-well plates, the PDOs were treated with the indicated compounds for 24 hours and then released into the culture medium for 48 hours. Following 2 cycles of the above treatment, the viability of the PDOs in each well was determined using CellTiter-Glo (Promega, G7570) following the manufacturer’s instructions. The luminescent signal was measured with a plate reader (SpectraMax iD5).

### IHC analysis.

Slides were deparaffinized in xylene and hydrated in a series of graded alcohol dilutions. Sections were then placed in 250 mL antigen-unmasking solution from Vector Laboratories for antigen retrieval in a pressure cooker at 123°C for 30 seconds and subsequently cooled on the benchtop for 20 minutes. Hydrogen peroxide (3%) was used to block endogenous peroxidase activity. Sections were placed in Rodent Block M solution for 30 minutes to block nonspecific background staining and then incubated with primary antibodies against γH2AX (Cell Signaling Technology, 9718S, 1:400 dilution) for 1 hour at room temperature. After the sections were rinsed, they were applied with Rabbit on Rodent HRP Polymer (Biocare Medical) for 30 minutes, and color development was performed using DAB. Counterstaining was accomplished by staining with CAT hematoxylin. All washing steps were performed in PBS alone and in PBS with 0.1% Tween-20.

### Statistics.

Cell survival data are presented as the mean ± SD. Mouse data are presented as the mean ± SEM. GraphPad Prism (GraphPad Software) was used to determine statistical significance. A 2-tailed paired *t* test was used to determine the *P* values of the results presented in SuperPlots (66), and a 1-way ANOVA test was applied for multiple comparisons. A *P* value of less than 0.05 was considered significant.

### Study approval.

Animal experiments were conducted according to a protocol reviewed and approved by an IACUC protocol at CWRU (Stanton Gerson, PI, protocol no. 2016-0049; and Rui Wang, PI, protocol no. 2019-0087). All animal studies were performed and handled in accordance with the CWRU IACUC protocols and regulations. For mouse xenograft models, 5 × 10^6^ viable DLD-1 or MDA-MB-231 cells were injected into the subcutaneous tissue of both dorsal flanks of 5- to 6-week-old female athymic nude (nu/nu) or NOD SCID γ (NSG) mice (The Jackson Laboratory). Cell numbers were established via trypan blue exclusion. For PDX model, tumors derived from established pancreatic PDX were explanted and cut into homogenate, suspended with isometric PBS solution mixed with Matrigel. Then, the homogenate was subcutaneously implanted into the lower dorsal flanks of female athymic nude mice. Mice were randomly assigned to treatment groups and injected intraperitoneally with vehicle control, AZD-7762, PCI-34051, HDAC8i-3, or a combination when the average tumor volume reached an appropriate size of approximately 100–200 mm^3^. The detailed treatment dose and schedule are described in the figure legends. Tumor sizes were measured with calipers and monitored 2–3 times weekly, and mouse weights were also assessed 3 times a week. On the last day of the study, 1 hour after drug treatment, 3 randomly selected mice from each group were sacrificed, tumors were excised, and tumor weights were measured. Tumor samples were preserved in 10% buffered formalin for further analysis. Formalin-fixed tumor samples were submitted to the CWRU Histology Core Lab for processing.

### Data availability.

Data are available in the Supporting Data Values file.

## Author contributions

TYC, YY, and SBL designed and conducted experiments and analyzed data. ZYY, MR, NZL, LHC, and YQ assisted with experiments and data collection. WZ was responsible for the IHC data analysis. HJT and WJH provided HDAC8 inhibitors. YY and ERC performed bioinformatics analysis. MLK designed and constructed the CRISPR plasmids. JJP assisted with CI calculations. SBL, RW, KKT, WEH, and SLG conceived and supervised the study. TYC, YY, and SBL prepared the manuscript. JJP and SBL revised the manuscript. All authors reviewed the manuscript. The order of the first two authors’ names was determined alphabetically.

## Supplementary Material

Supplemental data

Unedited blot and gel images

Supporting data values

## Version 1 10/22/2024

In-Press Preview

## Version 2 12/02/2024

Electronic publication
