# Supplementary material for: Combined HDAC8 and checkpoint kinase inhibition induces tumor-selective synthetic lethality in preclinical models
Source: J Clin Invest. 2024 Oct 22;134(23):e165448. doi: 10.1172/JCI165448 (PMC11601943; doi:10.1172/JCI165448)
Supplement: Supplemental data [file jci-134-165448-s162.pdf]

Supplementary Information

A phenotypic drug screen discovers HDAC8 inhibitors as potential adjuvants for checkpoint kinase inhibitors to induce replication stress and genotoxicity

In brief, we treated the reporter cell line U-2 OS stably expressing GFP-RPA1 and RFP-PCNA (1, 2) with compounds alone or in combination with the CHK1/2 inhibitor AZD-7762. The treated cells were pre-extracted to remove soluble proteins and stained for γH2AX (Supplemental Figure 1A). As positive controls (1, 3), treatment with hydroxyurea (HU), an inhibitor of nucleotide synthesis, or AZD-7762 alone slightly increased levels of both DNA damage and GFP-RPA1 bound on replicating chromatin, and the combined treatment generated synergistic effects for both phentypes (Supplemental Figure 1B).

Accordingly, we performed a high-throughput screen on a commercially available library comprising 152 epigenetic inhibitors (Cayman). In this screen, 24 compounds combined with AZD-7762 induced effects on DNA damage and RPA accumulation greater than the total power of individual treatments were selected as positive hits (Supplemental Figure 1C-D). As expected, 4 of the top 5 hits (Resminostat, Scriptaid, SP2509, and ITF 2357) are known to target histone deacetylases or demethylases that have been shown to regulate chromatin replication and genome integrity (4-8). Another top hit, PCI-34051, emerged as a particularly interesting candidate, as its specific target, HDAC8, is a unique protein deacetylase harboring substrates and functions distinct from other canonical class I HDACs (HDAC1, 2, and 3) (9).

HDAC8 is a Zn^{2+}-dependent deacetylase localized predominantly in the nucleus (9) and upregulated in many cancer tissues (10). Several studies have shown that RNAi-mediated depletion of HDAC8 in different types of cancer cells suppresses cell proliferation (11-13).

However, inactivation of HDAC8 activity by specific inhibitors seems to generate substantial cytotoxicity only in T-cell leukemia and neurologic cancers (14, 15). Although the loss of HDAC8 activity has been shown to perturb S-phase progression (16, 17), whether HDAC8 possesses a regulatory function in chromatin replication remains unexplored. Here, we showed that a short-term suppression of HDAC8 by PCI-34051 remarkably enhanced RPA-marked ssDNA accumulation and DNA damage in replicating cells treated with AZD-7762 (Supplemental Figure 1C-G). By checking a series of chemicals with reported inhibitory activity towards HDAC8 (Supplemental Table 2) (18), we further confirmed that HDAC8 inhibitors (HDAC8i) are promising candidates for synergistic combination since all tested compounds showed a consistent pattern of induction of GFP-RPA1 and γH2AX signals that occurred explicitly in replicating cells (Supplemental Figure 1F-G). To ensure the compounds were acting primarily on-target, we examined the specificity of the HDAC8 inhibitors. As expected, all these agents increased the acetylation level of SMC3 (structural maintenance of chromosomes protein 3), a well-established HDAC8 substrate (Supplemental Figure 1H) (19). Among them, PCI-34051, HDAC8i-1 and HDAC8i-3 displayed high specificity towards HDAC8, illustrated by the absence of elevated acetylation of α-tubulin (HDAC6) and histone H3 (class I/II HDACs) (Supplemental Figure 1H). These observations suggest that HDAC8 inactivation may induce replication stress and collaborate with checkpoint kinase inhibitors to generate synergistic toxicity in cancer cells.

Supplementary Materials and Methods

Chemicals and antibodies

The chemicals and antibodies used in this study are listed in Supplementary Table 1-3.

Epigenome compound screen

The U-2 OS reporter cell line with stable expression of GFP-RPA1/RFP-PCNA were seeded on 384-well plates and treated with individual compounds of Epigenetics Screening Library (Cayman, 11076) and the CHK1/2 inhibitor AZD-7762 for 6 h. Cells were pre-extracted with 0.5% Triton X-100 for 5 min and fixed with 2% formaldehyde for 15 min. The fixed cells were incubated with 3% bovine serum albumin (BSA), followed by immunofluorescence staining for γH2AX. Images were collected by Molecular Devices ImageXpress Micro XL microscope system with 10X Plan Fluor or 20X SWD20 Plan Fluor objective lens. MetaXpress® Power Core™ software was used for quantification.

Western blot analysis

Cells were harvest and lysed in 1x Laemmli sample buffer (LSB; 60 mM Tris-HCl pH 6.8, 2% SDS and 10% glycerol). Equal protein amounts were separated by 12% SDS-polyacrylamide gel electrophoresis (PAGE) and transferred onto nitrocellulose membrane. After blocking with 5% skim milk, the membranes were incubated with specific primary antibodies, followed by incubation with species-specific horseradish peroxidase (HRP)-conjugated secondary antibodies. Chemiluminescence were detected by using the ECL Substrate kit (Bio-Rad) and measured by ImageQuant™ (LAS 4000).

EdU-incorporation assay and immunofluorescence staining

DNA replication efficiency was determined by the Click-iT™ EdU Cell Proliferation Kit (Invitrogen, C10340) according to the manufacturer's instructions. Briefly, cells were treated with indicated compounds for 6 h and pulsed with 20 μM of EdU for 30 min. Cells were pre-extracted with 0.5% Triton X-100 for 5 min and fixed with 2% formaldehyde for 15 min, followed by incubation with the Click-iT reaction for 30 min. Cells were then incubated with ice-cold methanol for 15 min for the antigen retrieval of PCNA. After blocking with 3% BSA, cells were incubated with primary antibodies at 4°C for overnight and then incubated with species-specific secondary antibodies at room temperature for 1 h. Finally, nuclei were stained by incubation with 5 μg/ml of 2-(4-amidinophenyl)-6-indolecarbamidine dihydrochloride (DAPI; Sigma, D9542).

Pulsed-field gel electrophoresis (PFGE)

PFGE was performed as described previously (20) with minor modifications. Briefly, cells grown on 10-cm dishes were treated with indicated compounds for 15 h. 1 x 10^6 treated cells were molded into 1% CleanCut Agarose (BioRad, 1703594) plugs, followed by incubation with the buffer containing 10 mM tris-Cl (pH 8.0), 50 mM EDTA, 1% N-laurylsarcosyl, 100 μg/ml proteinase K at 50°C for 72 h. Plugs were then subjected to PFGE (1% agarose; 120° angle, 60- to 240-s switch time, and 4 V/cm) (Bio-Rad Laboratories) for 20 h. Gels were stained with ethidium bromide and imaged using Molecular Imager VersaDoc MP4000 system. The intensities of broken DNA were measured by using the Multi-Gauge software. Relative amounts of broken DNAs were calculated by normalizing the values of treated groups to that of the untreated control, and results from three biological repeats are shown.

siRNA transfection

Cells grown on 6-well plates were transfected with specific small interference RNAs (siRNAs) at a final concentration of 50 nM (Sigma), 25 nM (Dharmacon), or 20 nM (Ambion), using Lipofectamine 3000 or RNAiMAX Reagent (Thermo) according to the manufacturer's instructions. siHDAC8 #1 was purchased from Sigma and the designed sequence (5'-GUCCCGAGUAUGUCAGUAU-3') was referenced to Yen et al (21); siHDAC8 #2 was purchased from Dharmacon (Smartpool, L-003500-00-0005); siCDC45 (sequence: 5'-CGGAUCUCCUUUGAGUAUG-3') was purchased from Sigma; siESCO1 was purchased from Ambion (s41632).

Trypan-blue exclusion assay

Cells grown on 6-well plates were treated with indicated compounds for 24 h and released in fresh culture medium. At the indicated time points, cells were collected and stained with trypan blue. The amount of viable and death cells was measured using the Vi-CELL XR Viability Analyzer (Beckman Coulter).

Cell cycle analysis

At each designated time point, cells were collected and fixed with 70% ice-cold ethanol. The fixed cells were washed with cold PBS containing 1% FBS and stained with 0.05 mg/ml propidium iodide (PI) containing 0.25 mg/ml RNase A at 37 °C for 30 min, followed by measurement of DNA content using BD FACSCalibur. The cell-cycle profiles were plotted and percentage of sub-G_1 population were quantified by the FlowJo software.

Thymidine synchronization

Thymidine synchronization was performed as described previously (20) with minor modifications. Cells were incubated with 2 mM of thymidine for 20 h. Cells were then washed

strictly and incubated with fresh culture medium. At indicated time points, cells were then treated with indicated compounds and harvested accordingly.

Thymidine-nocodazole synchronization

Cells were incubated with 2 mM of thymidine for 20 h. Cells were then washed and released into culture medium for 4 h, followed by treating with 150 ng/ml nocodazole for 12 h to block cells in mitosis. The mitotic cells were de-attached by shaking and tapping and then washed strictly. The equal mitotic cells amount was seeded and incubated with culture medium.

Plasmids

The plasmids pcDNA3.1-SMC3-Flag/V5-His, which express C-terminally Flag/V5-tagged SMC3, were constructed by inserting the corresponding cDNA into the pcDNA3.1/V5-His TOPO vector using TOPO-TA cloning (Thermo Fisher Scientific). The pcDNA3.1-SMC3-Flag/V5-His series, which express mutant SMC3 containing K/T insertion before K105, were generated by introducing K/T insertion into pcDNA3.1-SMC3-Flag/V5-His using the GenBuilder™ DNA Assembly kit (GeneScript). Primers used for constructing the SMC3 mutants are: KK_F AAAAAGGATCAGTATTTCTTAGAC; TKK_R ATACTGATCCTTTTTtgtGGCACCAA-TAACTCTTCG; KKK_R ATACTGATCCTTTTTtttGGCACCAATAACTCTTCG; pcDNA3.1-HindIII_F ACCCAAGCTGGCTAGTTAAGC; SMC3-HindIII_R AGACTCCAT-AGCATGCAAGC.

LC-MS/MS

PCI-34051 and 22d and their glucuronides in mouse plasma were identified and analyzed using a liquid chromatography with tandem mass spectrometry (LC-MS/MS) method. Plasma was thawed and 5 µL was mixed with 5 µl of water. To precipitate protein and extract compounds of

interest, 90 µL cold methanol was added and vortexed for 15 minutes at 2000 rpm. The solution was centrifuged at 16,000 x g for 30 minutes. Two microliters of supernatant were used for LC-MS/MS analysis. All experiments were performed on an Agilent ZORBAX XDB-Phenyl column (50 x 2.1 mm, 5µm) using a Waters ACQUITY UPLC H-Class PLUS System. Compounds were eluted with a step gradient of solvents A and B (solvent A: 0.1% formic acid in water; solvent B: 0.1% formic acid in acetonitrile) at a flow rate of 0.3 ml/min. A multi-step gradient was applied as follows: an isocratic gradient of 10% solvent B for 2 minutes, increase from 10 to 70% B in 0.1 minutes, followed by a one-minute isocratic elution. The column was subsequently washed for 7 minutes at 95% B and re-equilibrated at 10% B for 5 minutes. Data were acquired in a Thermo Scientific Q Exactive Plus mass spectrometer using an electrospray ionization (ESI) positive mode parallel reaction monitoring (PRM) scan. The resolution was set at 17,500 (at 200 m/z), the AGC target at 2×105, maximum fill time 100ms, and the individual isolation window of 0.4 m/z. Normalized collision energy of 35 was employed for PCI-34051 and its glucuronide fragmentation and CE 20 was used for 22d and its glucuronide fragmentation. The PRM monitored precursors for PCI-34051, PCI-34051-glucuronide, 22d, and 22d-glucuronied were 297.123 m/z, 473.155 m/z, 346.144 m/z, and 522.176 m/z respectively. Data were analyzed by Thermo Scientific Xcalibur software.

Supplementary Table 1. Information of chemicals

Chemicals	Brand	Cat. No.
Epigenetics Screening Library	Cayman	11076
PCI-34051	BioVision	2251
AZD-7762	AdooQ	A100113
Hydroxyurea (HU)	Sigma	H8627
MK-1775	AdooQ	A10599
Nocodazole	Sigma	M1404
Palbociclib	AdooQ	A14427
Prexasertib	MedChemExpress	HY-18174
Roscovitine	LC Laboratories	R-1234
UCN-01	Sigma	U6508
VE-821	AdooQ	A11605

Supplementary Table 2. Information of HDAC8 inhibitors (18, 22, 23)

No.	Name	Formula	Structure
HDAC8i-1	(E)-N-Hydroxy-2-(4-fluorophenyl)-4-methoxycinnamide	$C_{16}H_{14}FNO_3$	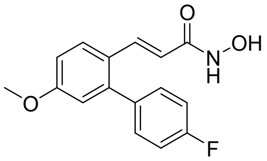
HDAC8i-2	(*E*)-*N*-Hydroxy-2-(2-quinolin-3-yl)-4-methoxycinnamide	$C_{19}H_{16}N_2O_3$	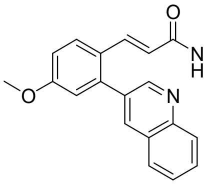
HDAC8i-3	(*E*)-*N*-Hydroxy-4-methoxy-2-(biphenyl-4-yl)cinnamide	$C_{22}H_{19}NO_3$	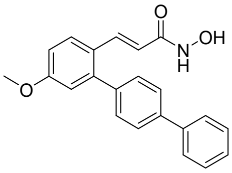
HDAC8i-4	(*E*)-*N*-Hydroxy-4-methoxy-2-(3,4-methylenedioxyphenyl)cinnamide	$C_{17}H_{15}NO_5$	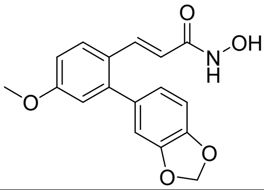
HDAC8i-5	(*E*)-*N*-hydroxyHydroxy-4-methoxy-3-(2-methoxyphenylamino)cinnamide	$C_{17}H_{18}N_2O_4$	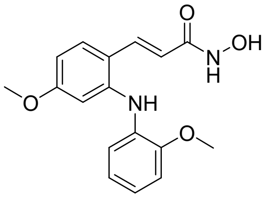
HDAC8i-6	*N*-Hydroxy-2-(4’-methoxybiphenyl-4-yl)-4-methoxycinnamide	$C_{23}H_{21}NO_4$	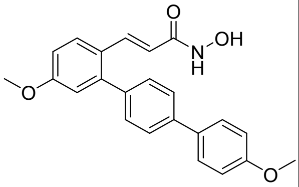

Supplementary Table 3. Information of antibodies

Antibodies	Brand	Cat. No.	Application
rabbit anti-PCNA	GeneTex	GTX100539	IF
mouse anti-γH2AX	Millipore	05-636	IF/WB
goat anti-rabbit	Jackson ImmunoResearch	111-295-144	IF
goat anti-mouse	Jackson ImmunoResearch	115-545-146	IF
mouse anti-actin	Sigma	MAB1501	WB
mouse anti-ATM pS1981	BD	56007	WB
mouse anti-ATM pS1981	Millipore	05-740	WB
rabbit anti-Bcl-xL	Epitomics	1018-S	WB
rabbit anti-caspase-3	Cell signaling	9662	WB
rabbit anti-caspase-8	Cell signaling	9746	WB
rabbit anti-caspase-9	Cell signaling	9502	WB
mouse anti-CDC45	Santa Cruz	sc-55569	WB
rabbit anti-CHK1 pS345	Cell signaling	2358	WB
rabbit anti-CHK2 pT68	Cell signaling	2661	WB
rabbit anti-CHK2	Cell signaling	2662	WB
rabbit anti-H2AX	Cell signaling	2595	WB
rabbit anti-H2A	Abcam	ab18255	WB
rabbit anti-HDAC8	Proteintech	17548-1-AP	WB
rabbit anti-H3 pS10	Millipore	06-570	WB
rabbit anti-H3 acK9	Millipore	07-352	WB
rabbit anti-H3	Abcam	ab1791	WB
goat anti-MCM2	Bethyl	A300-122A	WB

rabbit anti-KAP1 pS824	Abcam	ab70369	WB
rabbit anti-MCL-1	Abcam	ab32087	WB
rabbit anti-p53 pS15	Cell signaling	9284	WB
mouse anti-PCNA	Santa Cruz	sc-56	WB
rabbit anti-RPA1	Abcam	ab79398	WB
rabbit anti-RPA2 pS4/S8	Bethyl	A300-245A	WB
rabbit-RPA2 pS33	Bethyl	A300-246A	WB
mouse anti-RPA2	Thermo	MA1-26418	WB
mouse anti-SMC3 acK105/106	Millipore	MAB#1073	WB
rabbit anti-SMC3	Bethyl	A300-060A	WB
mouse anti-α-Tubulin acK40	Sigma	T7451	WB
mouse anti-α-Tubulin	Sigma	T5168	WB
goat anti-mouse	Jackson ImmunoResearch	115-035-003	WB/Dot blot
goat anti-rabbit	Jackson ImmunoResearch	111-035-003	WB
donkey anti-goat	GeneTex	GTX232040-1	WB
S9.6	Kerafast	ENH001	Dot blot

Supplementary References

1. Feng Y, Vlassis A, Roques C, Lalonde ME, Gonzalez-Aguilera C, Lambert JP, et al. BRPF3-HBO1 regulates replication origin activation and histone H3K14 acetylation. *EMBO J.* 2016;35(2):176-92.
2. Mejlvang J, Feng Y, Alabert C, Neelsen KJ, Jasencakova Z, Zhao X, et al. New histone supply regulates replication fork speed and PCNA unloading. *The Journal of cell biology.* 2014;204(1):29-43.
3. Toledo L, Neelsen KJ, and Lukas J. Replication Catastrophe: When a Checkpoint Fails because of Exhaustion. *Mol Cell.* 2017;66(6):735-49.
4. Bhaskara S. Histone deacetylases 1 and 2 regulate DNA replication and DNA repair: potential targets for genome stability-mechanism-based therapeutics for a subset of cancers. *Cell Cycle.* 2015;14(12):1779-85.
5. Wu R, Wang Z, Zhang H, Gan H, and Zhang Z. H3K9me3 demethylase Kdm4d facilitates the formation of pre-initiative complex and regulates DNA replication. *Nucleic Acids Res.* 2017;45(1):169-80.
6. Summers AR, Fischer MA, Stengel KR, Zhao Y, Kaiser JF, Wells CE, et al. HDAC3 is essential for DNA replication in hematopoietic progenitor cells. *J Clin Invest.* 2013;123(7):3112-23.
7. He Y, Zhao Y, Wang L, Bohrer LR, Pan Y, Wang L, et al. LSD1 promotes S-phase entry and tumorigenesis via chromatin co-occupation with E2F1 and selective H3K9 demethylation. *Oncogene.* 2018;37(4):534-43.
8. Jorgensen S, Schotta G, and Sorensen CS. Histone H4 lysine 20 methylation: key player in epigenetic regulation of genomic integrity. *Nucleic Acids Res.* 2013;41(5):2797-806.
9. Chakrabarti A, Oehme I, Witt O, Oliveira G, Sippl W, Romier C, et al. HDAC8: a multifaceted target for therapeutic interventions. *Trends Pharmacol Sci.* 2015;36(7):481-92.
10. Nakagawa M, Oda Y, Eguchi T, Aishima S, Yao T, Hosoi F, et al. Expression profile of class I histone deacetylases in human cancer tissues. *Oncol Rep.* 2007;18(4):769-74.
11. Song S, Wang Y, Xu P, Yang R, Ma Z, Liang S, et al. The inhibition of histone deacetylase 8 suppresses proliferation and inhibits apoptosis in gastric adenocarcinoma. *Int J Oncol.* 2015;47(5):1819-28.

12. Vannini A, Volpari C, Filocamo G, Casavola EC, Brunetti M, Renzoni D, et al. Crystal structure of a eukaryotic zinc-dependent histone deacetylase, human HDAC8, complexed with a hydroxamic acid inhibitor. *Proc Natl Acad Sci U S A.* 2004;101(42):15064-9.
13. Higuchi T, Nakayama T, Arao T, Nishio K, and Yoshie O. SOX4 is a direct target gene of FRA-2 and induces expression of HDAC8 in adult T-cell leukemia/lymphoma. *Blood.* 2013;121(18):3640-9.
14. Balasubramanian S, Ramos J, Luo W, Sirisawad M, Verner E, and Buggy JJ. A novel histone deacetylase 8 (HDAC8)-specific inhibitor PCI-34051 induces apoptosis in T-cell lymphomas. *Leukemia.* 2008;22(5):1026-34.
15. Rettig I, Koeneke E, Trippel F, Mueller WC, Burhenne J, Kopp-Schneider A, et al. Selective inhibition of HDAC8 decreases neuroblastoma growth in vitro and in vivo and enhances retinoic acid-mediated differentiation. *Cell Death Dis.* 2015;6:e1657.
16. Dasgupta T, Antony J, Braithwaite AW, and Horsfield JA. HDAC8 Inhibition Blocks SMC3 Deacetylation and Delays Cell Cycle Progression without Affecting Cohesin-dependent Transcription in MCF7 Cancer Cells. *J Biol Chem.* 2016;291(24):12761-70.
17. Lopez G, Bill KL, Bid HK, Braggio D, Constantino D, Prudner B, et al. HDAC8, A Potential Therapeutic Target for the Treatment of Malignant Peripheral Nerve Sheath Tumors (MPNST). *PLoS One.* 2015;10(7):e0133302.
18. Qi J, Singh S, Hua WK, Cai Q, Chao SW, Li L, et al. HDAC8 Inhibition Specifically Targets Inv(16) Acute Myeloid Leukemic Stem Cells by Restoring p53 Acetylation. *Cell Stem Cell.* 2015;17(5):597-610.
19. Deardorff MA, Bando M, Nakato R, Watrin E, Itoh T, Minamino M, et al. HDAC8 mutations in Cornelia de Lange syndrome affect the cohesin acetylation cycle. *Nature.* 2012;489(7415):313-7.
20. Lee SB, Segura-Bayona S, Villamor-Paya M, Saredi G, Todd MAM, Attolini CS, et al. Tousled-like kinases stabilize replication forks and show synthetic lethality with checkpoint and PARP inhibitors. *Sci Adv.* 2018;4(8):eaat4985.
21. Yan W, Liu S, Xu E, Zhang J, Zhang Y, Chen X, et al. Histone deacetylase inhibitors suppress mutant p53 transcription via histone deacetylase 8. *Oncogene.* 2013;32(5):599-609.

22. Huang WJ, Wang YC, Chao SW, Yang CY, Chen LC, Lin MH, et al. Synthesis and biological evaluation of ortho-aryl N-hydroxycinnamides as potent histone deacetylase (HDAC) 8 isoform-selective inhibitors. *ChemMedChem.* 2012;7(10):1815-24.
23. Kuo YH, Huang WJ, and Chang CI. United States; 2019.
