# Supplementary material for: Combined HDAC8 and checkpoint kinase inhibition induces tumor-selective synthetic lethality in preclinical models
Source: J Clin Invest. 2024 Oct 22;134(23):e165448. doi: 10.1172/JCI165448 (PMC11601943; doi:10.1172/JCI165448)

Figure 1A

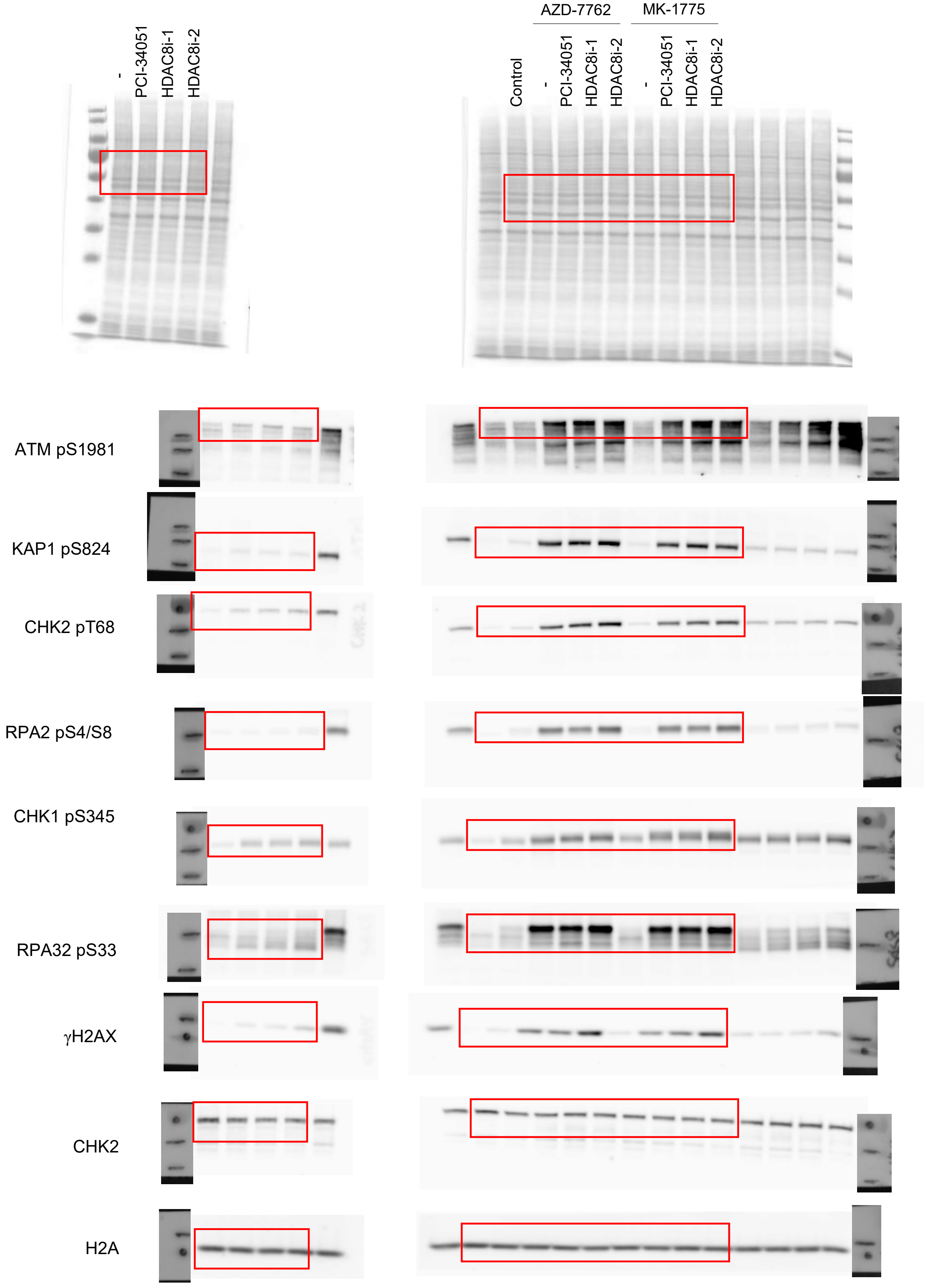

-
PCI-34051
HDAC8i-1
HDAC8i-2
AZD-7762
MK-1775
Control
-
PCI-34051
HDAC8i-1
HDAC8i-2
-
PCI-34051
HDAC8i-1
HDAC8i-2
ATM pS1981
KAP1 pS824
CHK2 pT68
RPA2 pS4/S8
CHK1 pS345
RPA32 pS33
γH2AX
CHK2
H2A

Figure 1B

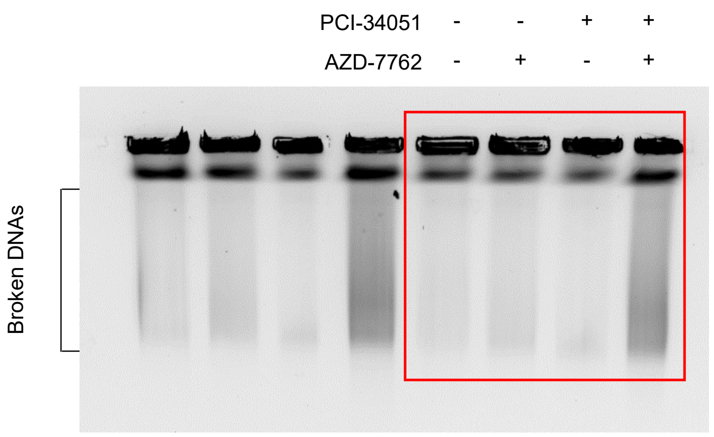

Figure 1C

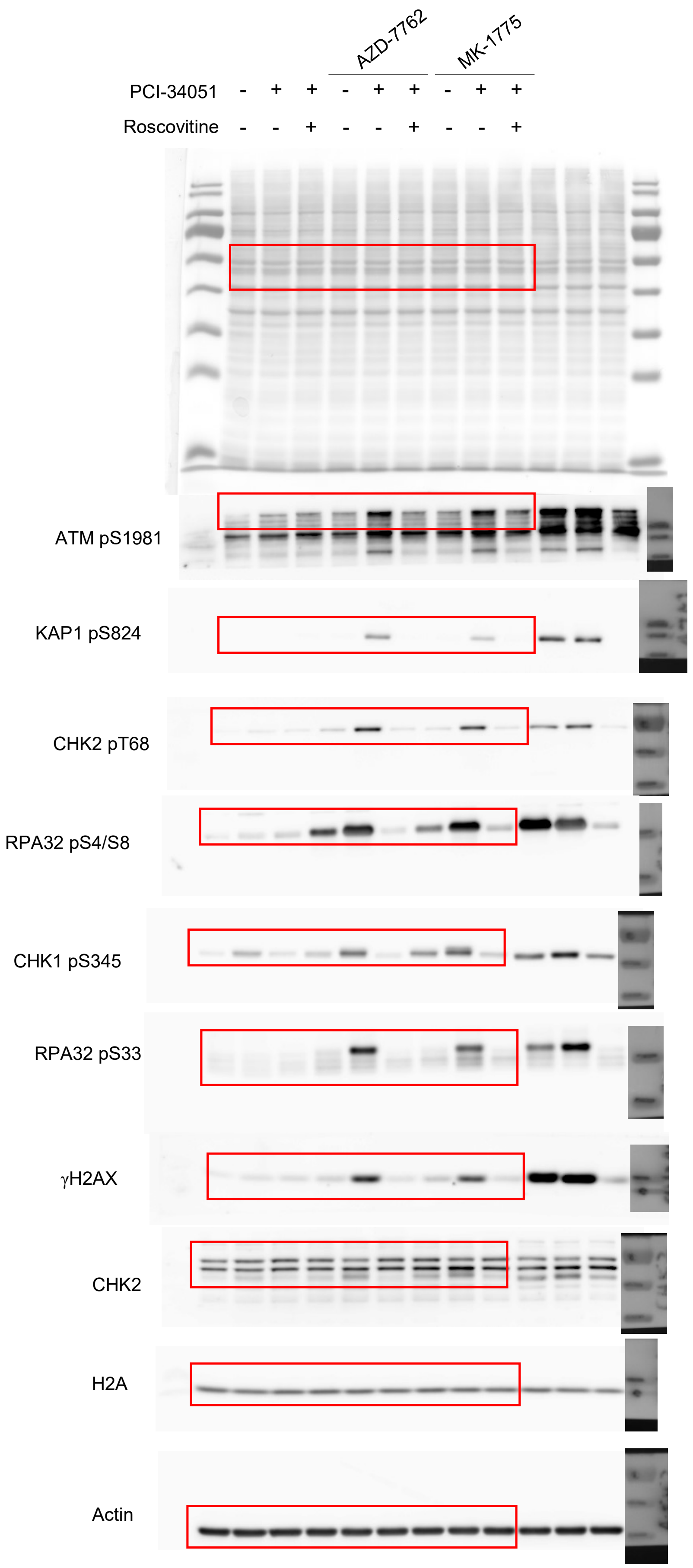
AZD-7762
MK-1775
PCI-34051 - + + - + + - + +
Roscovitine - - + - - + - - +
ATM pS1981
KAP1 pS824
CHK2 pT68
RPA32 pS4/S8
CHK1 pS345
RPA32 pS33
γH2AX
CHK2
H2A
Actin

Figure 2D

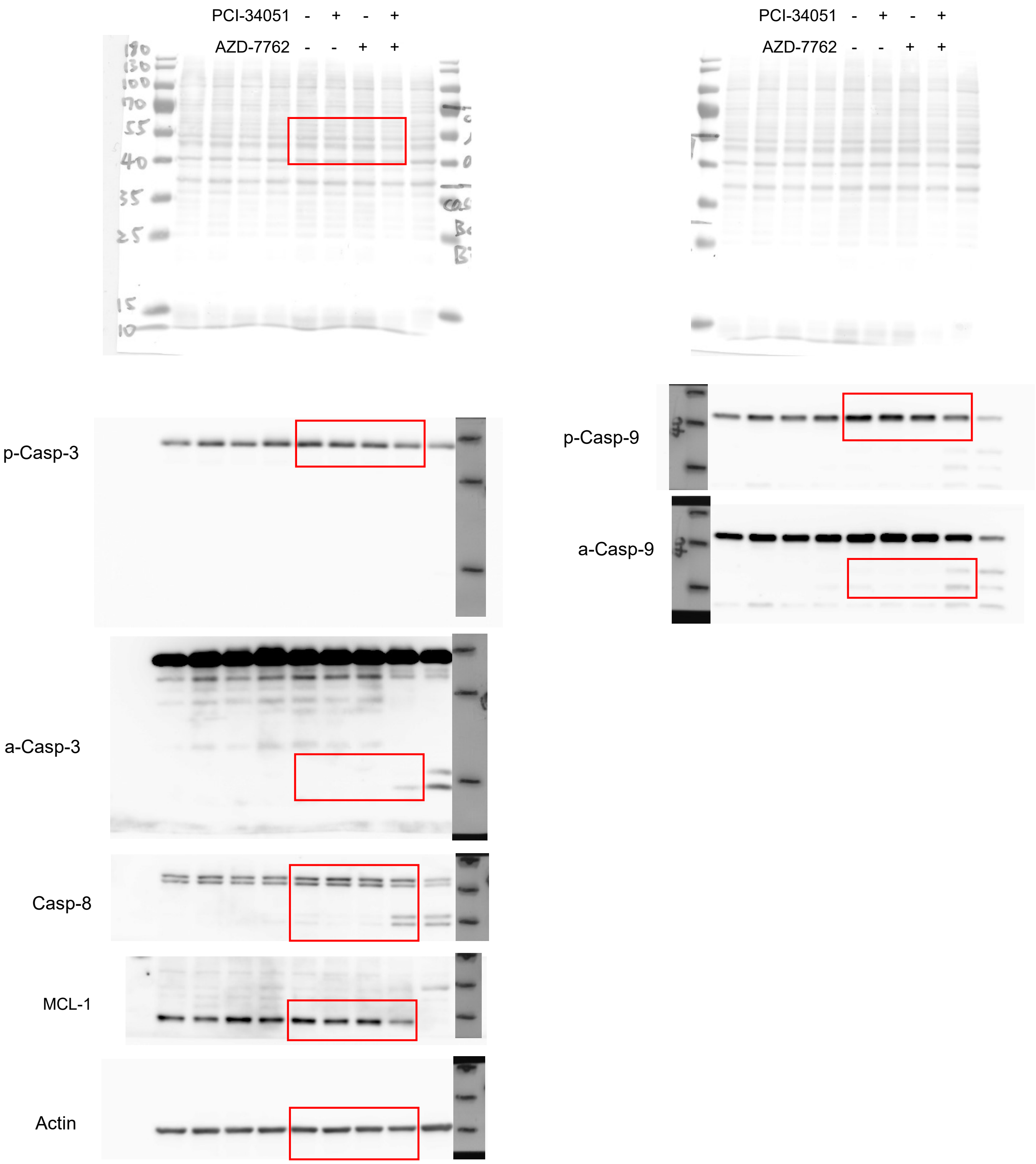

PCI-34051 - + - +
AZD-7762 - - + +
PCI-34051 - + - +
AZD-7762 - - + +
p-Casp-3
a-Casp-3
Casp-8
MCL-1
Actin
p-Casp-9
a-Casp-9

Figure 4C

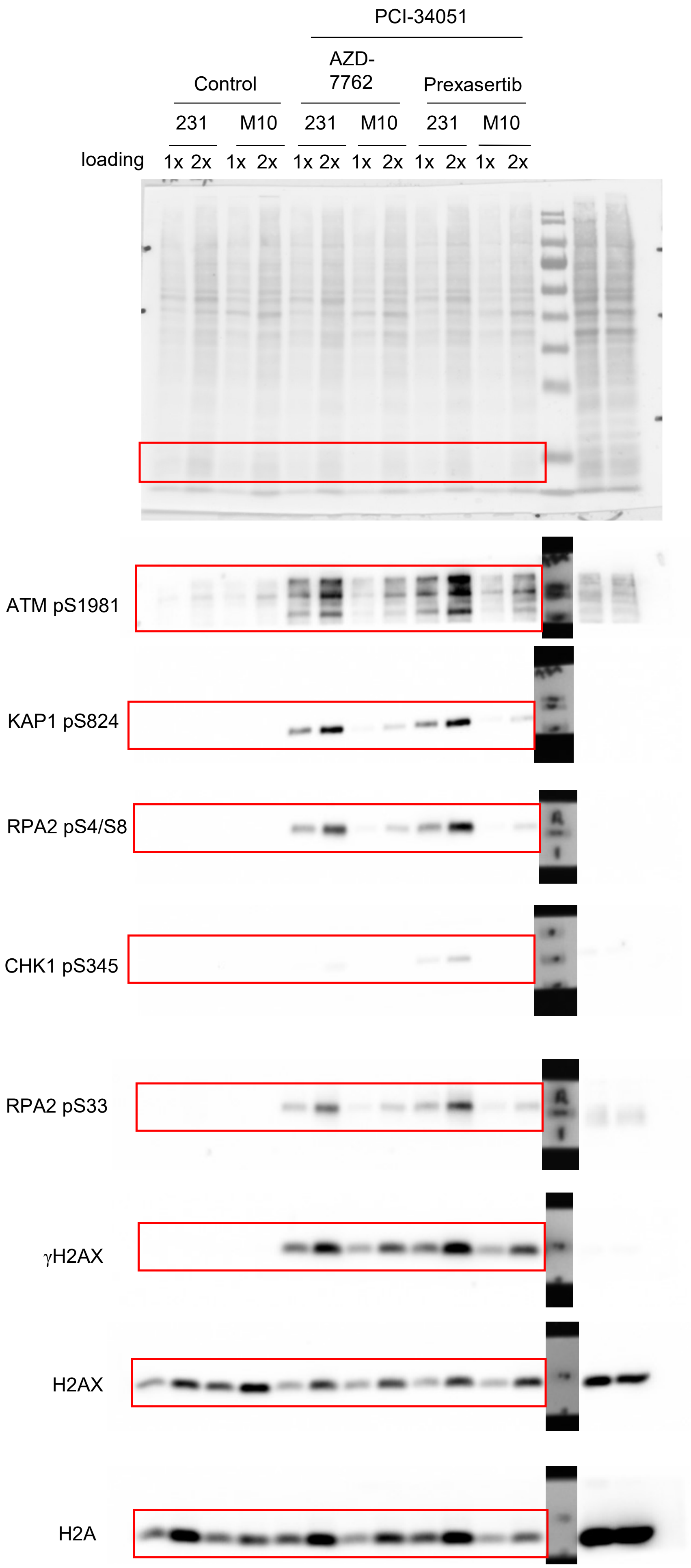
PCI-34051
Control
AZD-7762
Prexasertib
231
M10
231
M10
231
M10
loading
1x 2x 1x 2x 1x 2x 1x 2x 1x 2x 1x 2x
ATM pS1981
KAP1 pS824
RPA2 pS4/S8
CHK1 pS345
RPA2 pS33
γH2AX
H2AX
H2A

Figure 4D

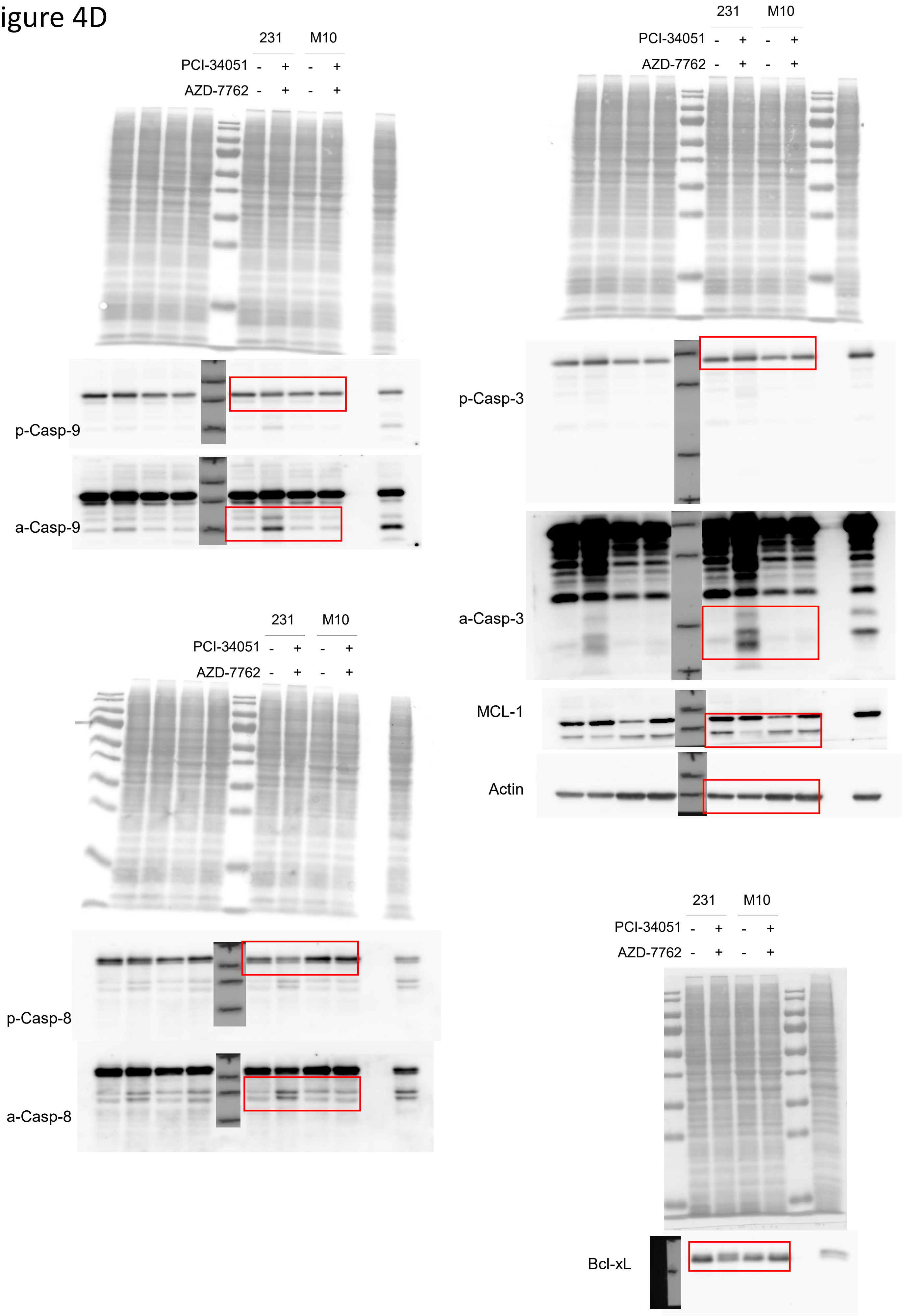
231
M10
PCI-34051 - + - +
AZD-7762 - + - +
p-Casp-9
a-Casp-9
p-Casp-8
a-Casp-8
p-Casp-3
a-Casp-3
MCL-1
Actin
Bcl-xL

Figure 5B

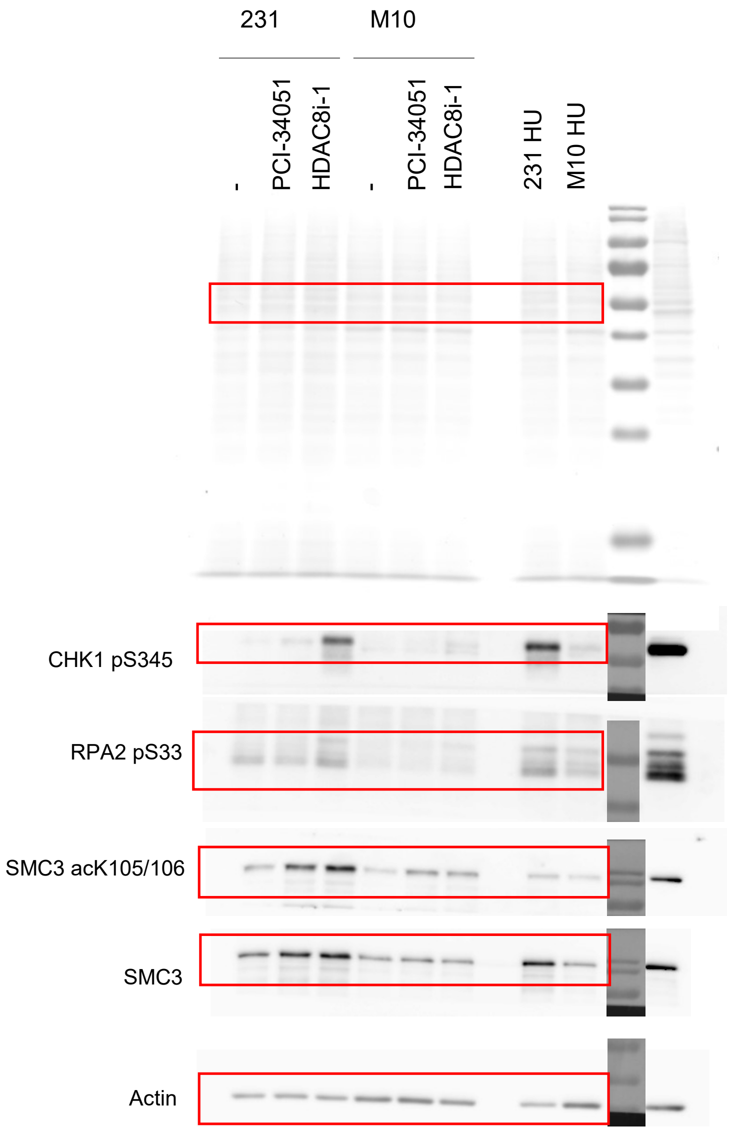
231
M10
-
PCI-34051
HDAC8i-1
-
PCI-34051
HDAC8i-1
231 HU
M10 HU
CHK1 pS345
RPA2 pS33
SMC3 acK105/106
SMC3
Actin

Figure 6A

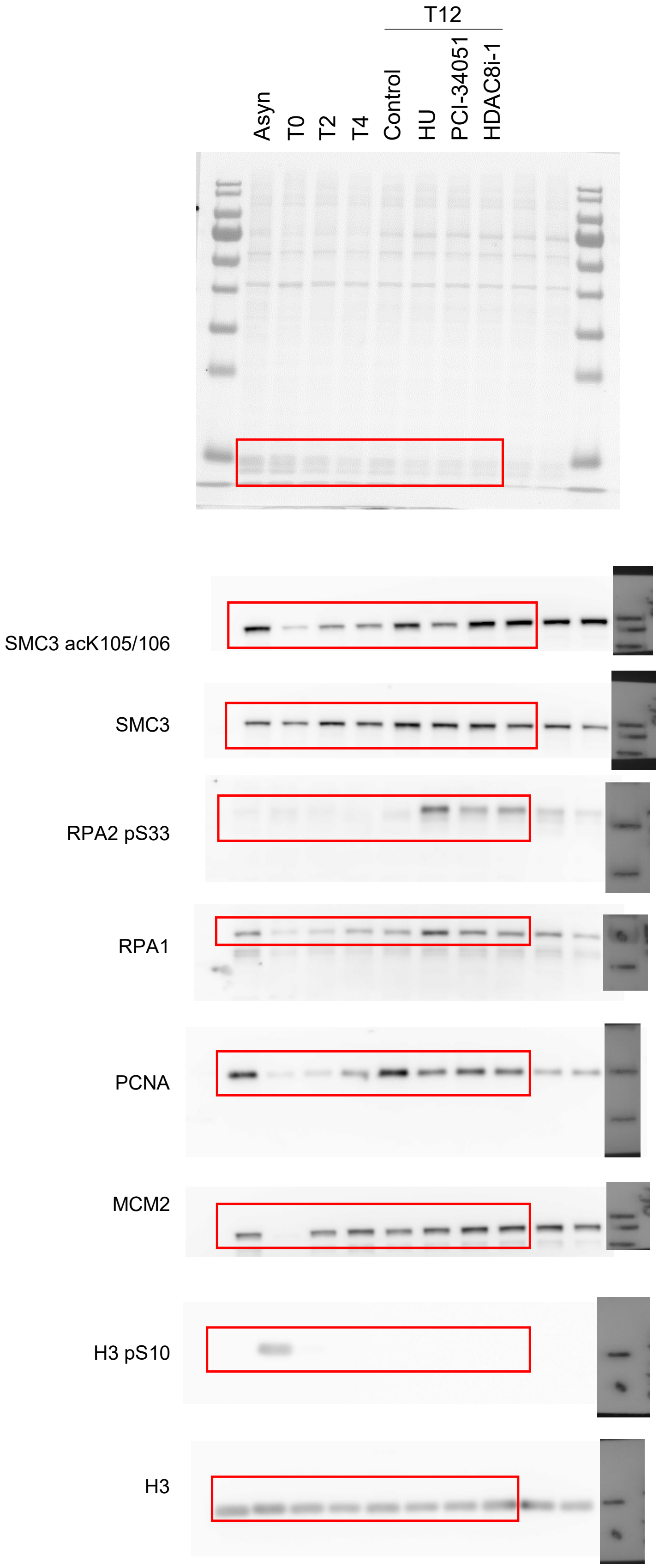
T12
Asyn
T0
T2
T4
Control
HU
PCI-34051
HDAC8i-1
SMC3 acK105/106
SMC3
RPA2 pS33
RPA1
PCNA
MCM2
H3 pS10
H3

Figure 6B

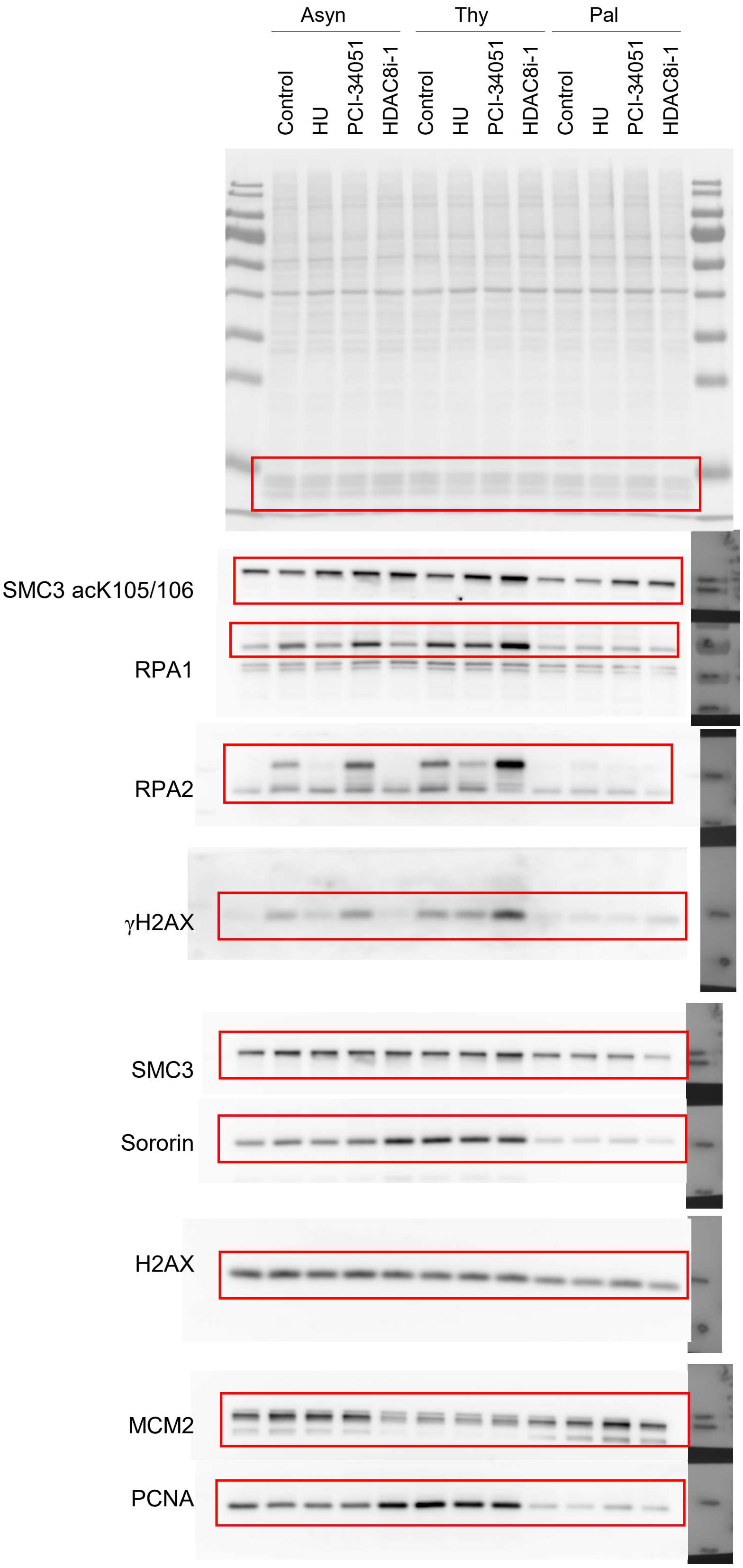

Asyn
Thy
Pal
Control
HU
PCI-34051
HDAC8i-1
Control
HU
PCI-34051
HDAC8i-1
Control
HU
PCI-34051
HDAC8i-1
SMC3 acK105/106
RPA1
RPA2
γH2AX
SMC3
Sororin
H2AX
MCM2
PCNA

Figure 7A

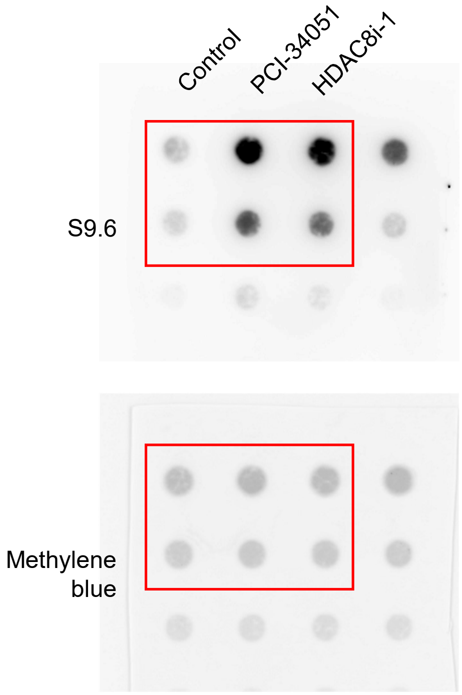

Figure 7B

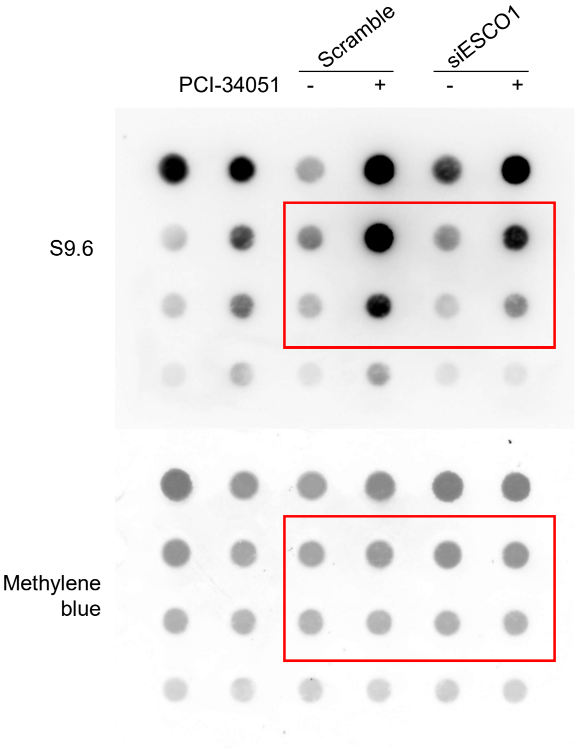

Figure 7C

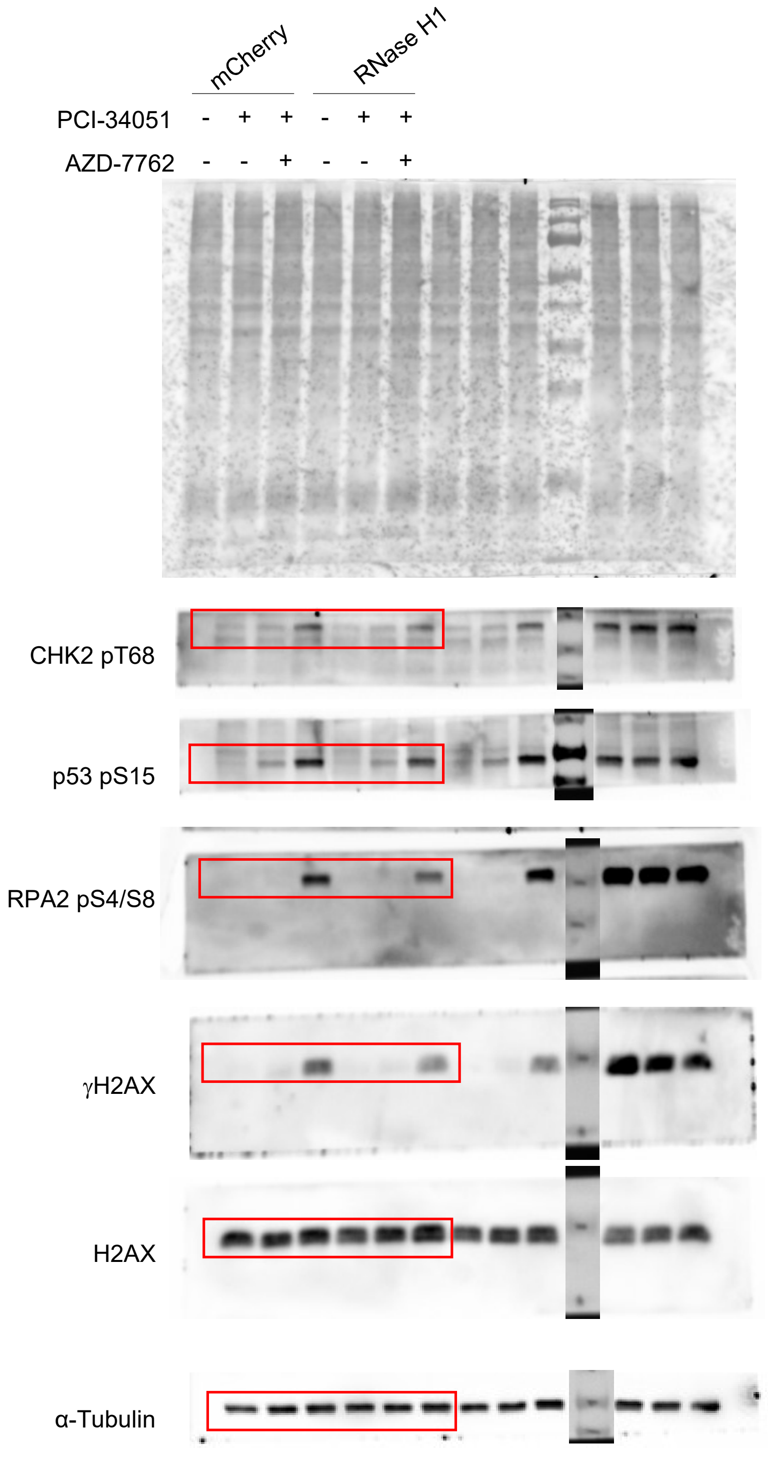

mCherry
RNase H1
PCI-34051 - + + - + +
AZD-7762 - - + - - +
CHK2 pT68
p53 pS15
RPA2 pS4/S8
γH2AX
H2AX
α-Tubulin

Figure 7D

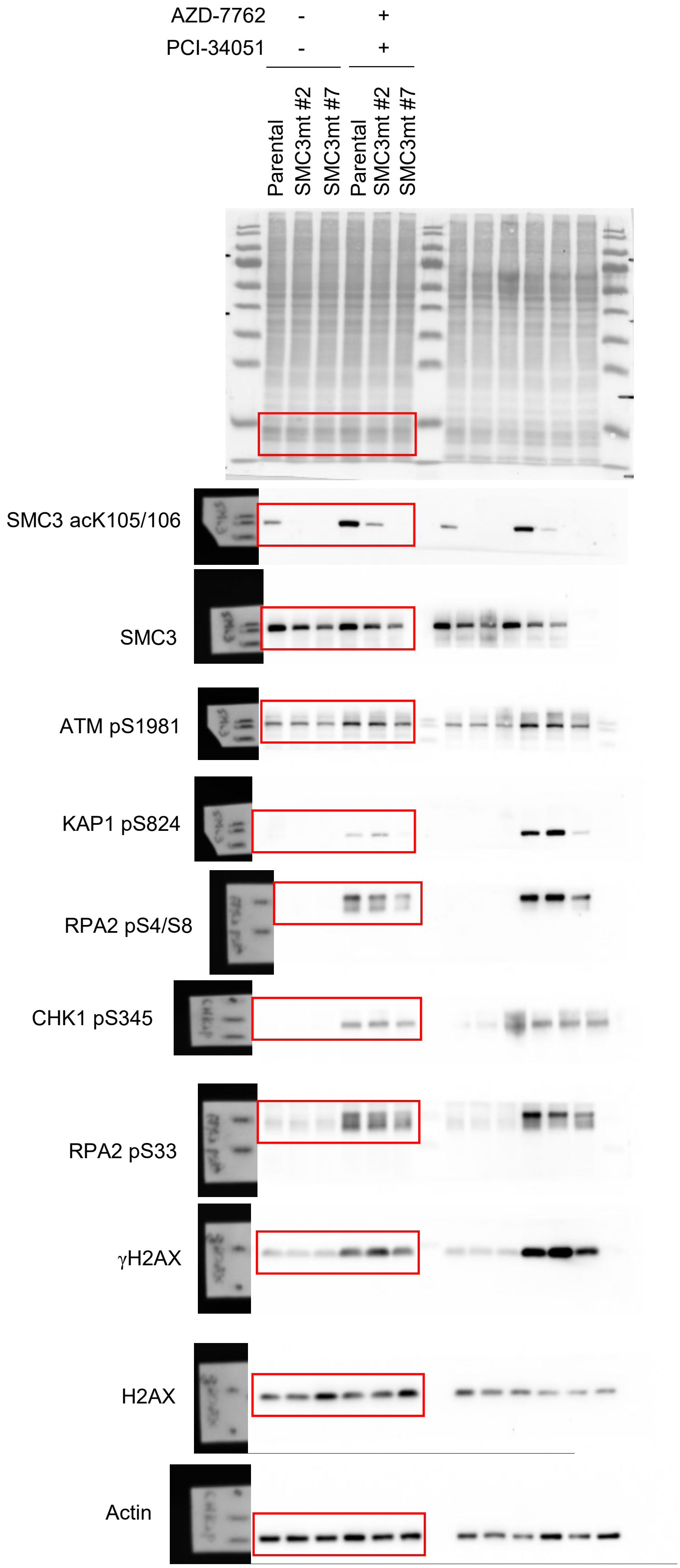

AZD-7762 - +
PCI-34051 - +
Parental
SMC3mt #2
SMC3mt #7
Parental
SMC3mt #2
SMC3mt #7
SMC3 acK105/106
SMC3
ATM pS1981
KAP1 pS824
RPA2 pS4/S8
CHK1 pS345
RPA2 pS33
γH2AX
H2AX
Actin

Figure 7F

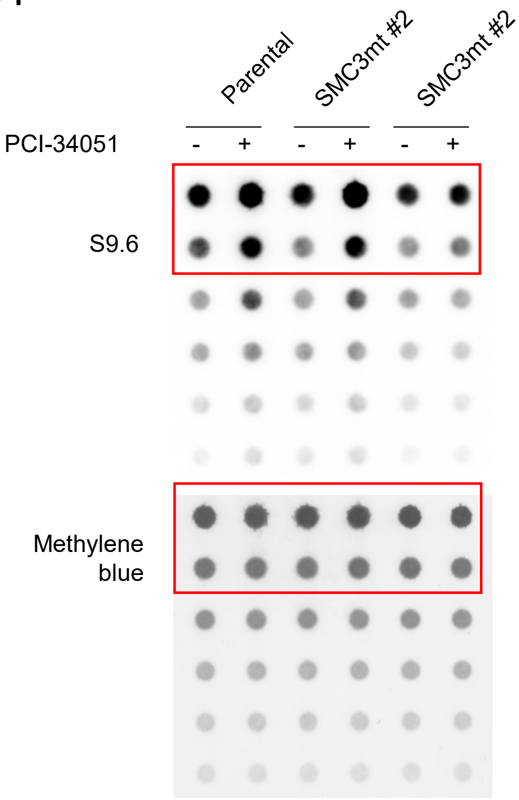

Supplementary Figure 1H

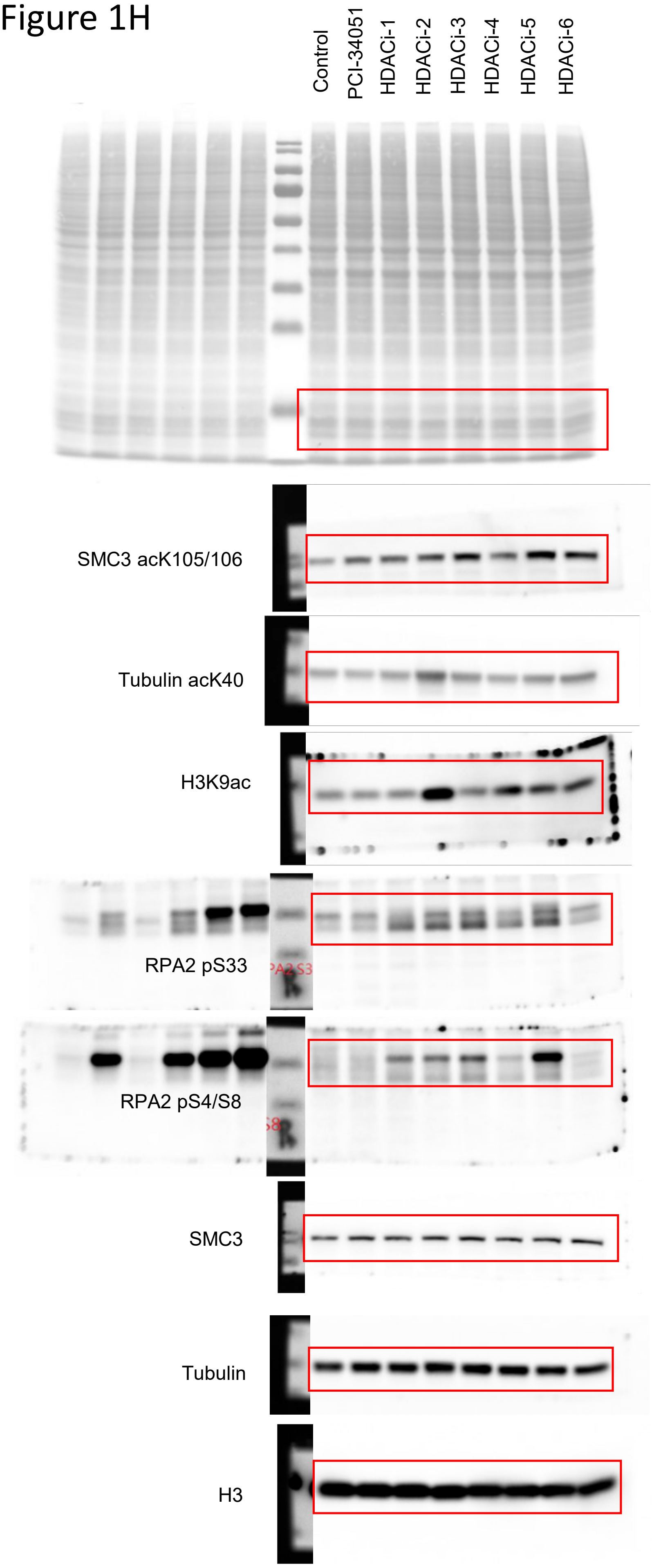

Supplementary Figure 2A

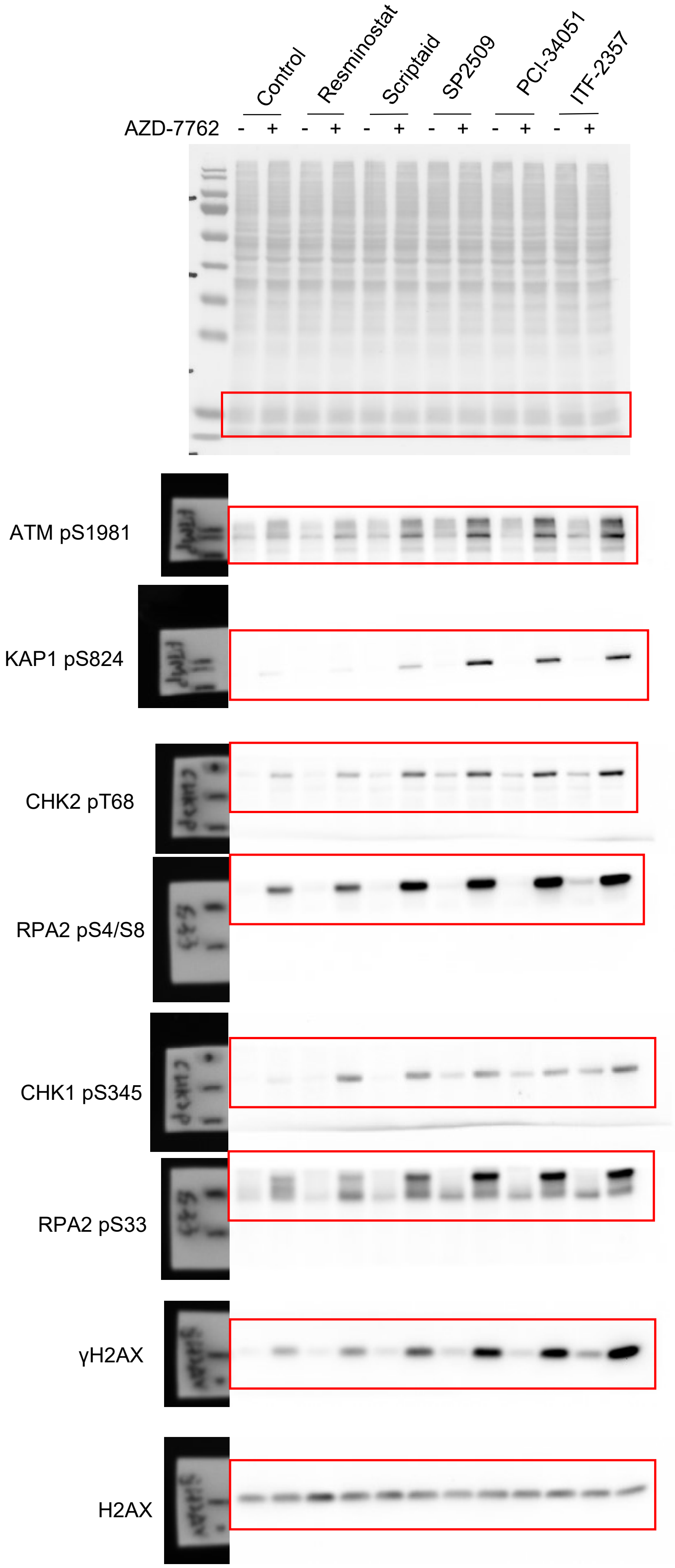

Supplementary Figure 2B

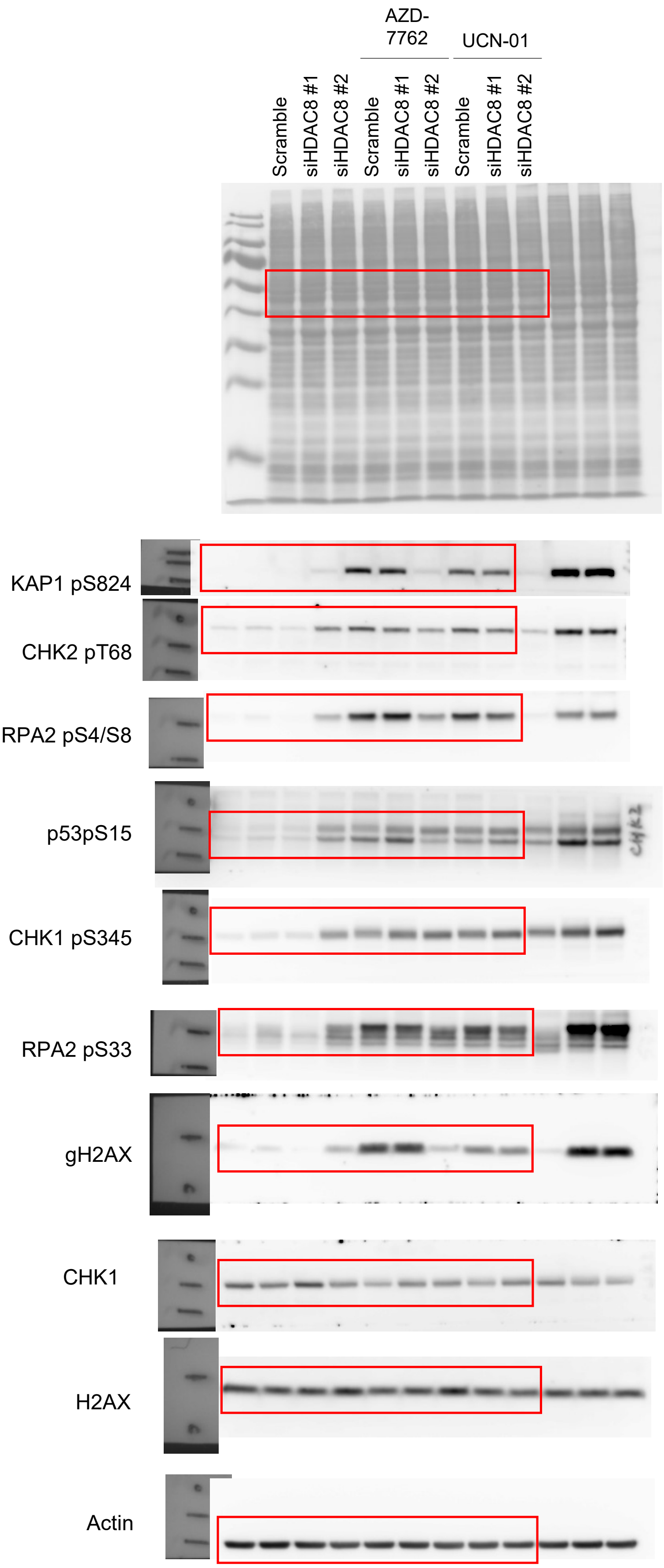

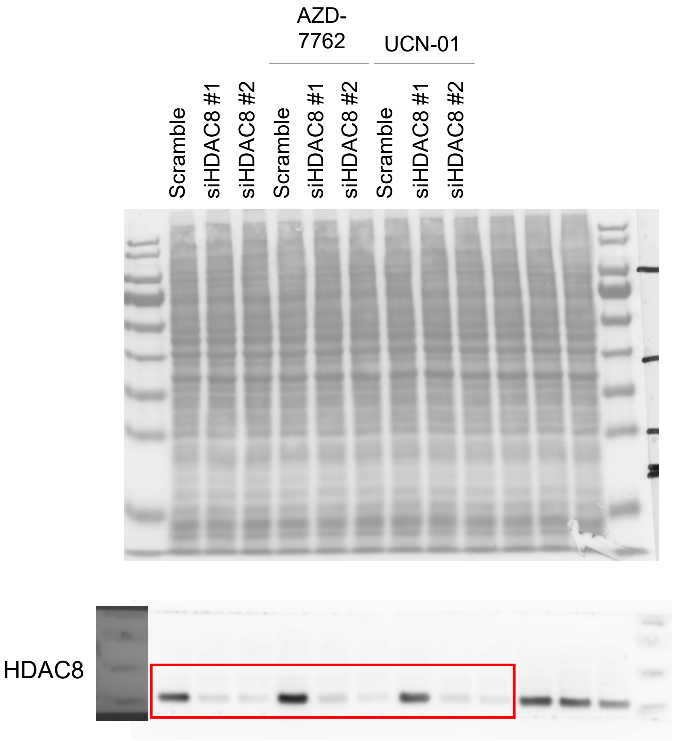

Supplementary Figure 2C

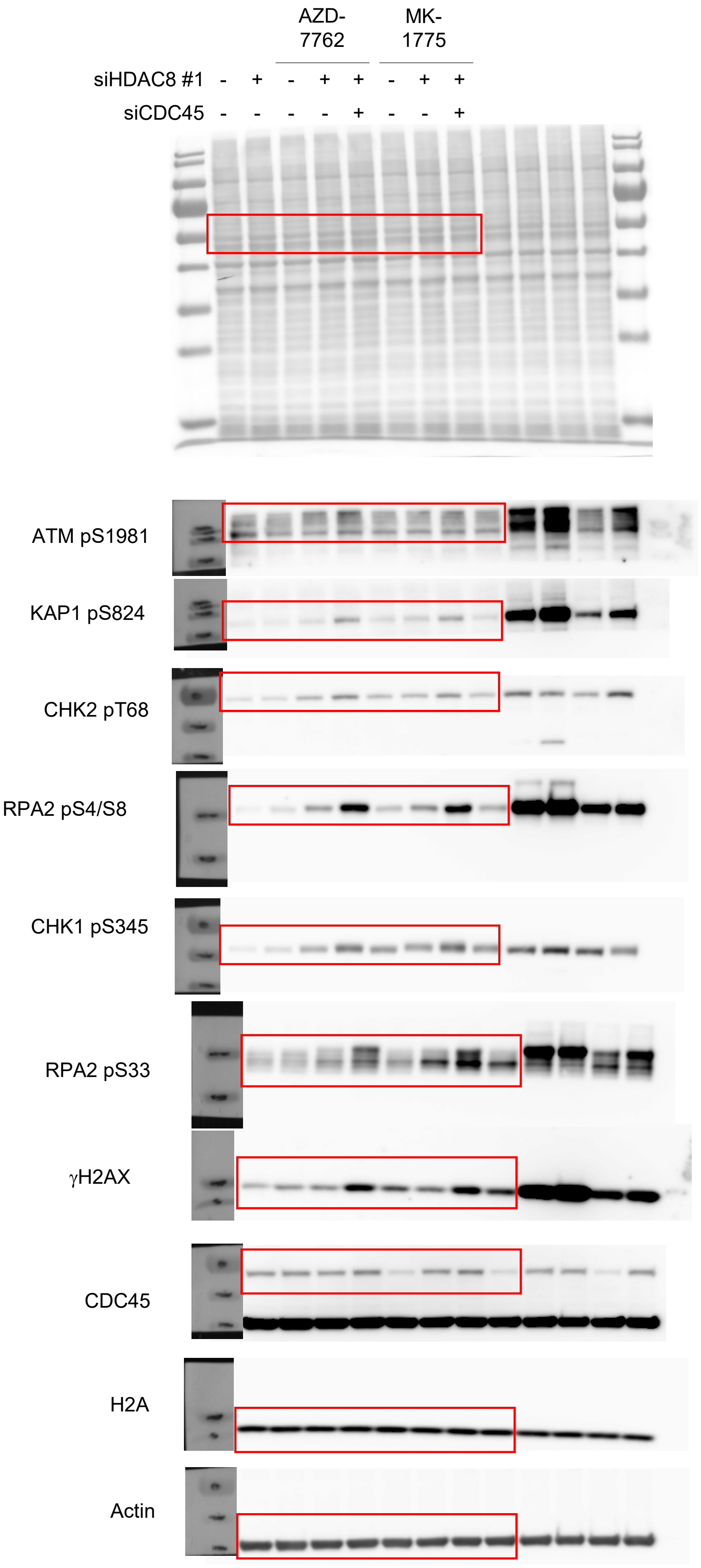

Supplementary Figure 2D

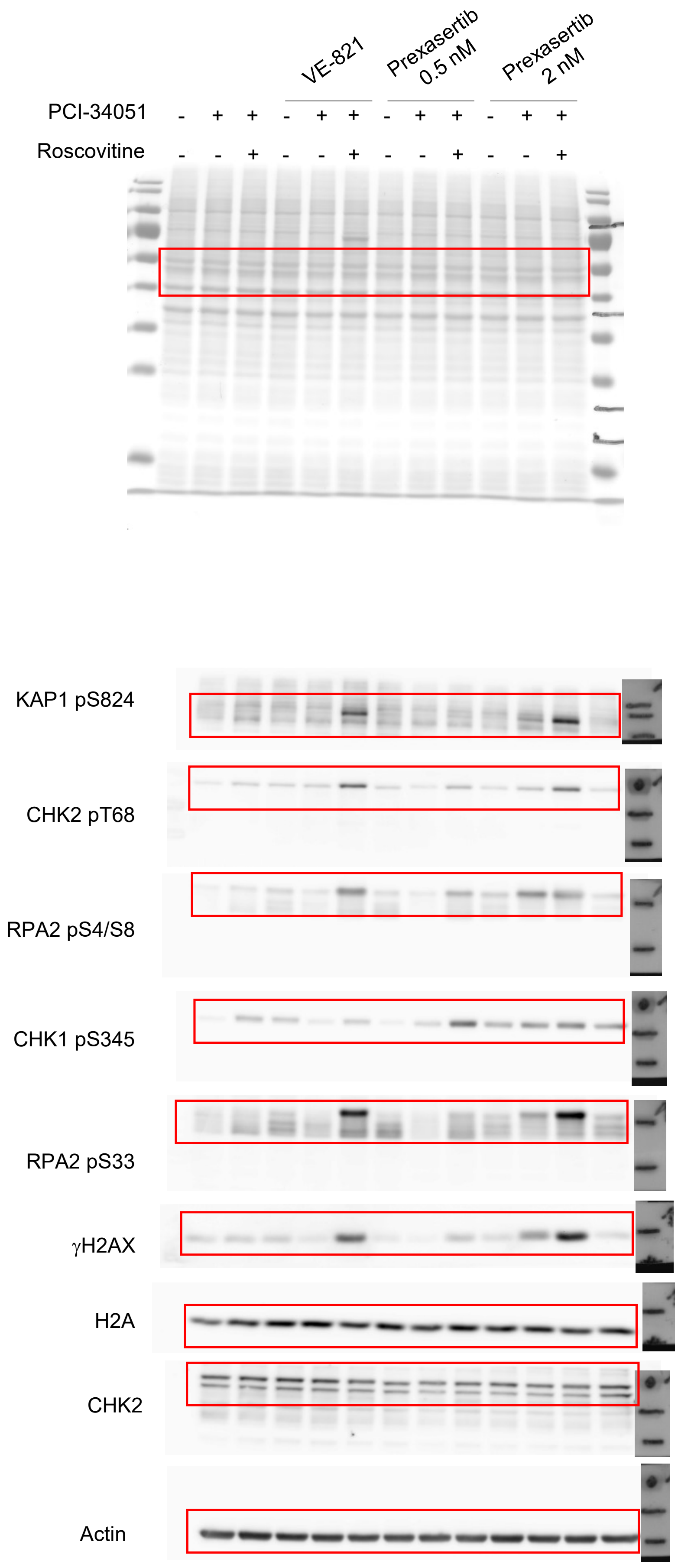

Supplementary Figure 2E

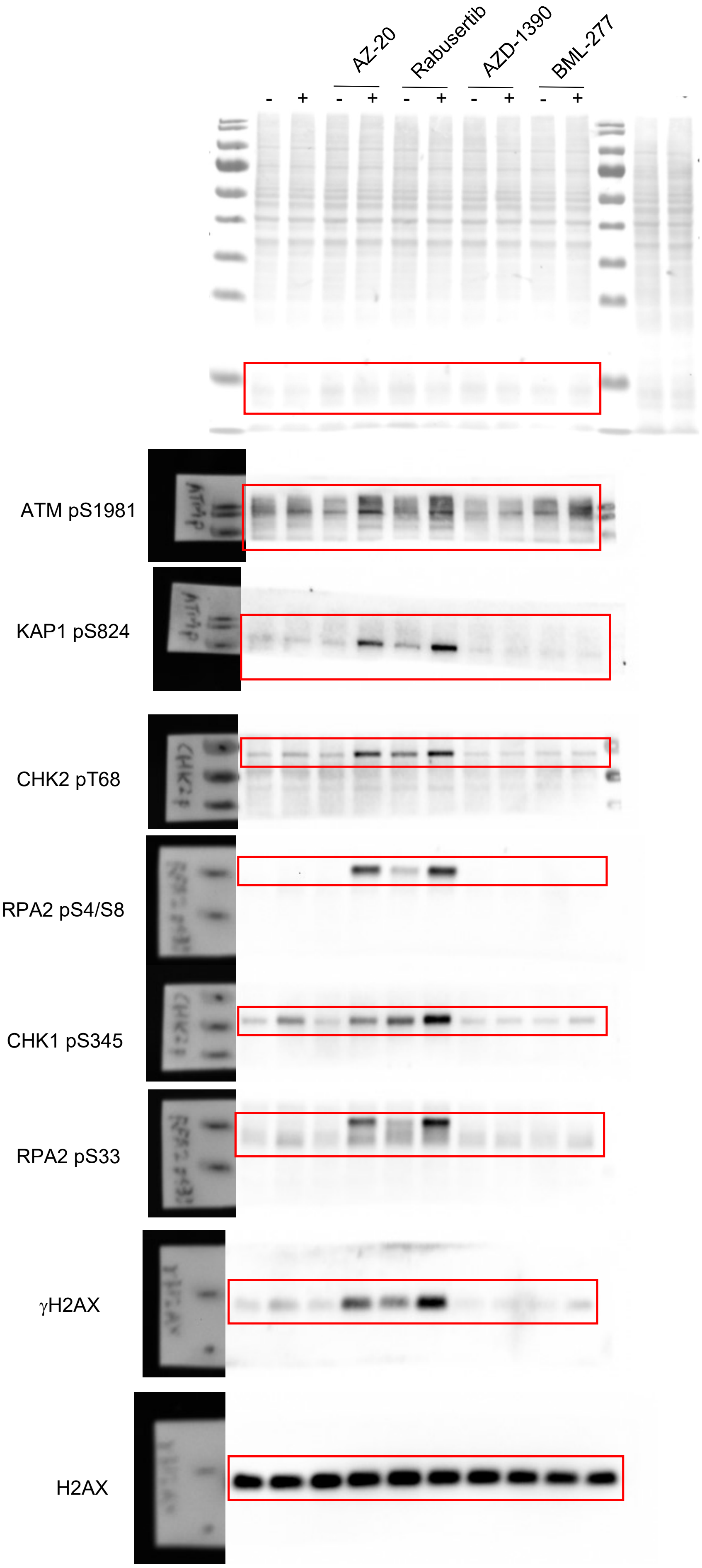

Supplementary Figure 4B

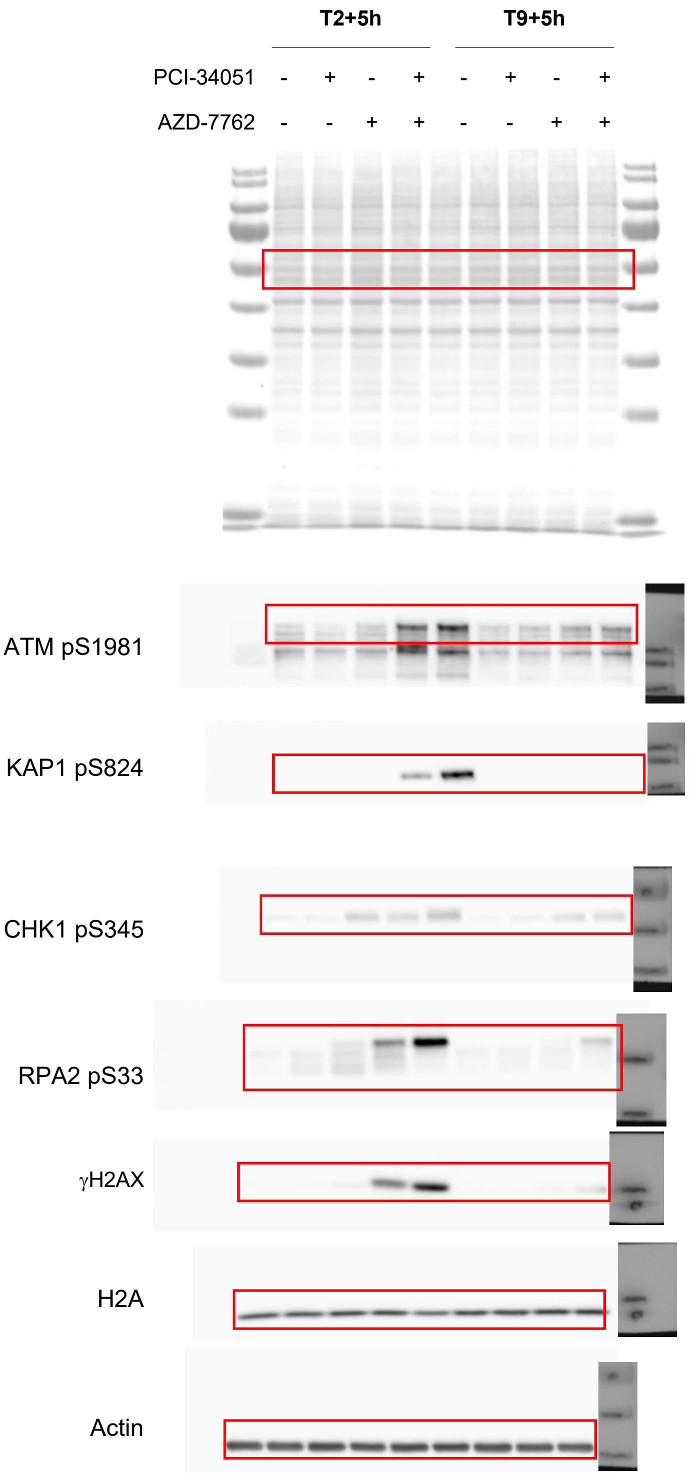

Supplementary Figure 6E

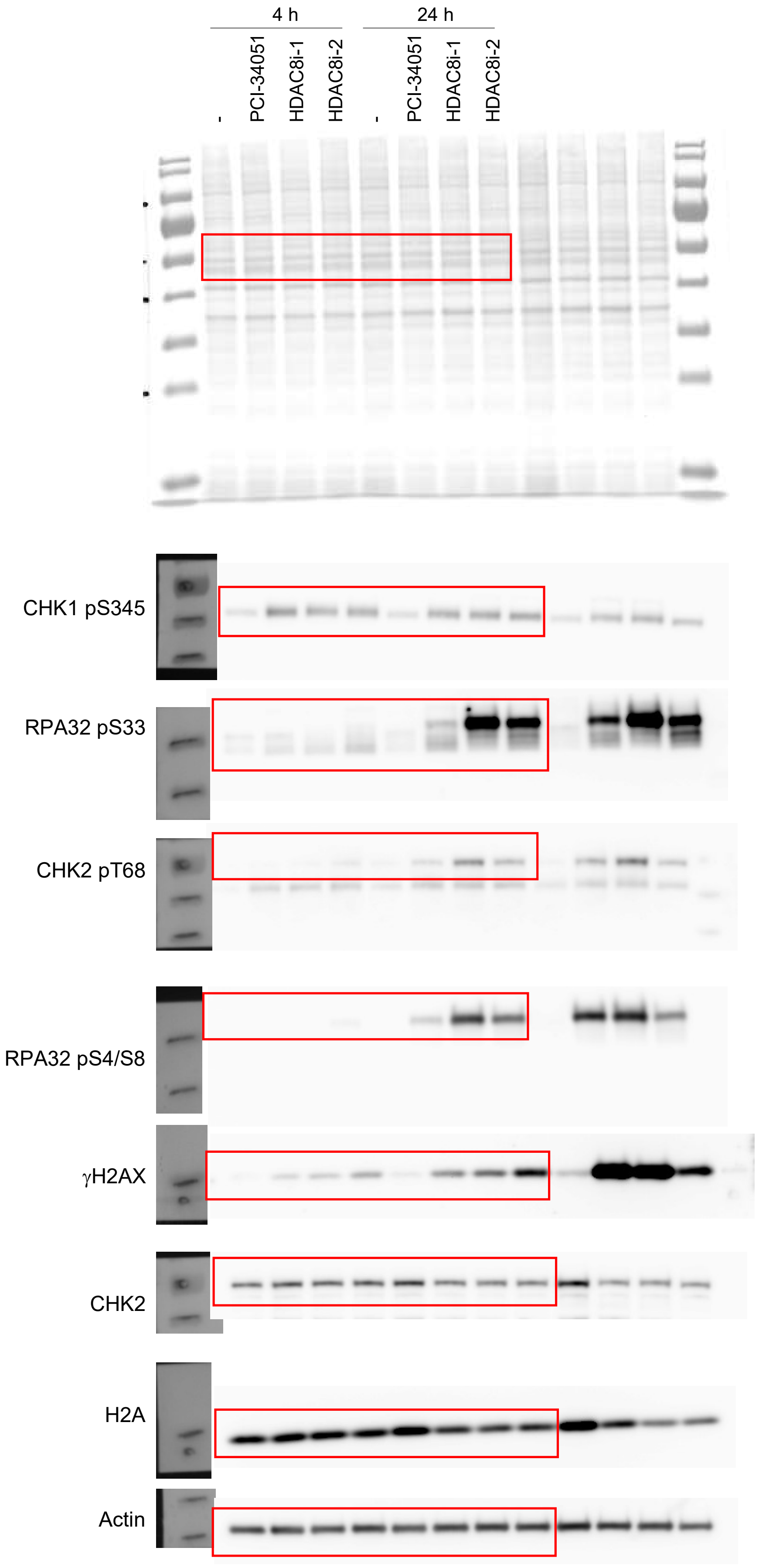

Supplementary Figure 6G

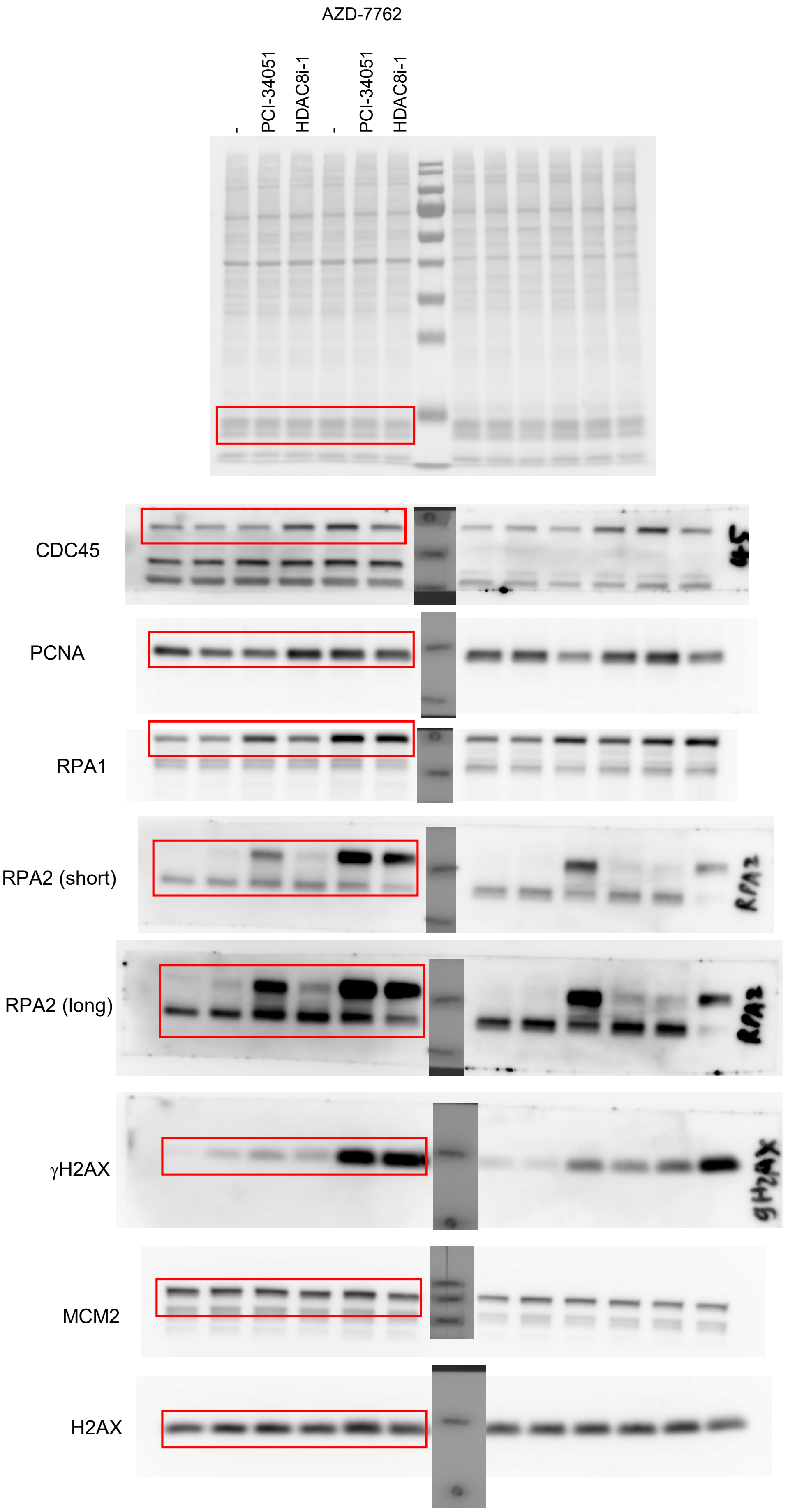

Supplementary Figure 7B

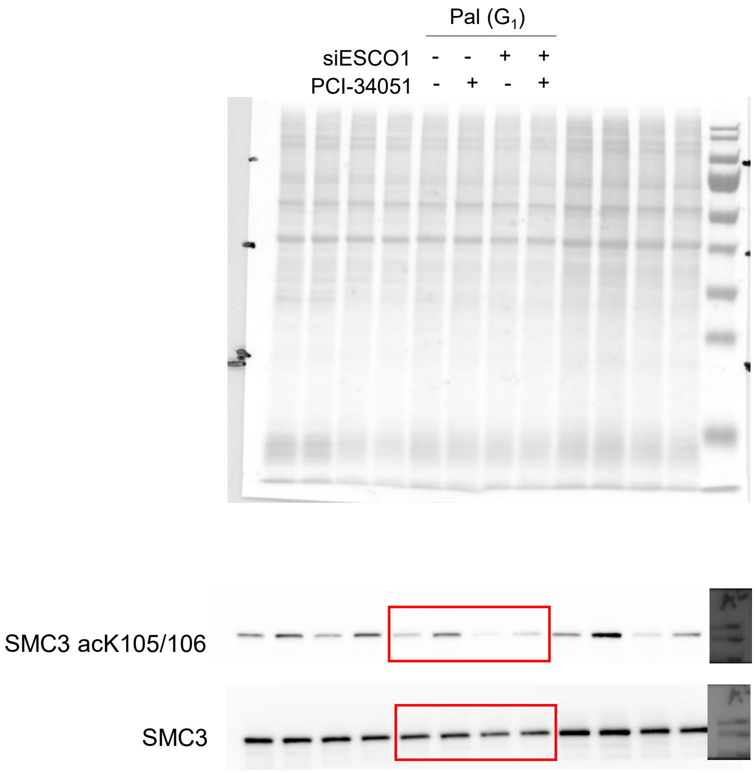

Supplementary Figure 8A

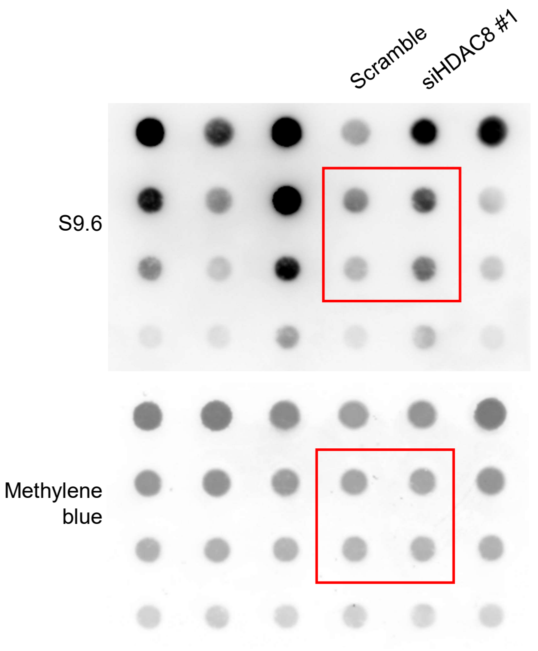

Supplementary Figure 8B

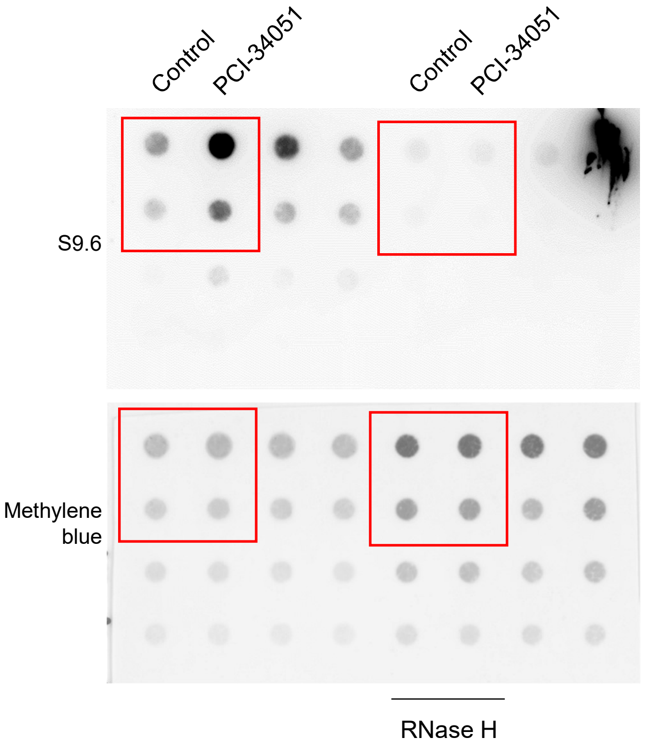

Supplementary Figure 8C

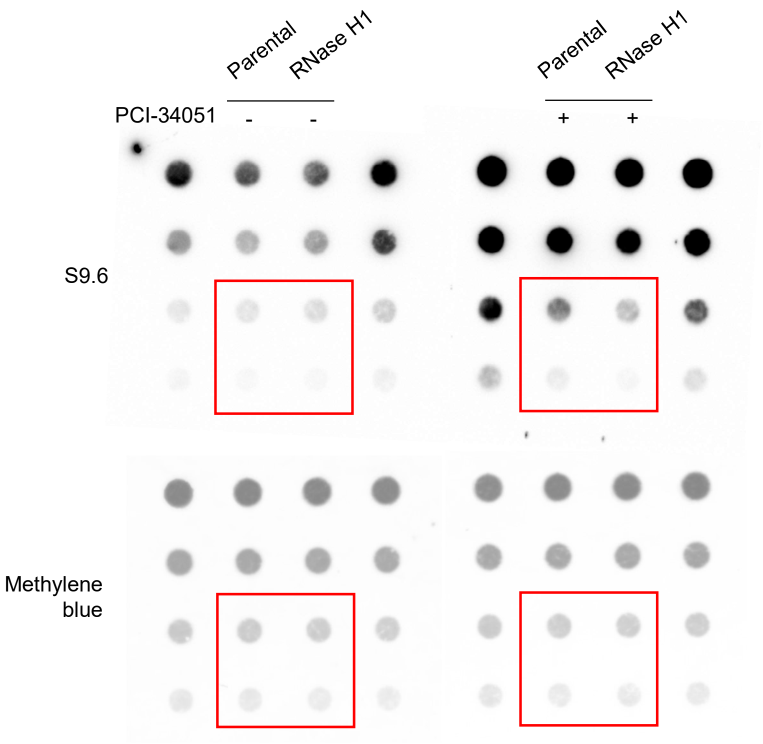

Supplementary Figure 8D

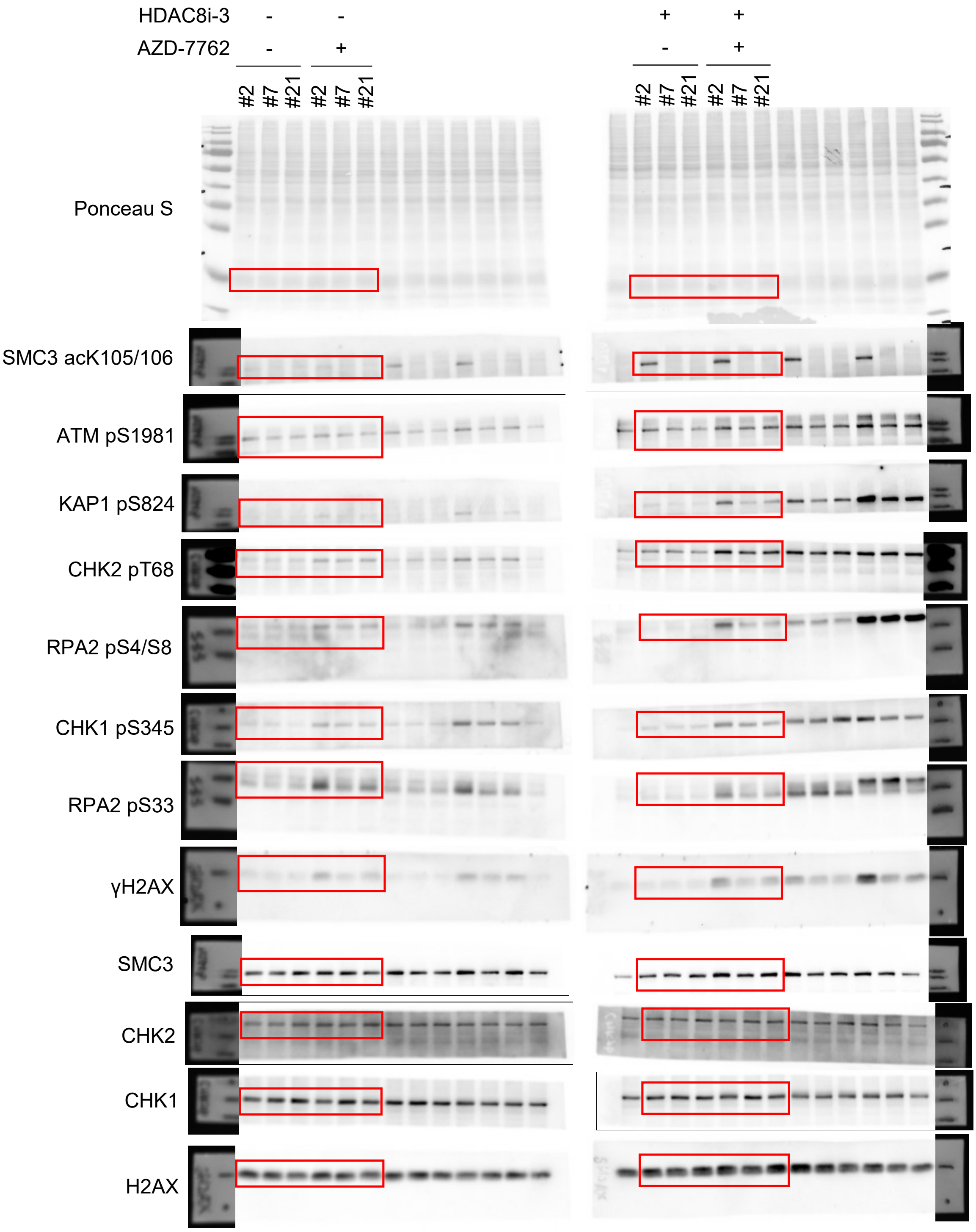

Supplementary Figure 8E

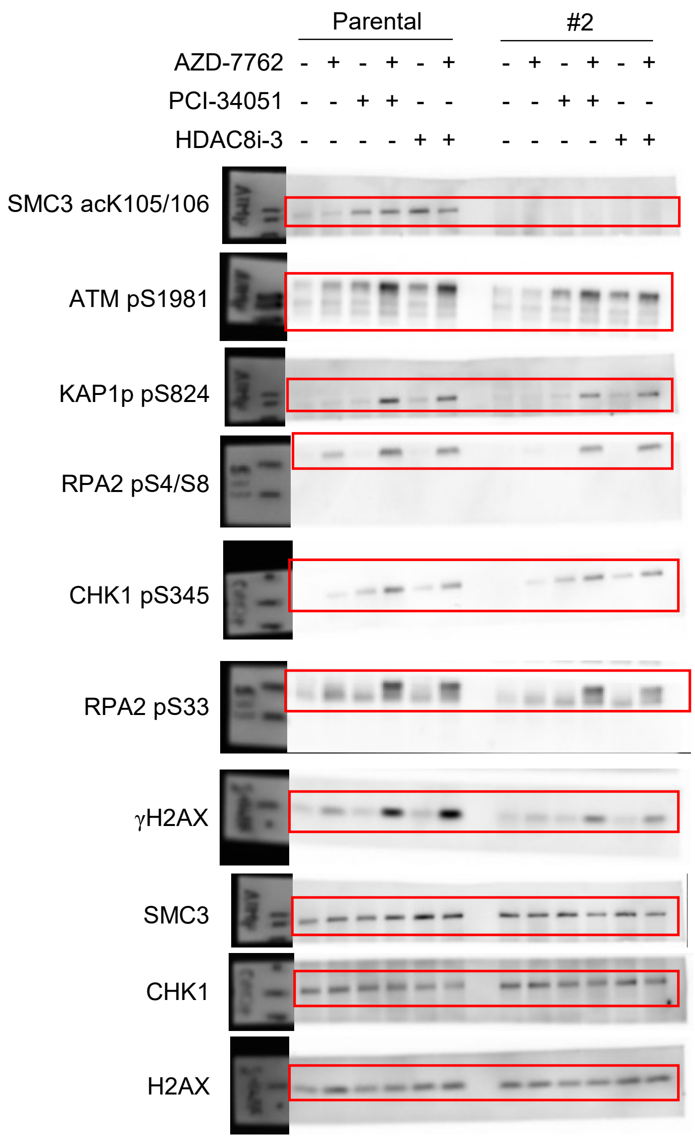

Supplementary Figure 8E

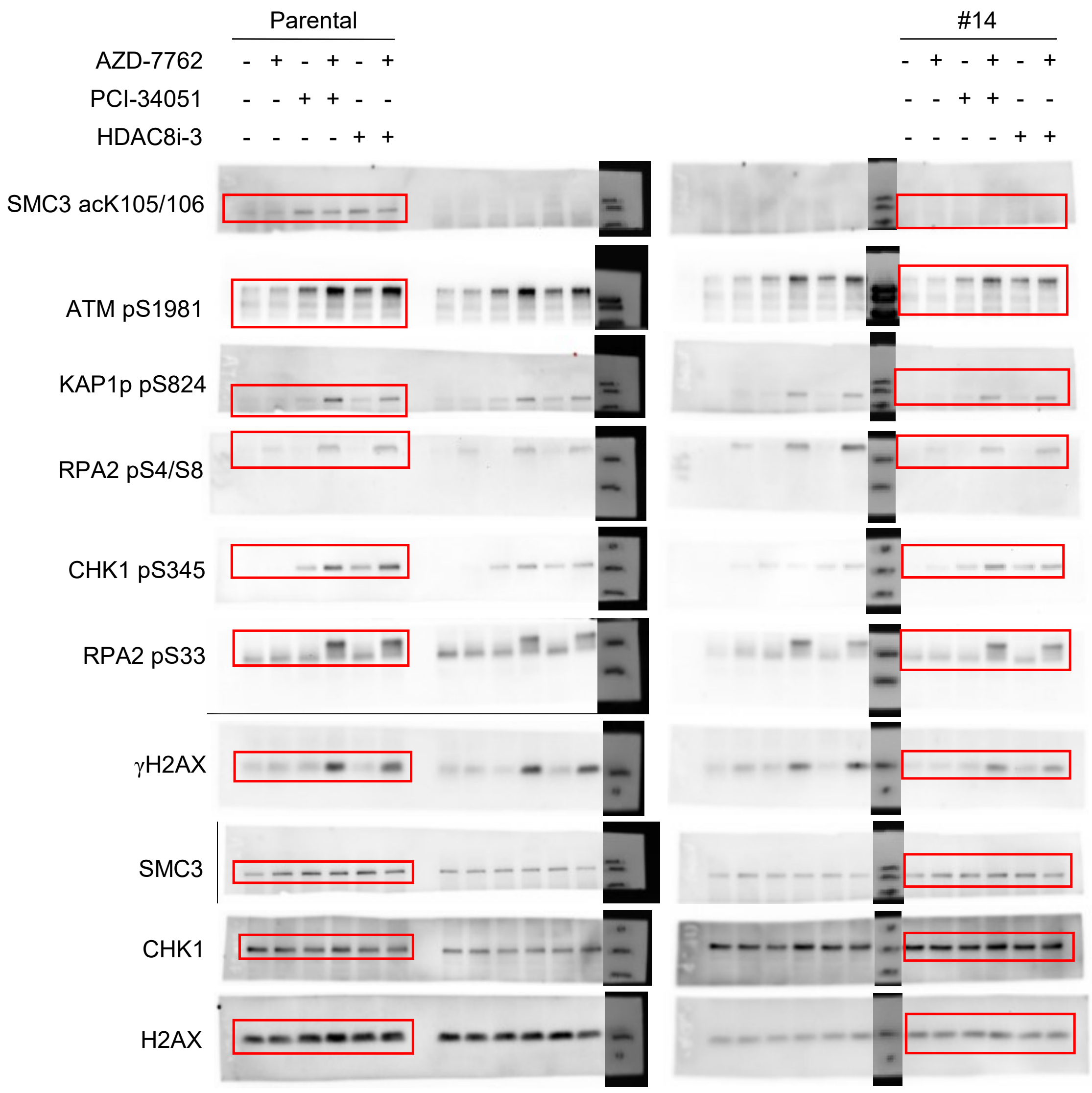

Supplementary Figure 8F

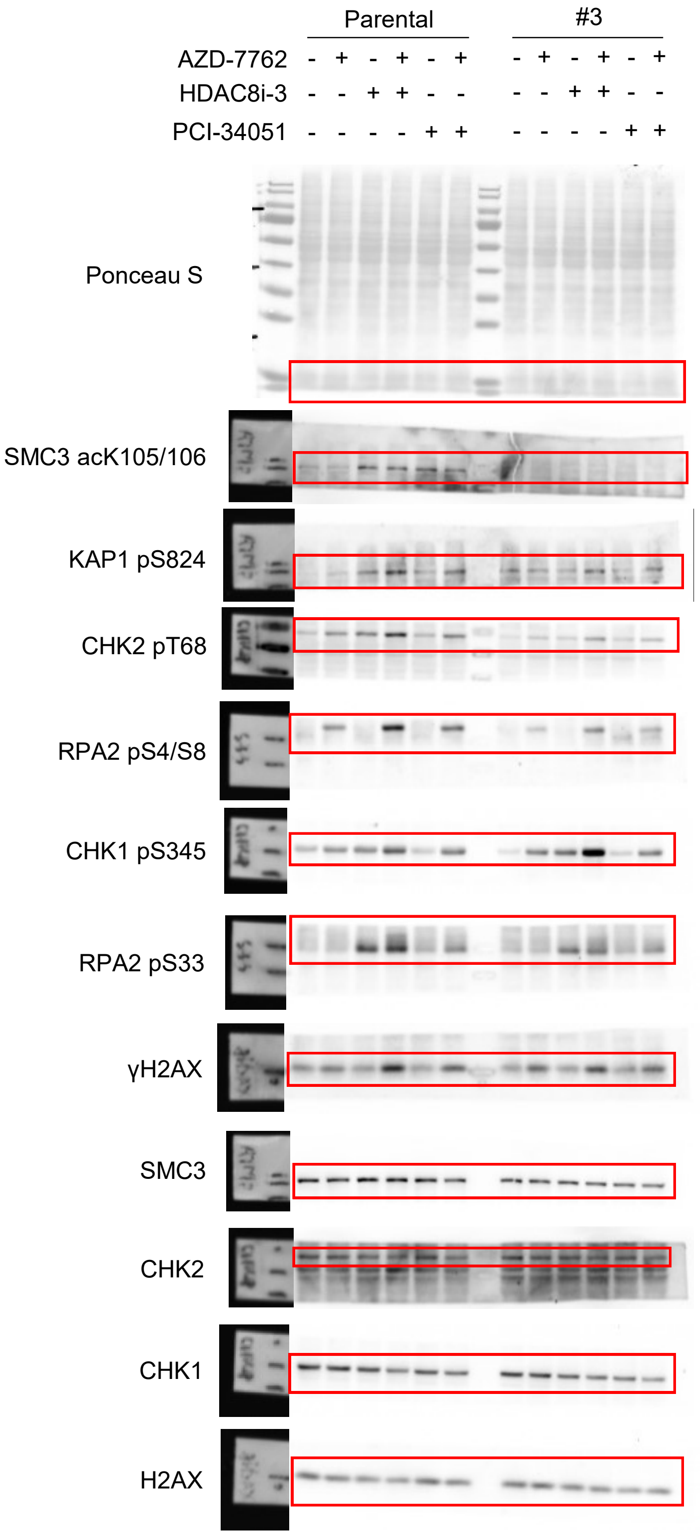

Supplementary Figure 8G

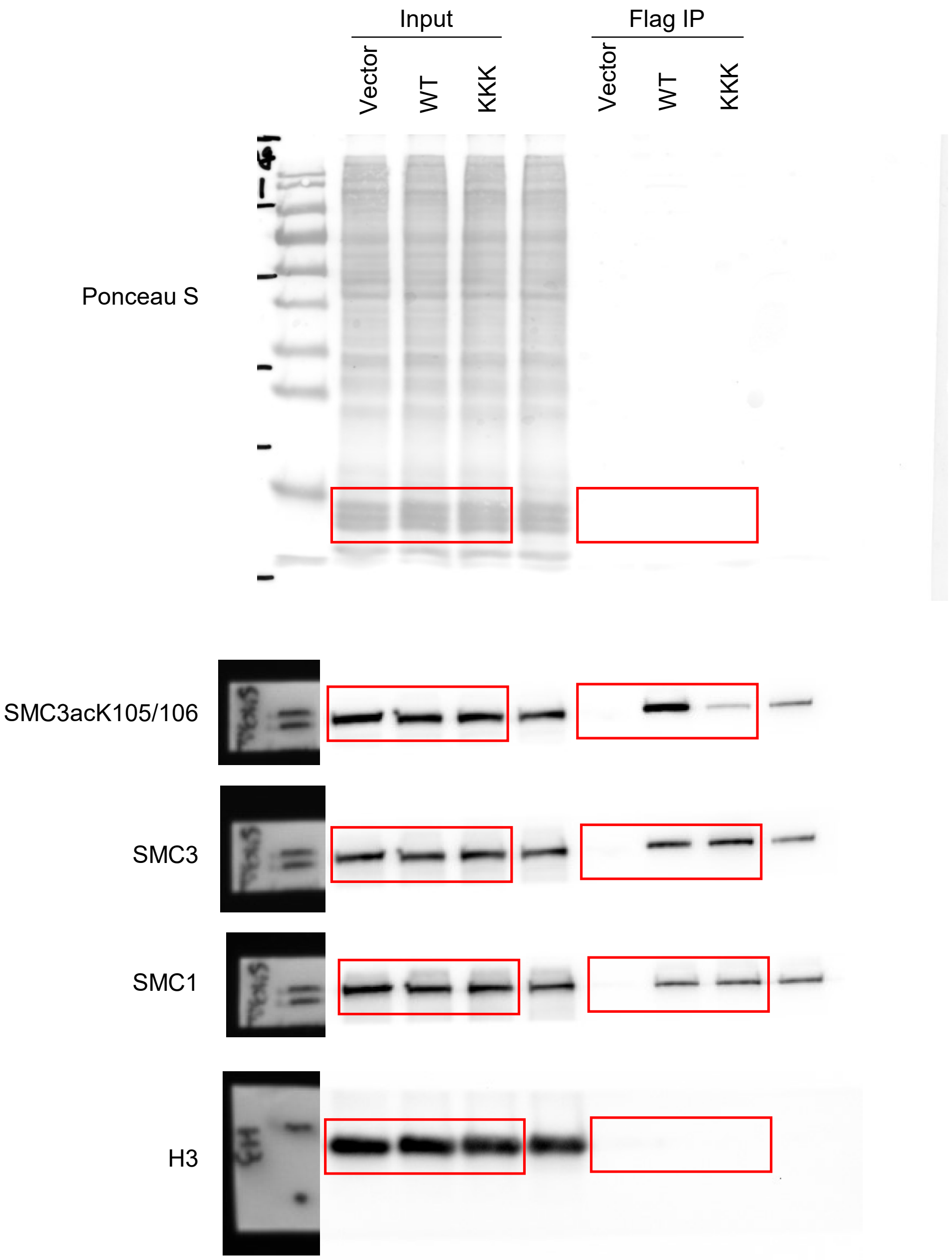

Supplementary Figure 10C

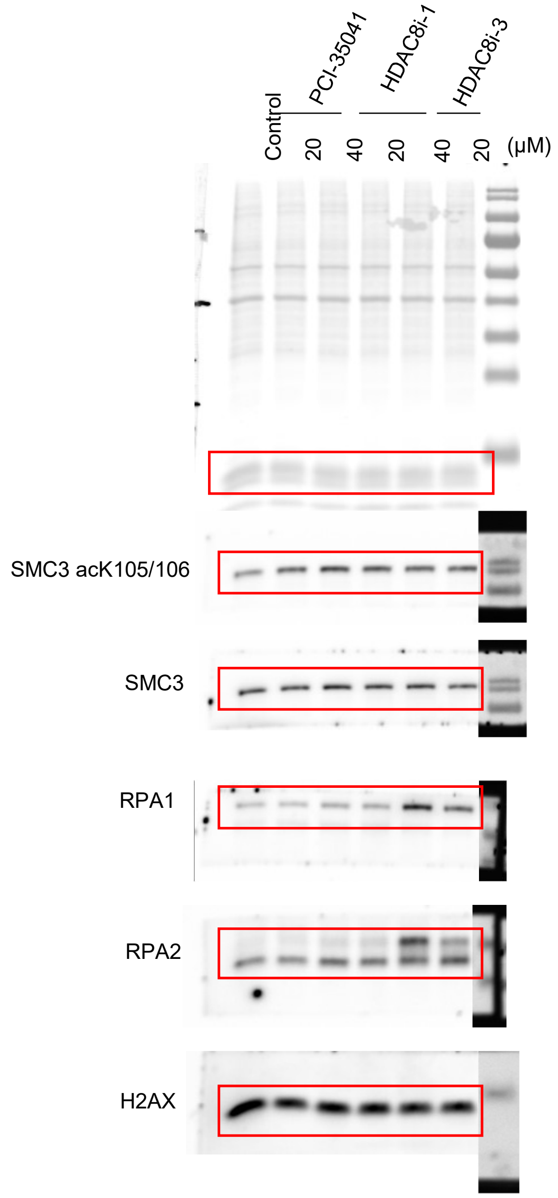

Supplementary Figure 10D

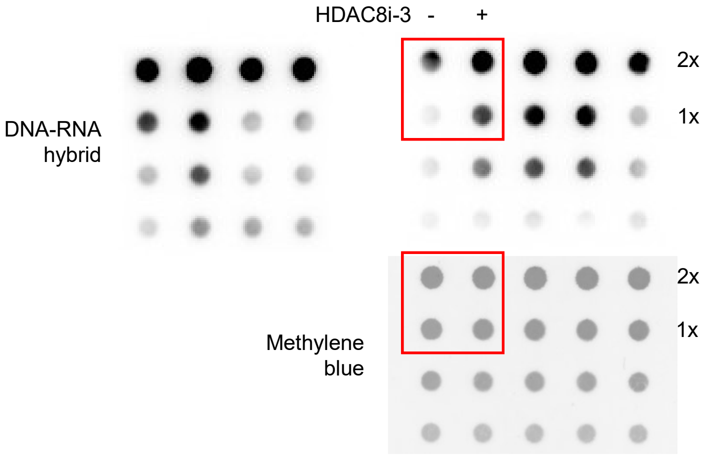

Supplementary Figure 10E

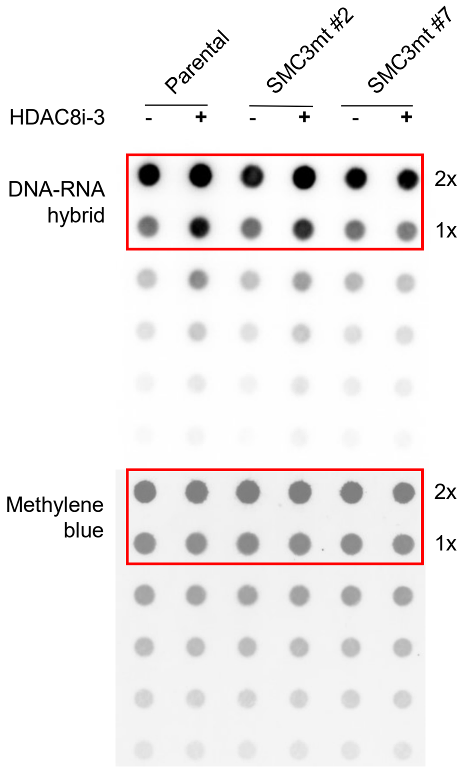

Supplementary Figure 10F

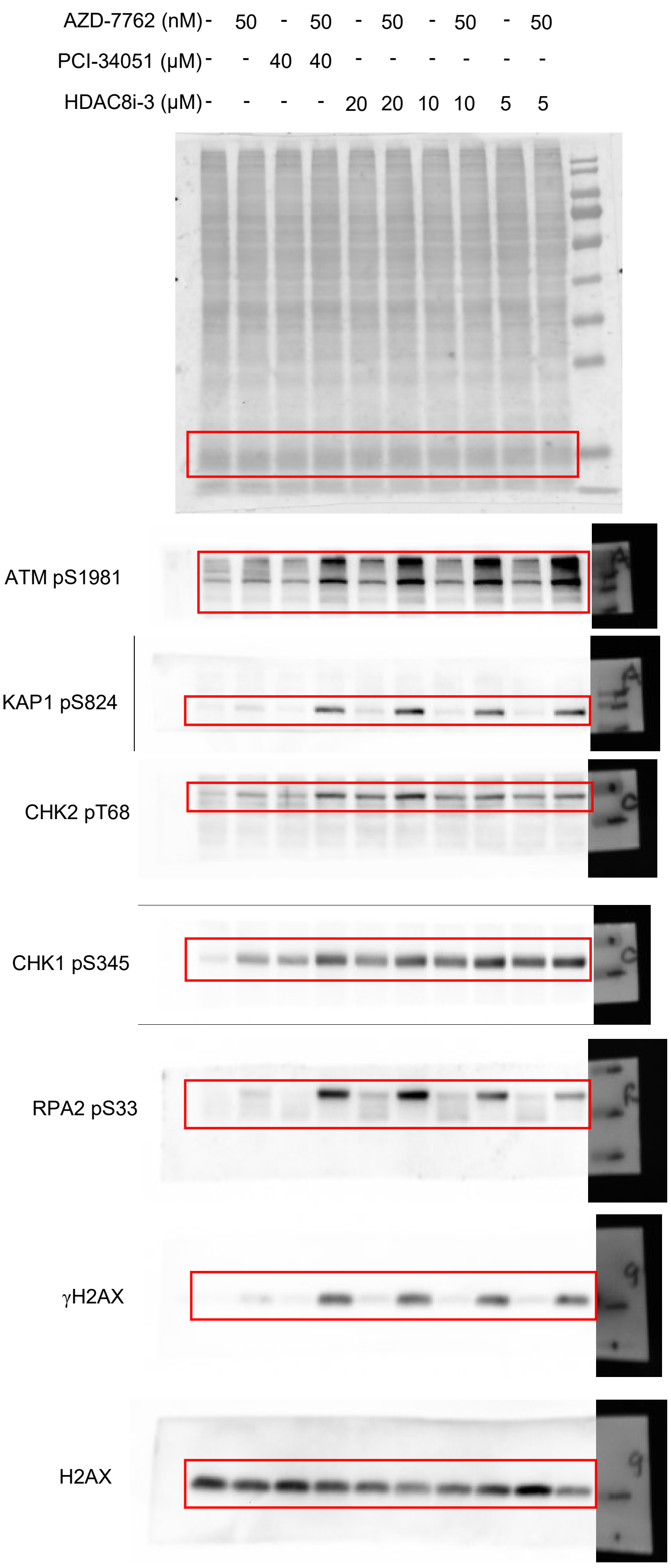

Supplement: Unedited blot and gel images [file jci-134-165448-s163.pdf]
